# Transparent and Transient Flexible Electronics

**DOI:** 10.1002/advs.202505133

**Published:** 2025-06-20

**Authors:** Nitheesh M. Nair, Ayoub Zumeit, Ravinder Dahiya

**Affiliations:** ^1^ Institute for Automation and Applied Informatics Karlsruhe Institute of Technology 76344 Eggenstein‐Leopoldshafen Germany; ^2^ Bendable Electronics and Sustainable Technologies (BEST) Group Electrical and Computer Engineering Department Northeastern University Boston MA 02115 USA

**Keywords:** flexible electronics, large‐area electronics, printed electronics, transient electronics, transparent electronics

## Abstract

Transparent electronics has gained tremendous attention in recent years because of the growing demand for see‐through devices in applications such as displays, windscreens, and wearables. These applications require transparent electronics in a large area and flexible form factors along with performances at par with conventional electronics. Additionally, the controlled transience and degradability of electronics are desired to reduce the end‐of‐life challenges. Attaining these attributes simultaneously is challenging as inherent material properties do not always align well, and there are technological limitations such as thermal budget issues in the case of flexible substrates. As a result, several materials and structures, including 1D nanowires, 2D nanosheets, metal oxides, and polymers etc., are explored. This comprehensive review discusses these developments related to transparent electronics as well as the challenges associated with the development of flexible and transient transparent electronics over large areas. Potential solutions to overcome these challenges and various resource‐efficient deposition and printing technologies are also presented along with examples of reported transparent circuits, sensors, actuators, and energy devices. Finally, potential future directions are discussed for flexible transient transparent electronics as their ever‐growing demand could lead to the emergence of new materials, fabrication techniques, and applications.

## Graphical abstract

Transparent electronics has gained tremendous attention in recent years because of the growing demand for see‐through devices in applications such as displays, windscreens, and wearables. This review discusses the recent developments related to transparent electronics as well as the challenges associated with the development of flexible and transient transparent electronics over large areas.

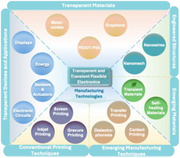

## 1 Introduction

Transparent devices that are imperceptible to human eyes are attracting considerable interest for emerging see‐through applications such as smart windows, vehicle windscreens, wearables, eyewear, optical communications using light fidelity (Li‐Fi), and many more.^[^ ^1^ ^]^ They could allow the user to simultaneously see the information projected on a display and the real world in the background. By incorporating transparent touch panels, it is possible to make interactive displays too. In fact, such approaches have been explored for touch interactive haptic displays, energy generation and storage with touch‐sensing capabilities, and self‐powered electronic skin in robotics.^[^ ^2^ ^]^ Such transparent electronic systems have massive potential to augment reality, head‐up displays in automobiles, signboards, and holographic information displays for health, military, and educational purposes.^[^ ^3^ ^]^ Likewise, a transparent solar cell can convert any windowpane into an invisible source of energy generation, which can be stored locally via a transparent supercapacitor or other storage cells.^[^ ^4^ ^]^ Transparent electronics also have high potential in healthcare wearables, such as see‐through smart wound plasters, to monitor the healing without damaging the tissues.^[^ ^5^ ^]^

The optical transparency of various materials used for transparent electronics plays a crucial role in achieving the required performance and ensuring practical implementations. A device must have all optically transparent layers, including the substrate, active layers, contacts, and dielectric. At the same time, the transparent devices should possess excellent electrical properties. For example, in addition to the optical transparency, a transparent antenna needs highly conducting patches and a substrate with optimum dielectric constant and low loss‐tangent to efficiently convert the electrical current to electromagnetic radiations and vice‐versa. Attaining good charge carrier density for metallic and semiconducting layers, while allowing visible light to pass through without getting absorbed, is a highly arduous task as more charge carriers favor higher light absorption. Several emerging applications also require mechanically flexible devices that need to be fabricated over large areas and provide uniform performance. Further, the push for degradable devices is gaining momentum, because of rapidly growing end‐of‐life issues such as electronic waste management.^[^ ^6^ ^]^

There are multiple approaches to achieving optical transparency in various electronic materials. Metal oxides, such as indium tin oxide (ITO), are inherently transparent due to their wide bandgap, and degenerate doping is explored to improve their electrical properties. However, being ceramic, they are highly brittle and hence not ideal for flexible electronics applications.^[^ ^7^ ^]^ On the other hand, polymers such as PEDOT:PSS,^[^ ^1^, ^8^ ^]^ and 2D materials such as graphene^[^ ^9^ ^]^ offer good flexibility and can exhibit low optical absorption at low thickness. They can be deposited as a continuous, homogenous thin film to realize the transparent electronics. However, their electrical performance needs to be boosted, particularly for high‐performance electronic devices.^[^ ^1^, ^10^ ^]^ Materials with high optical absorption, such as Ag, Cu, and Si, can also be structured as grids, meta‐surfaces, and nanostructures to obtain optically transparent films with optoelectronic properties comparable with vacuum‐deposited metal oxides.^[^ ^11^ ^]^ The optical transparency of substrate and passivation layers are also important since they can affect the overall device transparency and performance of optoelectronic devices.^[^ ^12^ ^]^

Along with excellent optoelectronic properties, it is crucial to consider the ecological footprint of the manufacturing processes used to obtain transparent materials and devices and the end‐of‐life management of electronics developed from them. Some of the materials critical for transparent electronics are scarce. Traditional fabrication methods, such as lithography and etching, are resource‐intensive and inherently wasteful—generating substantial material waste and releasing harmful chemicals into the wastewater and environment. For instance, indium, a key component in ITO, is a rare‐earth element and increasing demand for indium‐based films in optoelectronic applications has driven up the costs of ITO (>90 at% of the cost is dedicated for indium). Such situations emphasize the urgency for having manufacturing processes that generate minimal material wastage.^[^ ^13^ ^]^ Moreover, current patterning techniques for ITO often require the use of hazardous wet and chemical etchants, which pose significant environmental concerns.^[^ ^14^ ^]^ To address these challenges, resource‐efficient and ecologically benign manufacturing techniques such as printed electronics are being explored to deposit and pattern thin films for large‐area electronics.^[^ ^15^ ^]^ Such innovative approaches, as well as room temperature processing and the use of easily available materials, are vital for minimizing the ecological footprint of transparent electronics. The advent of transient electronics, designed to degrade into useful by‐products, could also offer a promising direction for transparent electronics that results in near zero electronic waste (e‐waste) after an operational life span.^[^ ^1^, ^16^ ^]^

**Figure 1:**
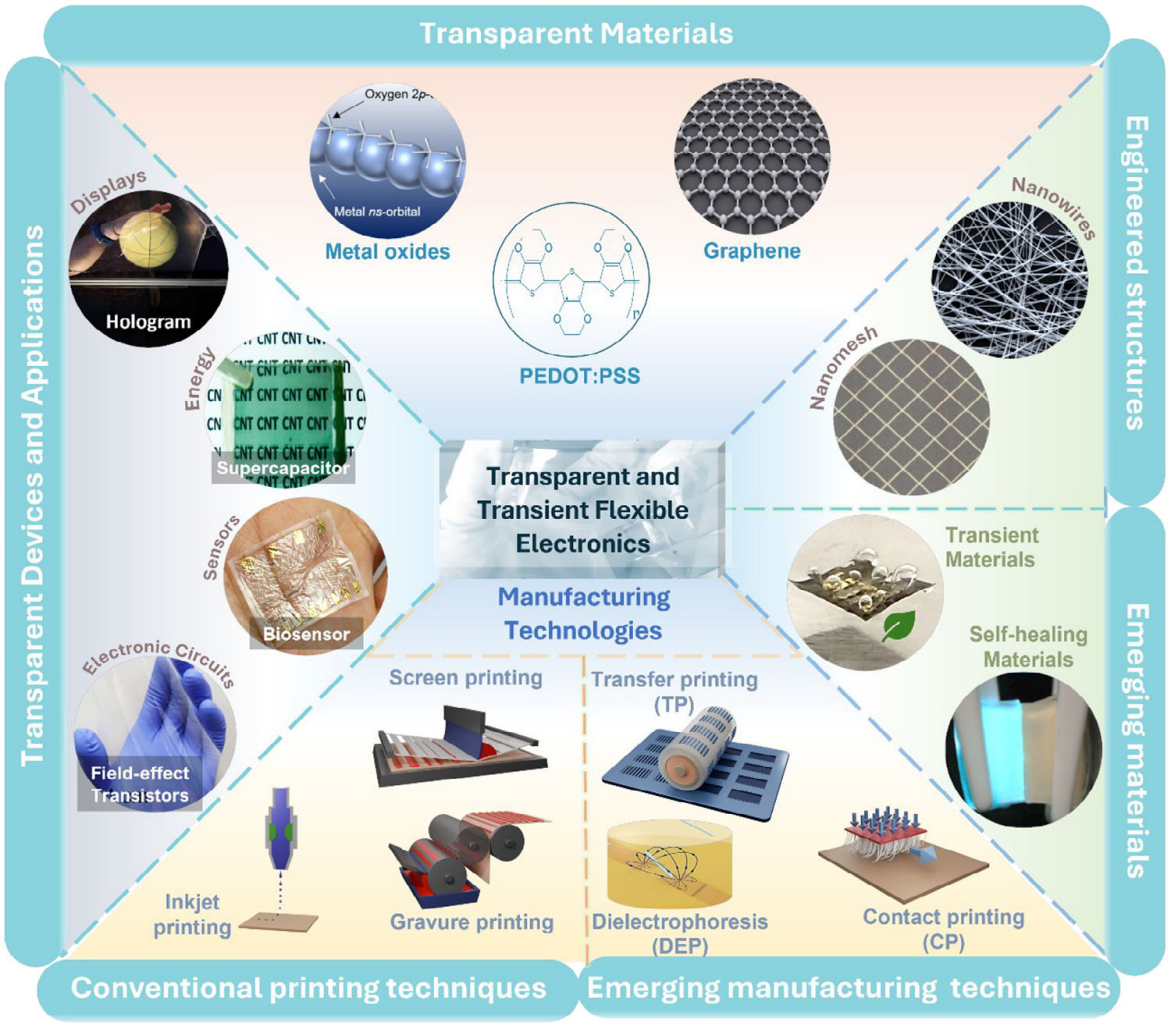
Overview of the materials, engineered structures, manufacturing technologies, and device‐level applications relevant to large‐area, flexible, and transient transparent electronics. “Metal oxides”: Reproduced with permission.^[^ ^20^ ^]^ Copyright 2004, Springer Nature. “PEDOT:PSS”: Reproduced with permission.^[^ ^21^ ^]^ Copyright 2004, Elsevier. “Graphene”: Reproduced with permission.^[^ ^22^ ^]^ Copyright 2016, American Chemical Society. “Nanowires”: Reproduced with permission.^[^ ^23^ ^]^ Copyright 2018, Elsevier. “Nanomesh”: Reproduced with permission.^[^ ^24^ ^]^ Copyright 2013, American Chemical Society. “Self‐healing materials”: Reproduced with permission.^[^ ^25^ ^]^ Copyright 2020, Springer Nature. “Transient materials”: Reproduced with permission.^[^ ^26^ ^]^ Copyright 2022, John Wiley and Sons. “Transfer printing (TP)”: Reproduced with permission.^[^ ^2f^ ^]^ Copyright 2024, AIP Publishing. “Contact printing (CP)”: Reproduced with permission.^[^ ^27^ ^]^ Copyright 2021, Springer Nature. “Dielectrophoresis (DEP)”: Reproduced with permission.^[^ ^28^ ^]^ Copyright 2023, IEEE. “Holographic displays”: Reproduced under the terms of the CC‐BY license.^[^ ^3b^ ^]^ Copyright 2020, John Wiley & Sons. “Supercapacitors”: Reprinted with permission.^[^ ^29^ ^]^ Copyright 2015, American Chemical Society. “Bioelectronic sensors”: Reproduced under the terms of the CC‐BY license.^[^ ^30^ ^]^ Copyright 2024, John Wiley and Sons. “Field‐Effect transistors”: Reproduced with permission.^[^ ^31^ ^]^ Copyright 2020, American Chemical Society.

This paper presents a comprehensive review of the above progress and future prospects of transparent flexible large‐area electronics. A summary of the main directions, such as commonly used materials, emerging resource‐efficient fabrication techniques, and potential applications of transparent flexible electronics, is given in **Figure** **1**. A few of these directions, such as transparent materials,^[^ ^17^ ^]^ conventional fabrication techniques,^[^ ^10^, ^18^ ^]^ and applications,^[^ ^19^ ^]^ have also been reviewed independently in the past. However, this review article is distinct because of several new and emerging aspects, such as resource‐efficient fabrication techniques, and transience studies related to flexible and transparent electronics. The coverage of such topics is timely, considering the renewed focus on semiconductor manufacturing and the emergence of new challenges such as the management of e‐waste. In fact, as mentioned above, the rapidly growing issues, such as management of e‐waste, are acting as the catalyst for advances such as transient (controlled degradation) transparent electronics. A few examples of transient transparent electronics, presented in this paper, clearly indicate these developments. In fact, these new topics make this review article complementary to the previously reported reviews on transparent electronics. The diverse, and often conflicting, requirements of flexible transparent electronics reported here show how challenging it is to explore this topic. At the same time, the wide‐ranging applications of transparent electronics make this field exciting too. It is hoped that such new topics will benefit researchers and practitioners from academia and industry interested in the field of transparent electronics.

The review begins with a discussion of the various materials popular for transparent electronic applications, such as conventional metal oxides, polymers, recently popular carbon‐based and metallic nanostructure‐based materials, and their hybrids. We then introduce the advanced manufacturing and printing techniques to pattern and deposit the transparent flexible material for large‐area fabrication, and their advantages and disadvantages. After this, some emerging applications in devices and circuits, sensors, actuators, energy, etc. are discussed. Finally, we discuss various challenges and future directions in the area.

## 2 Historical Development

The transparent electronics began to take shape when conductive Cd film, grown by evaporation in 1907, was observed to become transparent after oxidizing in air, while maintaining conductivity.^[^ ^32^ ^]^ The formed CdO film is the first‐ever reported transparent and conductive material. The initial developments were engrossed with binary and ternary oxides of indium, tin, and zinc. But, in the last two decades, many new materials such as metallic nanostructures, polymers, 2D materials, and composites have also emerged. Advancements in fabrication techniques, moving away from complex vacuum deposition to cost‐effective and resource‐efficient solution‐processed and hybrid technologies, such as printing, make it possible to integrate transparent electronic material with high performance at relatively low processing temperatures of <100 °C. These fabrication advancements fueled the concept of flexible and transparent electronics, which is otherwise difficult to achieve with the conventional approach of making flexible devices by thinning the conventional Si wafers, like the first flexible solar cell reported in 1967 and later for flexible chips.^[^ ^33^ ^]^ In the last decade, developments in amorphous oxides, as well as atmospheric pressure low‐temperature fabrication routes, have made it possible to think beyond rigid glass to plastic substrates that are flexible, stretchable, and conformable. The plastic substrates are attractive for portable device applications because they are lightweight, thinner, and more rugged than glass and compatible with reel‐to‐reel processing, making them suitable for cost‐effective manufacturing over a large area.^[^ ^34^ ^]^

**Figure 2:**
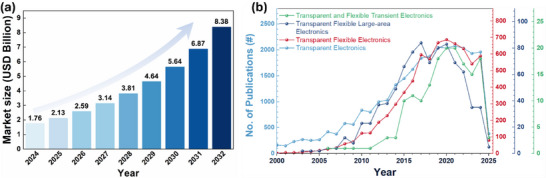
Market and Research trends. a) Transparent electronics market size predicted by Precedence Research. (Source – www.precedenceresearch.com) b) Number of articles published having keywords “flexible”, “large area”, “transient”, and “transparent electronics” (Source – Web of Science, March 2025).

We have studied the market demand and the research trend in the field of transparent electronics. The technological reports, such as the one prepared by Precedence Research (**Figure** **2**a), show that the transparent electronics industry generated 1.76 billion USD in 2024 and is predicted to create 8.38 billion USD by 2032, with a Compound Annual Growth Rate (CAGR) of 21.5% during the period. The growing market demand for transparent devices can be attributed to their applications in the areas of consumer electronics, automotive, energy, healthcare, and other industries, with innovative and sustainable solutions. The academic and research sectors also experienced exponential growth, as may be noted from Figure 2b, which shows the number of research articles published on topics related “transparent and flexible electronics”, during most of the last two decades. However, there has been a slight decrease in the number of publications during the last 4 years, which underlines the need for more creative solutions to meet the market demand. Among the total publications in the year 2024 on “transparent electronics”, 30% have the term “flexible”, and 2% have “large area”, which means all transparent electronics are not flexible and large‐area electronics. From a sustainability perspective, it is concerning that less than 1% of the published articles included the term “transient”. Nonetheless, the field has picked up in recent years and significant opportunities lie ahead for advancements in the field of transient transparent electronics. The materials and emerging manufacturing techniques, reviewed in the later sections, could be deployed to attain advances in sustainable transparent electronics.

## 3 Strategies for Attaining Transparency in Electronics

**Figure 3:**
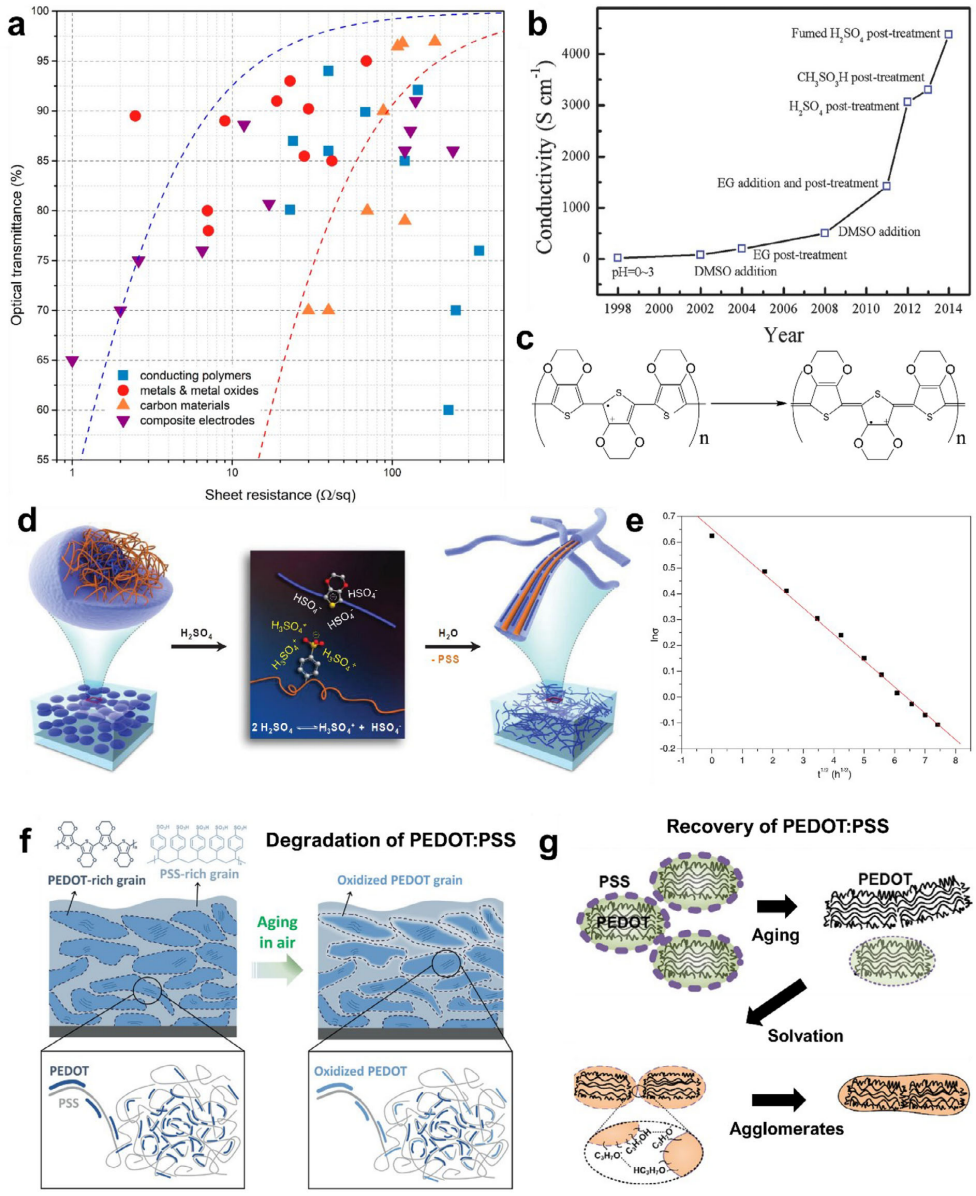
Transparent Polymers. a) Optical transparency and sheet resistance of various available TC materials. Reprinted with permission.^[^ ^10^ ^]^ Copyright 2020, American Chemical Society. b) The performance enhancement of PEDOT:PSS. The electrical conductivity of the PEDOT:PSS with different dopants and post‐deposition treatments. Reproduced with permission.^[^ ^36^ ^]^ Copyright 2015, John Wiley and Sons. c) Shows the structural modification of the PEDOT:PSS. Reproduced with permission.^[^ ^21^ ^]^ Copyright 2004, Elsevier. d) The structure rearrangement of the PEDOT:PSS with H_2_SO_4_ treatment, amorphous PEDOT:PSS grains were reformed into highly ordered crystalline nanofibrils. Reprinted with permission.^[^ ^37^ ^]^ Copyright 2014, John Wiley and Sons. e) The decrease in the conductivity of the PEDOT:PSS with time at a given temperature at ambient conditions confirms its degradation. Reproduced with permission.^[^ ^38^ ^]^ Copyright 2009, Elsevier. f) Schematic showing the illustration of the degradation of PEDOT:PSS in air.^[^ ^39^ ^]^ Reproduced with permission. Copyright 2020, John Wiley and Sons. Schematic illustration showing the recovery of PEDOT by solvation using IPA.^[^ ^40^ ^]^ Adapted with permission. Copyright 2015, Elsevier.

The primary requisite of transparent electronics is that the materials used should be able to offer both optical transparency and electrical conductivity. High charge carrier density is one of the prerequisites for good electrical conductivity. At the same time, the presence of free charge carriers increases the chances for light absorption and thereby reduces the optical transparency. As a result, achieving high conductivity and transparency in the same material for conductors and semiconductors is somewhat at odds. A specific lower limit for these properties must be defined based on the application requirements and in this regard one of the Figures of Merit (φ_*TC*_) for transparent conductors (TCs) as:^[^ ^35^ ^]^

(1) $$\varphi_{TC}=\frac{T^{x}}{R_{s}}$$

where *T* is the transmittance, *R_s_* is the sheet resistance, and *x* is the power factor. The transparency and conductivity of a film are highly dependent on its thickness (*t*) as:

(2) $$T=e^{-\alpha t}$$

(3) $$R_{s}=\frac{1}{\sigma t}$$

where σ and α are the electrical conductivity and optical absorption coefficient, respectively. Since transmittance of 90% and above is desirable for most applications, the value of x is chosen as 10, and this shifts the φ_*TC*_ maximum to a thickness, *t*_max_ = 1/10α. Since the transmittance varies with the wavelength at which it is measured, typically the value at 550 nm (middle of the visible spectrum) is used to compute the φ_*TC*_ *as* it is close to the sensitive region of the human eye.^[^ ^10^ ^]^ **Figure** **3**a shows the transparency and conductivity performances of various available TC materials.^[^ ^10^ ^]^ With extremely high transparency and minimal sheet resistance, an ideal TC material should be located in the upper left corner of the graph. The measures to develop transparent electronic devices include using i) transparent materials that can offer good transparency at a specified thickness corresponding to achieving desired electrical properties, ii) Engineered structures that can enhance the optical transparency of electronic materials, and iii) using composites of both materials. Some of the commonly used transparent electronic materials and engineering approaches in terms of transparent, conducting, and flexible properties are discussed below.

### 3.1 Transparent Materials

#### 3.1.1 Oxides

Indium Tin Oxide (ITO) is the most commonly used commercial TC due to its excellent optical transmittance in the visible spectrum, electrical conductivity, substrate adhesion, and environmental stability.^[^ ^41^ ^]^ Indium oxide (In_2_O_3_) is an *n*‐type material with a bandgap of 3.7 eV,^[^ ^42^ ^]^ which is large enough that a photon in the visible region (400–800 nm) cannot excite an electron from the valence to the conduction band; hence, it is an insulator at room temperature. To make it conduct, the approach is to increase the free carrier density by degenerate doping, which pushes the Fermi level within the conduction band. For this, Sn can be used as a dopant, substituting In in In_2_O_3_, which can act as an electron donor with an ionization level close to the conduction level. However, a high concentration of impurity dopants can reduce carrier mobility, necessitating a trade‐off to optimize the electrical properties. Pulsed laser deposited (PLD) ITO over a PET substrate exhibits a transparency of 87% and sheet resistance of ≈35 Ω sq^−1^ with a φ_*TC*_ of 7.1 mΩ^−1^.^[^ ^43^ ^]^ Indium, used in ITO, is a rare, expensive element and toxic in nature.^[^ ^44^ ^]^ Fluorine‐doped tin oxide (FTO) is an alternative and widely used TC. A transparency of 81.9% and sheet resistance of 21.8 Ω sq^−1^ have been reported for flame‐assisted spray deposited FTO over the soda‐lime glass with a φ_*TC*_ of 6.2 mΩ^−1^, which is very close to that of ITO.^[^ ^45^ ^]^ Stoichiometric point defects such as oxygen vacancies can readily ionize at room temperature in some metal oxides, particularly those with d10 cations such as ZnO, and can donate electrons for conduction.^[^ ^46^ ^]^ The electrical properties can be further improved by doping with Al. By varying the thickness, Al‐doped ZnO (AZO) can exhibit a transmittance between 86.7% to 80.1%, and the corresponding sheet resistance will vary from 230 to 85 Ω sq^−1^ with a corresponding φ_*TC*_ of 1 to 1.2 mΩ^−1^, which is very low compared to the φ_*TC*_ of ITO and FTO.^[^ ^47^ ^]^ Elements such as Ga, F, and In were also used for doping in ZnO to improve the optoelectronic properties.^[^ ^48^ ^]^

Oxides have been widely employed as transparent semiconducting materials for decades due to their high electron mobility and wide band gap.^[^ ^46^ ^]^ The conduction band minimum consists of the highly dispersive *ns*‐orbital of the metal, while the *2p*‐orbital of oxygen that is localized in nature forms the valence band maximum.^[^ ^49^ ^]^ It results in a smaller effective mass for electrons compared to holes and, thereby, high electron mobility. ZnO, IGZO, and SnO_2_ are widely employed as n‐type metal oxide materials.^[^ ^50^ ^]^ Metal oxides such as NiO, Cu_2_O, and CuMO_2_ have demonstrated p‐type behavior because of their smaller hole‐effective mass. These materials have metal cations that introduce occupied *s* or *d* orbitals near the valence band maximum, thereby enhancing the dispersion and helping to achieve low hole‐effective mass. Since the amount of oxygen significantly decides the conductivity of metal oxides, they were widely employed for sensing gases that can readily oxidize or reduce the materials.^[^ ^51^ ^]^

Because metal oxides are ceramics, most are brittle, and the high temperature pre‐ or post‐deposition requirements are incompatible with the low thermal budget of flexible substrates and thus unsuitable for flexible and wearable device applications. Furthermore, doped metal oxides necessitate highly sophisticated deposition techniques in a precisely controlled atmosphere, which are inefficient in terms of material utilization.^[^ ^52^ ^]^ In recent decades, many other materials have been studied as metal oxide substitutes for their potential as transparent electronic materials. Conducting polymers, carbon nanotubes (CNTs), graphene, metallic thin films, and one‐dimensional nanostructures have been reported as TCs, especially for flexible electronic applications.

#### 3.1.2 Polymers

Polymers with conjugated structures can transport carriers through de‐localized π bonds. The bandgap of these polymers can be tuned through chemical modifications and thus their optical properties.^[^ ^53^ ^]^ Polyethylene dioxythiophene (PEDOT) blended with a negatively charged polymer, polystyrene sulphonate (PSS), is a widely used TC polymer.^[^ ^1i,k^ ^]^ We can form TC films of PEDOT:PSS using solvent‐based deposition techniques. Pure PEDOT:PSS has a conductivity <1 Ω^−1^ cm^−1^ which is below par as a TC electrode (>10^4^ Ω^−1^ cm^−1^ for ITO^[^ ^54^ ^]^). But we can drastically improve the electrical performance by adding other organic compounds such glycerol, dimethylformamide (DMF), ethylene glycol (EG), dimethyl sulfoxide (DMSO), and tetrahydrofuran (THF) (Figure 3b).^[^ ^36^, ^55^ ^]^ Doping changes the structure of the PEDOT from a coil‐like benzoid to a more linear quinoid structure (Figure 3c), which improves intermolecular charge transport and thus carrier mobility.^[^ ^21^, ^56^ ^]^ The properties of the PEDOT films can also be improved by post‐treatment with acids and solvents.^[^ ^8a^ ^]^ When amorphous PEDOT:PSS grains were treated with concentrated H_2_SO_4_, they were observed to be reformed into highly ordered crystalline nanofibrils via a charge‐separated transition mechanism, as shown in Figure 3d.^[^ ^37^ ^]^ Polymers such as polyaniline (PANI) and polypyrrole (PPy) are also used as TC polymers.^[^ ^18a^ ^]^

A variety of π‐conjugated organic semiconductors that can offer *N*‐type, *P*‐type, and ambipolar conductivities were used.^[^ ^17d^ ^]^ The major advantages include precise tuning of optoelectronic properties through bottom‐up chemical synthesis routes, solution deposition compatibility, and lightweight and mechanical flexibility. Small‐molecule organic semiconductors such as pentacene and rubrene have been widely used in organic field‐effect transistors, especially because they can exhibit a high degree of crystallinity.^[^ ^57^ ^]^ In comparison to small molecules, polymers exhibit low crystallinity and, thereby, semiconducting performance. However, the long‐chain structure facilitates higher carrier mobility and mechanical flexibility. Polymers such as Poly(3‐hexylthiophene) (P3HT) and 2,7‐dioctyl[1]benzothieno[3,2‐b][1]benzothiophene (C8‐BTBT) are commonly used semiconductors in organic devices.^[^ ^58^ ^]^

Despite being highly flexible, polymers are not commercially popular as transparent materials because their electrical efficiency is much lower for a given thickness, their mechanical properties (such as hardness, scratch resistance, etc.) are poor, and some are highly hygroscopic, making them unstable under normal atmospheric conditions and prone to degradation. The decrease in conductivity of the PEDOT:PSS with ambient exposure is shown in Figure 3e, confirming the electrical degradation.^[^ ^59^ ^]^ When deposited on a substrate, PEDOT:PSS exhibits pancake‐like morphologies comprising PEDOT‐rich and PSS‐rich grains (Figure 3f).^[^ ^39^ ^]^ The conductivity is governed by the connectivity between PEDOT‐rich grains, where PEDOT crystallites form via π–π interactions and coexist with individual PEDOT chains stabilized by PSS. When exposed to air, water may be absorbed into the film, causing oxidation of PEDOT at the surface of the PEDOT‐rich grains. This leads to a decrease in the degree of overlap between the grains, hindering carrier transport and thereby increasing the resistance. However, recent studies also indicate that PEDOT:PSS takes several years to degrade, and the degradation by‐product could include microplastics.^[^ ^13b^ ^]^ Such observations suggest that the degradation of transparent electronics alone is insufficient, and there is also a need to analyze the degradation by‐products. The recovery of such hard‐to‐degrade materials could be interesting; the recovery of aged PEDOT:PSS was demonstrated.^[^ ^40^ ^]^ Aging of PEDOT:PSS leads to phase separation, forming hydrophobic PEDOT‐rich agglomerates as the hydrophilic PSS dissolves in water. Recovery is achieved by adding isopropyl alcohol (IPA), which preferentially solvates the PEDOT‐rich domains and redistributes them uniformly. The mechanism is shown in Figure 3g.

#### 3.1.3 2D Materials

**Figure 4:**
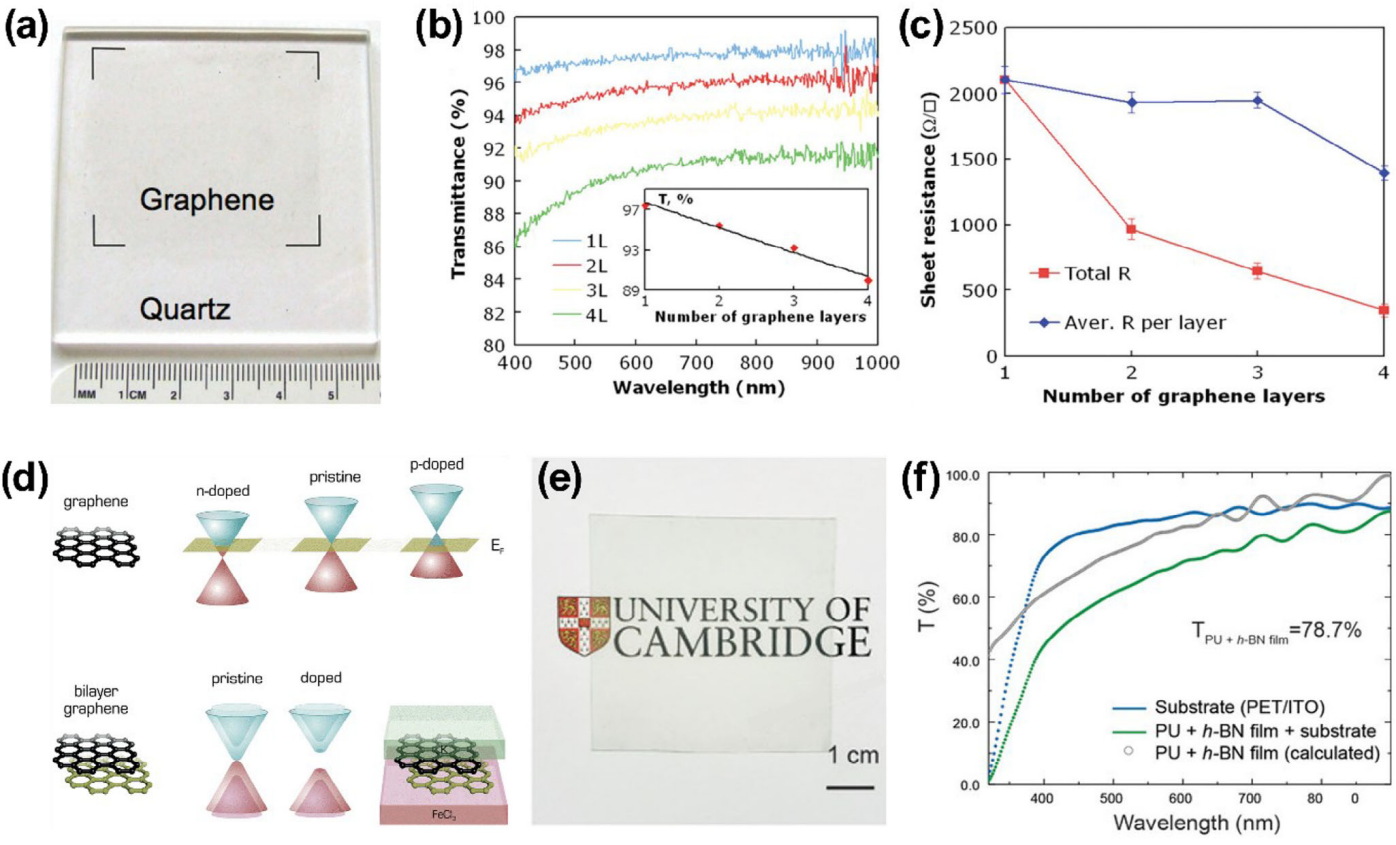
2D Materials. a) Photograph showing the graphene on a quartz substrate. The variation in b) optical transparency and c) sheet resistance with different numbers of graphene layers. Reproduced with permission.^[^ ^61^ ^]^ Copyright 2009, American Chemical Society. d) Band structure of single‐layer graphene under pristine, n‐ and p‐doped conditions and dual‐doped bilayer graphene. Reproduced with permission.^[^ ^22^ ^]^ Copyright 2016, Royal Society of Chemistry. e) Photograph of transparent h‐BN coating and f) its transmittance measured using UV–vis spectroscopy. Reproduced under the terms of the CC‐BY license.^[^ ^64^ ^]^ Copyright 2020, John Wiley and Sons.

Due to their atomic thickness, 2D materials have received wide attention for flexible transparent electronics, along with good electrical performance, optical transparency, and mechanical flexibility. One such 2D material is the carbon‐based graphene sheets (**Figure** **4**a). An sp2 bonded two‐dimensional monomer of carbon, named graphene, is widely used as a large‐area TC, because of its unique conduction properties, high mechanical strength, flexibility, and chemical resistance.^[^ ^60^ ^]^ Figures 4b,c illustrates the relationship between the number of graphene layers and their optical transparency and electrical properties, respectively. Going from a single to four layers of graphene, the sheet resistance can change from 350 kΩ sq^−1^ to 2.1 kΩ sq^−1^ while maintaining optical transparency greater than 90% (φ_*TC*_ = 0.2 mΩ^−1^).^[^ ^61^ ^]^ Large‐area transparent single‐layer graphene on flexible PVC substrate was reported with a sheet resistance of 4.71 kΩ sq^−1[^ ^2a^ ^]^ It exhibits excellent stability against mechanical bending with less than 1% resistance change after 100 bending cycles of 4 mm in bend radius (≈1.7% strain). The electronic properties of graphene can be affected by adsorbed molecules, as they can either donate (*n*‐type) or accept (*p*‐type) electrons to modulate carrier concentration(Figure 4d).^[^ ^22^ ^]^ This property of graphene can be utilized to tune the semiconducting properties of graphene or for chemical sensing applications. Even though graphene is a zero bandgap material, a band gap can be introduced by doping (dopants such as FeCl_3_, AuCl_3_, SnCl_2_, IrCl_3_, and RhCl_3_) and thereby control the type and concentration of the charge carriers.^[^ ^9a^ ^]^ For example, AuCl_3_ can strip an electron from graphene, producing *p*‐type graphene with an overabundance of holes for conduction. As a result, a significant decrease in sheet resistance was observed without much reduction in transparency.^[^ ^62^ ^]^ Another interesting approach to controlling the band gap is through dual‐doping of bi‐layer graphene, where individual layers were precisely doped using donor and acceptor atoms (Figure 4d).

Molybdenum disulfide (MoS_2_) is a layered transition metal dichalcogenide (TMD) that can offer transparent semiconducting behavior.^[^ ^63^ ^]^ A monolayer MoS_2_ can exhibit >90% optical transparency in the visible spectrum and have a direct band gap of 1.8 eV. Monolayer MoS_2_ has been widely explored to make transparent field‐effect transistors. Hexagonal boron nitride (h‐BN) is an insulator analogy of 2D materials with a wide band gap of 6 eV.^[^ ^64^ ^]^ Apart from a large bandgap, h‐BN has large resistivity, high breakdown strength, good elastic constant, and a permittivity of ≈4.9, making it a better dielectric even at atomic thickness.^[^ ^65^ ^]^ Figure 4e shows a photograph of the h‐BN insulating coating, which exhibits an average transmittance above 78%, as illustrated in Figure 4f.

Handling 2D graphene is difficult, and it is challenging to deposit and pattern over a large area. Solution‐based layer development on top of graphene might be problematic because of its high hydrophobicity, and surface modification methods like plasma treatment can harm the delicate graphene layers.

#### 3.1.4 Transient and Emerging Materials

Modern applications demand materials that not only provide fundamental transparent and conductive properties but also offer advanced functionalities, such as biodegradability and self‐healing. These emerging capabilities are increasingly important for the development of sustainable and next‐generation electronic devices, which will be discussed in the following.

##### Biodegradable and Transient Materials

**Figure 5:**
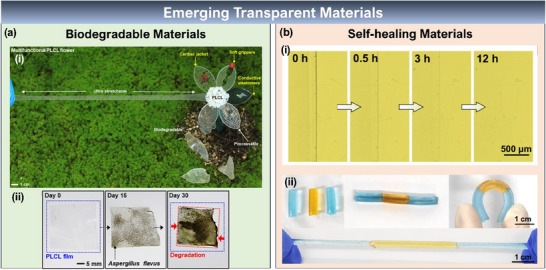
Emerging Transparent Materials. a) Biodegradable Materials. i) PLCL elastomer‐based biodegradable transparent, stretchable film demonstrating its potential applications. ii) optical images of the enzymatic degradation of the PLCL Film. Reproduced under the terms of CC‐BY license.^[^ ^66^ ^]^ Copyright 2023, Springer Nature. b) Self‐healing Materials. i) Demonstrating the self‐healing process of the PAA/betaine elastomer, a scar autonomously healed after 12 h at RH 80%. ii) Three colored individual PAA/betaine elastomer films healed together to withstand mechanical bending and stretching. Reproduced under the terms of the CC‐BY license.^[^ ^79^ ^]^ Copyright 2021, Springer Nature.

Recently, there has been a growing demand for transient electronic devices designed to decompose after fulfilling their intended purpose, enabling the development of biological implants and eco‐friendly electronics.^[^ ^66^ ^]^ However, the primary challenge is to replace plastic‐based transparent substrates such as PET and PI. Various bio‐based materials have emerged as a suitable substitute for transparent plastic substrates, which are a potential solution. **Table** **1** shows some bio‐based transparent flexible substrates and their physical properties. The substrates derived from proteins such as nanocellulose are among the cheapest bio‐degradable substrates for large‐area fabrication.^[^ ^67^ ^]^ Materials developed from starch, glucose, such as chitosan, and natural resins, like shellac, were also reported. They have a comparable performance with plastic substrates and have better thermal stability, which is desirable for manufacturing devices with improved and reliable performance, and at the end of life, they will degrade into compost in an eco‐friendly way. Hence, such bio‐substrates have a high potential to overrule plastics and need to be promoted, even commercially, as a sustainable solution for the future. Most can also be used as suitable dielectric materials, especially in pressure sensors, supercapacitors, or gate dielectrics in transistors.^[^ ^67^ ^]^ Recently, a super‐elastic biodegradable elastomer has been demonstrated using poly(l‐lactide‐co‐ε‐caprolactone) (PLCL) (shown in **Figure** **5**a).^[^ ^66^ ^]^ The material has been demonstrated to form a composite with various conductive materials, such as PEDOT:PSS, and hence, it can be used as a conductive material in addition to the insulating substrate. Conductive polymers including PEDOT:PSS, PANI, metallic NWs of Mg, Tn, and Fe, are good conductors while P3HT and metal‐oxides such as ZnO, IGZO are good semiconductors that can offer biodegradability.^[^ ^68^ ^]^ Many π‐conjugated polymer materials are available in nature, have good biocompatibility, and can serve as semiconductors and conductors, which needs further exploration.

**Table 1:** Biodegradable transparent and flexible substrate.

Material	Optical transparency	Mechanical strength	Thermal stability	Remarks
Biomass‐based polyimide^[^ ^69^ ^]^	>80%	UTS = 115 MPa E = 3.3 GPa	*T*_d_ > 400 °C *T*_g_ > 250 °C	OFET on PI exhibited stable performance after 1000 bend cycles and baking at 200 °C for 2 h
Flexible transparent wood^[^ ^70^ ^]^	>90%	UTS = 41.2 MPa	–	A TCE with 80% transmittance and 11 Ω sq^−1^ was demonstrated with Ag NW coating
Nanocellulose paper (NCP)^[^ ^67^ ^]^	>90%	UTS = 62.8 MPa; E = 3.5 GPa	Stable up to 310 °C	Disposable by flame burning. Perovskite solar cells on NCP exhibited >80% of original efficiency after 50 bending cycles
Edible Starch− Chitosan composite^[^ ^71^ ^]^	>90%	UTS = 31.6 MPa	‐	SCNT‐Graphene‐PEDOT:PSS electrode with 83% transmittance and 46 Ω sq^−1^ sheet resistance <3% resistance variation after 200 bending cycles. Degrade in 8 min in lysozyme
Isotropic paper^[^ ^72^ ^]^	>90%	–	‐	Environmentally degraded in 6 days
Starch paper^[^ ^73^ ^]^	>93%	UTS = 39.8 MPa	‐	Degrade in water after 24 h No deformations after 1000 bending at 26% bending strain
Nanopaper^[^ ^74^ ^]^	>90%	UTS = 287 MPa; E = 9 GPa	Maximum handling at 200 °C	Demonstrated flexible OLED on nanopaper

UTS‐Ultimate Tensile Strength, E‐Young's modulus.

##### Self‐Healing Materials

Self‐healing materials are another emerging material that can regain their properties and functionality after mechanical damage through self‐repairs. This embodied intelligence in the materials is inspired by the degenerative ability of biological tissues in the human body. Two approaches can achieve the self‐healing properties; i) through intrinsically designing the material to have dynamically reformable bonds and ii) through external systems, by loading healing agents into the material matrix.^[^ ^75^ ^]^ The intrinsic self‐healing materials have either highly chemically active or electrostatically attracting ends that try to rejoin once a crack is developed. The regeneration can be accelerated through external stimuli such as heat or light. In second scenario, the materials will be structurally embedded with microcapsules that are filled with adhesives. The mechanical crack will open these microcapsules, thereby releasing the glue to seal the crack simultaneously.^[^ ^76^ ^]^ Ionic conductors such as hydrogels,^[^ ^77^ ^]^ ionogels,^[^ ^78^ ^]^ and ion‐conducting elastomers^[^ ^77^, ^79^ ^]^ have been widely employed as intrinsic transparent self‐healable conductors. A 100% self‐healable ionic elastomer of polyacrylic acid/betaine has recently demonstrated ultra‐high transparency of 99.7%, 42.2 mS m^−1^ protonic conductivity, and nearly 100% elasticity (Figure 5b).^[^ ^79^ ^]^ The main disadvantage of ionic conductors is that such material will become brittle or shrink at extreme temperatures.^[^ ^77^ ^]^ The conductivity highly depends on external factors such as temperature and humidity, at the same time, the low carrier mobility is another challenge.^[^ ^80^ ^]^

### 3.2 Engineered Structures for Transparent Electronics

Most of the transparent materials examined thus far have acceptable transparency but require enhancement in electrical conductivity, especially due to limited thickness requirements. Another strategy is to consider materials with extremely high electrical conductivity and enhance their transparency by adopting various engineering approaches.

#### 3.2.1 Nanomesh and Nanostructures

**Figure 6:**
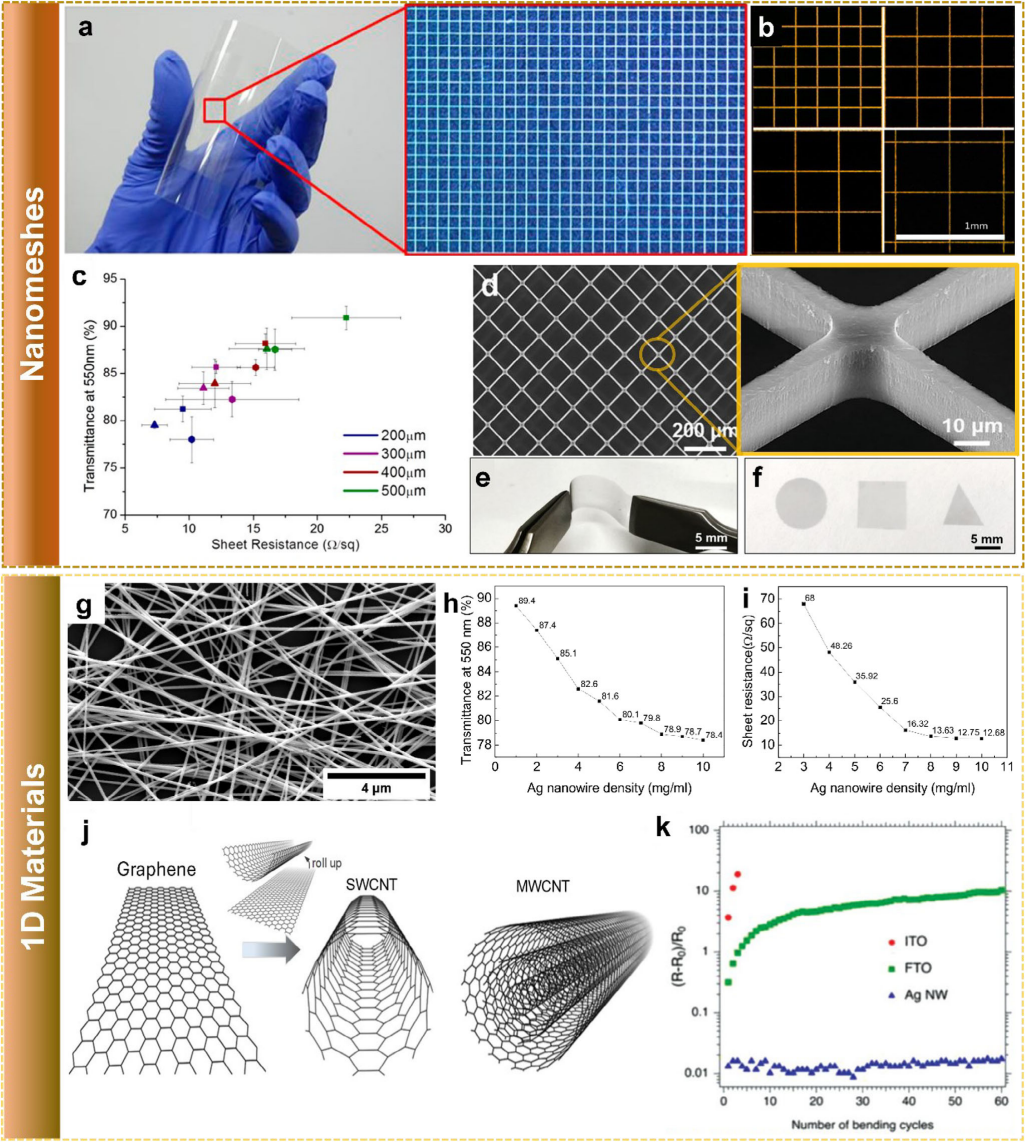
Engineered structures for transparent electronics. a) Metallic grid prepared on flexible PEN substrate for TC applications. b) Square metallic grids of 200, 300, 400, and 500 µm grid sizes, respectively. c) Transmittance at 550 nm and sheet resistance for different shapes and grid sizes. Reprinted with permission.^[^ ^24^ ^]^ Copyright 2013, American Chemical Society. d) Single crystalline Si nanomeshes with the inset showing the magnified image. e) Photograph of Si nanomeshes sample being bent with tweezers. f) Photographs of customized Si nanomeshs in various shapes. Reprinted with permission.^[^ ^11e^ ^]^ Copyright 2021, John Wiley and Sons. g) SEM image of Ag NW‐based TC film deposited on PET. The variation in the h) optical transmittance at 550 nm and i) sheet resistance as a function of the Ag NW density. Reproduced with permission.^[^ ^23^ ^]^ Copyright 2018, Elsevier. j) Rolled graphene sheet‐like structure of single‐walled (SWCNT) and multi‐walled CNTs (MWCNT). Reproduced under the terms of CC‐BY.^[^ ^87^ ^]^ Copyright 2019 MDPI. k) The resistance variation of different TC materials with mechanical bending test. The resistance of the Ag NW was observed to be unaffected by the mechanical deformation. Reprinted with permission.^[^ ^88^ ^]^ Copyright 2020, John Wiley and Sons.

Metals have a high electrical conductivity because of the large free electron density at room temperature (typically 10^22^–10^23^ electrons cm^−3^). At the same time, a continuous film of metal is opaque and reflects light in the visible range. A continuous metallic film with comparable sheet resistance to that of ITO has less than 50% optical transmittance, which is not acceptable for TC applications.^[^ ^81^ ^]^ However, if a mesh structure is used instead of continuous film, light can travel through the gaps and enhance total optical transparency. Ultra‐thin metallic films (typically < 10 nm), metallic nanowires (NWs), and nanomeshes are widely studied as TC electrodes. A 7 nm thin Au foil can be used as a TC electrode with around 80% transparency and 370 Ω sq^−1^ of sheet resistance.^[^ ^82^ ^]^ Au nanomesh has reported a better performance TC electrode with a transparency of 83% at a sheet resistance of 20 Ω sq^−1^ rather than a continuous film (**Figure** **6**a).^[^ ^11d^ ^]^ The TC performance of the nanomeshes is controlled by parameters such as grid pitch size, thickness, and width of the mesh lines. Figure 6b shows the optical photographs of a Ag nanoparticle‐based nanomesh of various grid pitch sizes, and Figure 6c shows the corresponding variation in the optoelectronic properties.^[^ ^24^ ^]^ The mesh dimensions control both conductivity and transparency, and the approach can be extended to any functional material to make it optically transparent. In a similar direction, a 96% transparent single‐crystalline Si nanomesh framework was also demonstrated (Figure 6d), confirming the adaptability of the approach to various electronic materials including semiconductors.^[^ ^11e^ ^]^ Nanomesh fabrication and patterning are difficult processes to obtain uniformly over a large area.

A simple direct coating of NWs can form a mesh‐like nano network and can function as transparent functional materials, with the performance depending on the properties of the individual NWs and the network morphology. Figure 6e shows the SEM image of Ag NW‐based TC film.^[^ ^11b^ ^]^ With a decrease in the NW length and diameter, an increase in resistance is expected since more junctions are needed per unit length. Similarly, when the diameter of the individual wires decreases, the resistance of the wires increases owing to surface scattering, and it will be highly significant when the NW diameter is closer to the mean free path of the electrons of the bulk metal.^[^ ^83^ ^]^ Typically, Ag and Cu have a mean free path of nearly 50 and 40 nm, respectively.^[^ ^84^ ^]^ High aspect ratio (length/diameter) NWs are highly desirable for TC applications.^[^ ^85^ ^]^ The areal density of the wires is the other parameter (Figure 6f,g). With more wires, multiple paths are available for the carriers to flow and lower the resistance. At the same time, the optical transparency will be reduced, and hence, a balance is needed between both parameters. The conductivity of individual NWs depends on the material used. Ag and Cu are studied widely, and among them, Ag is preferable because of its good electrical properties and resistance to oxidation. The network resistance is dominated by the junction resistance between the wires, which depends on how the nanowires are synthesized and how they are purified and deposited.^[^ ^86^ ^]^ Methods such as thermal annealing, hot pressing, electrical annealing, optical sintering, and encapsulation have been reported as techniques to fuse the NWs and thereby reduce the junction resistance.

Another widely studied carbon‐based material is the 1D carbon nanotubes (CNTs), with a rolled‐up graphene sheet structure.^[^ ^89^ ^]^ Based on the number of graphene sheet layer rolls, CNTs can be single‐wall (SWCNT) or multi‐wall (MWCNT) (Figure 6h) in structure.^[^ ^87^ ^]^ The benefit of CNTs is that we can efficiently produce CNT films utilizing solution‐based techniques, chemically functionalize their conductivity, and also tune them as semiconducting material. CNT‐based TC films typically have a sheet resistance of 400 Ω sq^−1^ with >90% transmittance (φ_*TC*_ = 0.9 mΩ^−1^), the contact resistance between the tubes dominates the electrical characteristics.^[^ ^81^ ^]^ There have been several reports of surface modification and functional treatment techniques that might further enhance electrical performance, but more enhancements need to emerge before they can be used as reliable TC material.^[^ ^10^ ^]^

One of the main advantages of the 1D materials is their mechanical properties. Because of their high aspect ratios and wire‐like structure, they are very ductile, and mechanical deformations such as compression, tension, and torsion do not affect the electrical characteristics. In comparison to ITO and FTO, the resistance of Ag NWs was observed to remain constant with multiple bending cycles, as shown in Figure 6i.^[^ ^86^, ^88^ ^]^ Brittle metal‐oxides, such as ZnO, can also made flexible in the form of NWs and have been reported to fabricate flexible devices.^[^ ^90^ ^]^ It has also been demonstrated that NW‐based films can be used as stretchable electronics, too.^[^ ^91^ ^]^

### 3.3 Combining Materials and Engineered Structures

**Figure 7:**
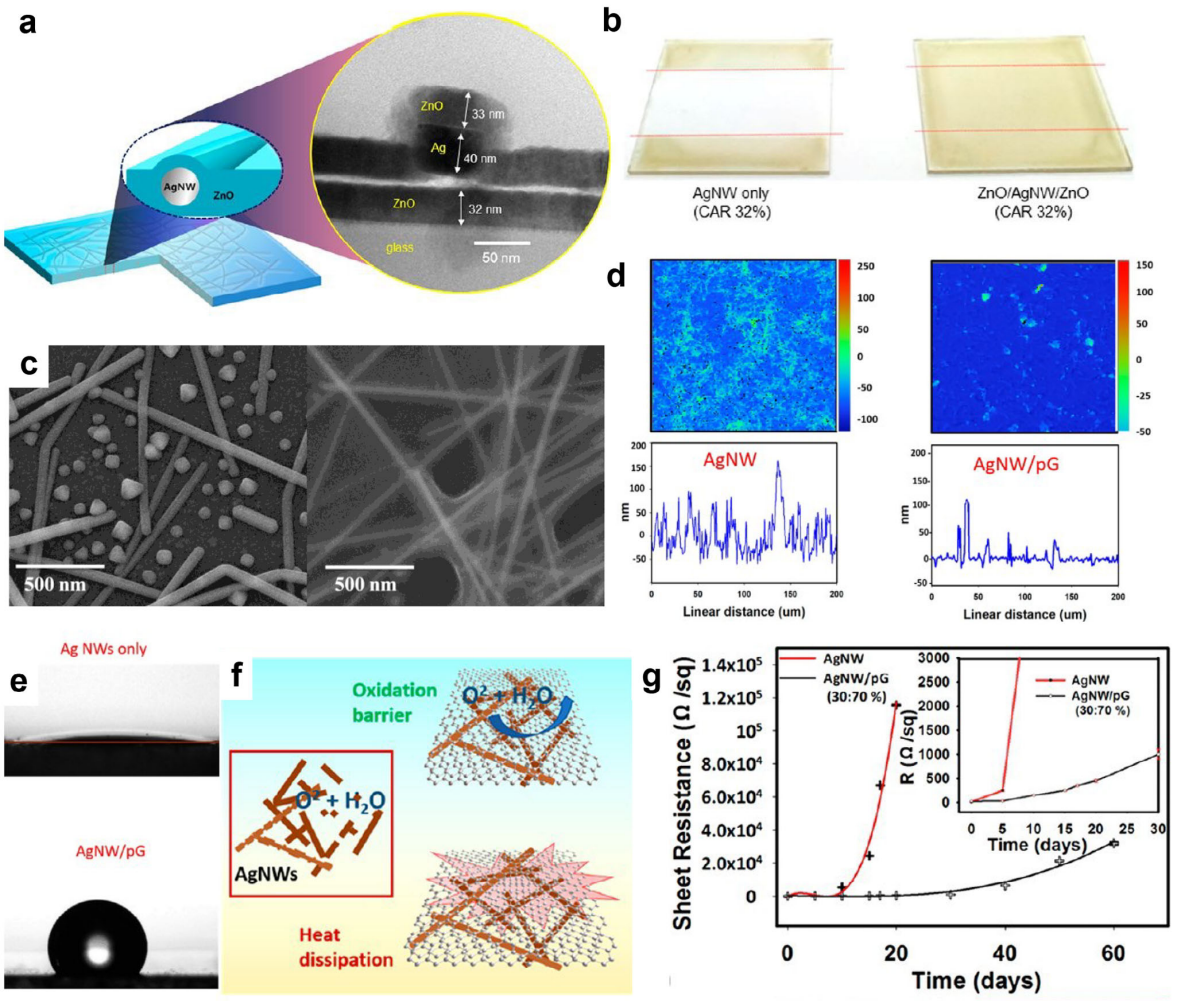
Composite materials for transparent electronics. a) Schematic of the ZnO/Ag NW/ZnO composite electrode and the cross‐sectional HRTEM image. b) The result of the scotch tape peel‐off adhesion test performed on bare Ag NW and ZnO/Ag NW/ZnO composite deposited on the glass substrate. The film looks completely detached in the case of bare Ag NWs, while looking intact for the composite. Reprinted with permission.^[^ ^92^ ^]^ Copyright 2013, American Chemical Society. c) SEM image of the bare Ag NW film annealed at 250 °C looks dissociated, while Ag NW‐ZnO composite film looks stable at 300 °C. Reprinted with permission.^[^ ^7^ ^]^ Copyright 2016, American Chemical Society. d) Comparative topography and surface profile of the Ag NW and Ag NW/graphene composite films confirming reduced surface roughness. e) Water contact angle of hydrophilic Ag NW and superhydrophobic Ag NW/graphene composite electrodes. f) The schematic representation of the graphene acting as an oxidation barrier and uniform heat distribution helps to improve the reliability of the Ag NWs. g) The improvement of the stability of the Ag NWs in ambient conditions after the introduction of the graphene. Reprinted with permission.^[^ ^94^ ^]^ Copyright 2018, American Chemical Society.

Being ceramic, commercially popular oxides such as ITO are not suitable for flexible device applications. Even though cost‐effective, polymeric TCs, including PEDOT:PSS, are hygroscopic and unreliable for long‐term atmospheric exposure. Graphene has excellent chemical resistance and mechanical stability but is very difficult to handle and pattern. Recently popular metallic NWs have high surface roughness, junction resistance, poor surface adhesion, and are easily oxidized with environmental exposure. Clearly, a single material cannot satisfy all the needs for flexible transparent electronics, and an innovative solution needs to emerge. Combining multiple materials as a composite TC film is an effective strategy and helps to achieve additional functionalities. For example, an improvement in the electrical and mechanical properties of the Ag NWs was demonstrated after binding using sputtered ZnO (as shown in **Figure** **7**a).^[^ ^7^, ^92^ ^]^ It was reported that the film roughness was reduced by planarizing the surface and improving the surface adhesion of the NWs. Adhesion of the film was tested using a 3 M scotch tape and observed to be intact for the composite, confirming its strong adhesion, as shown in Figure 7b.^[^ ^92^ ^]^ The thermal stability was also observed to be improved, the bare NWs were dissociating into particles at 250 °C while looks intact for the Ag NW‐ZnO composite even at 300 °C (Figure 7c).^[^ ^7^ ^]^ Similar functional improvements were reported for Ag NW‐PEDOT:PSS nanocomposites.^[^ ^93^ ^]^ Ag NW‐graphene TC composites were also reported, where the graphene will help to improve the surface roughness (Figure 7d) and also to convert the hydrophilic surface to superhydrophobic (Figure 7e), which helps to enhance the water‐repelling properties.^[^ ^94^ ^]^ Graphene has an excellent oxidation barrier and thermal conductivity. Hence, the graphene will protect the underlying metals from corrosion and oxidation and improve the thermal stability by reducing the localized heating by better thermal distribution, the mechanism is demonstrated in Figure 7f. Because of these properties, Ag NW‐graphene composite have been reported to be highly stable under ambient and harsh environmental conditions, a comparative improvement in the stability of the composite film at ambient is shown in Figure 7g.

**Table** 2 summarizes the various TC materials based on their optoelectronic properties. In general, these materials can be divided into two categories: inherently transparent materials (such as oxides, polymers, and graphene sheets) and structurally transparent materials (such as CNTs, NWs, and metallic meshes) and their composites. Table 2 further classifies them based on fabrication methods—the first set are the ones processed using conventional micro/nanotechnology approaches such as lithography, sputtering, etc., and the second category of materials that are can be processed using emerging resource‐efficient approaches such as printed electronics. Among them, the highest φ_*TC*_ was reported for transparent metallic meshes. Factors, including optimizing the mesh dimensions and the long‐term reliability of the technology, are still under development. The grid size needs to be reduced further, below 100 µm, and line width to the sub‐micron regime for better carrier collection/supply. An idle solution will be the composite films in the future.

**Table 2:** Summary of the optoelectronic properties of the various TC materials.

	Reference	Material	*T* [%]	*R*_sh_ [ohm sq^−1^]	φ_*TC*_ [mΩ^−1^]	Thickness [nm]	Flexibility	Fabrication
Fabrication using conventional methods								
Oxides	[98]	ITO	83	3.1	50.1	600	No	DC sputtered on glass
	[99]	FTO (NaOH assisted doping)	80.8	5.3	22.4	≈700	No	Spray pyrolysis on glass
	[100]	AZO	84.2	9	19.9	≈300	No	Sputtered on glass
	[101]	V‐doped ZnO	85	220	0.89	100–150	No	PLD on Al_2_O_3_
Polymers	[102]	PEDOT:PSS (DMSO doped)	82	46	2.9	N/A	Yes (Stretchable)	Spin coated on pre‐strained PDMS
	[103]	PEDOT:PSS (DMSO doped)	92.3	100	4.5	80	No	Spin coated on glass
	[104]	n‐PBDF	80	45	2.4	17–94	No	Spin coating followed by lithographic patterning
2D Materials	[105]	Graphene	80	280	0.38	N/A	Yes	CVD growth followed by Transfer
	[106]	Graphene	90	40	8.72	N/A	Yes	CVD
Nanostructures	[107]	Au mesh	95	8.03	74.6	200	Yes	Lithographically patterned and electrochemical peel off to PDMS
	[108]	Ag/Ni mesh	88.6	2.1	141.9	N/A	Yes	Blading Ag followed by electroplate Ni on PET
	[109]	SWCNT	88	82	3.4	7000	Yes	Embedded in PI
Composites	[110]	AZO/Ag NW/AZO/ZnO	93.4	11.3	44.71	150	No	Spin coated on glass
	[111]	Ag NW/Graphene	89	13.7	22.7	50	Yes	Spray coating on PEN
	[92]	ZnO/Ag NW/ZnO	92	8	6.5	N/A	Yes	Sputter (ZnO) and spin (Ag NW) coated on polymer
	[112]	Ag NW/IZO/PEDOT:PSS	86	5.9	37.5	N/A	Yes	Spin coating (Ag NW, PEDOT) and sputtering (IZO) on PEN
	[1i]	Ag NW+PEDOT:PSS	77	7	10.5	1940	Yes	Electrospinning
	[113]	Graphene+CNT	86	240	0.92	N/A	Yes	Spin coated on PET
	[114]	Ag NW/PEDOT:PSS/Graphene	88.3	30	9.6	50–100	No	Coating and transfer (Graphene) on glass substrate
Fabrication using resource efficient methods								
Polymer	[115]	PEDOT:PSS (H_2_SO_4_ treated)	90	46	7.6	N/A	Yes	Transfer printing to PEN
	[116]	PEDOT:PSS (EG doped)	75	58	0.97	3900	Yes (Stretchable)	Inkjet printing
2D Materials	[117]	Graphene	90	30	11.6	N/A	Yes	Roll to roll printing on PET
	[61]	Graphene	90	350	0.99	N/A	Yes	Transfer to PMMA
	[118]	Graphene (AuCl_3_ doped)	87	150	1.7	N/A	Yes	Transferred to PET
Nanostructures	[11b]	Ag NW	94	30	18.3	N/A	Yes	Printed on PET
	[119]	Ag NW	96	38	17.5	N/A	No	Meniscus‐Dragging Deposition on glass
	[120]	Cu NW	94	56.3	N/A	N/A	Yes	Transfer to PMMA
Composites	[93]	Ag NW+PEDOT:PSS	86	23.8	9.7	94	Yes	Printed on PET
	[121]	Ag NW+PEDOT:PSS	88.6	19.7	15.2	N/A	Yes	Brush painted on PU
	[122]	Ag NW+ZnO	93	28.7	16.9	N/A	Yes	Printed on PET
	[123]	Ag grid/Ag NW	87.5	16.5	15.9	N/A	Yes	Printed (Ag NP grid) and spin coated (Ag NW) on PET followed by NaCl treatment
	[124]	G/CNT/PEDOT:PSS	80	43.2	2.5	N/A	Yes	Transfer to Starch/Chi‐ tosan/Poly vinyl alcohol substrate

N/A, Data not available

An idle composite TC should have graphene‐like properties at the surface, with excellent chemical and oxidation resistance, uniformly distributed carrier collection/supply capability like an intrinsically transparent material at sub‐micron dimensions and assisted by structurally transparent metallic meshes to transport the carriers over a large area. However, composites are not idle for semiconducting materials due to the presence of multiple grain boundaries or material junctions, that can affect the electron mobility. In addition to the conductive materials, PDMS, PET, PI, and PEN are the popular flexible transparent substrate materials, and the thermal stability and the surface morphology of the substrates are critical parameters during fabrication. Parylene‐C,^[^ ^95^ ^]^ and nanocomposites of metal oxides such as ZnO^[^ ^96^ ^]^ or TiO^[^ ^97^ ^]^ with epoxies such as PDMS were widely reported as transparent and flexible protective coatings.^[^ ^12^ ^]^

## 4 Manufacturing Techniques for Transparent Electronics

The development of flexible transparent electronics is highly dependent on the utilization of deposition and processing techniques.^[^ ^48^ ^]^ Traditional microfabrication processes, including wet/vapour‐based techniques, have been widely employed due to their ability to produce high‐quality films with excellent optoelectronic properties. For instance, TC oxides such as ITO is the commonly employed transparent conductive film, which is normally fabricated either using chemical vapour deposition (CVD) or sputtering technique. Vacuum‐deposited ITO films typically exhibit excellent performance with over 90% transmittance and 10 Ω sq^−1^ sheet resistance. However, when deposited on flexible polyethylene terephthalate (PET) substrates, the performance decreases due to lower post‐annealing temperatures, resulting in a transmittance of 87% and a sheet resistance of 35 Ω sq^−1^ Inkjet‐printed ITO films show even lower performance with 85% transmittance and 520 Ω sq^−1^ sheet resistance. This demonstrates the significant impact of deposition techniques on film performance. Meanwhile, the limited reserves of indium, CVD and sputtering techniques can lead to increased cost as well as material waste, which is undesirable for cost‐effective/large fabrication purposes.^[^ ^125^ ^]^Therefore, seeking alternative materials that can replace ITO with the development of solution phase coating and printing processes is highly needed for the realization of flexible/cost‐effective, transparent large‐area electronics. As an alternative, metallic NW networks, such as silver nanowires (Ag NWs), have shown great promise. Ag NWs offer high flexibility, and ease of manufacturing through solution‐based techniques such as inkjet, and screen printing and have shown comparable performance to ITO. Randomly oriented Ag NWs networks can achieve up to 92.9% transparency at 20 Ω sq^−1^ sheet resistance, with even higher transparency (96.7%) when partially aligned. Compared to ITO, aligned Ag NW networks are more efficient in supplying and collecting charge carriers in polymer LEDs and organic solar cells, resulting in a 40% increase in maximum power efficiency and improved short circuit current. Transparent conductors realized based on the solution process show merit, including cost‐effectiveness, resource efficiency, and ease of scalable fabrication. In general, solution‐based processes, such as spin coating,^[^ ^126^ ^]^ drop casting, dip coating, and spray coating,^[^ ^127^ ^]^ are commonly used for depositing materials such as conducting polymers, NWs, and CNTs; on the other hand, graphene and ITO are typically grown using CVD.^[^ ^9a^ ^]^ While these conventional techniques, including deposition with chemical vapor‐based processes and solution‐based coating techniques, are effective in realizing high‐quality TC films with comparable optical and electrical properties with ITO.^[^ ^18b^ ^]^ Nevertheless, these solution/vapour‐based methods may represent significant challenges when scaled up for large‐area applications. This result is due to the requirements for additional lithographic steps for patterning, vacuums for vapor deposition, and wet etching using basic/acidic solutions. Furthermore, these methods are resource‐intensive, generating considerable material waste and toxic chemicals due to their reliance on microfabrication processing such as lithography and etching. Nevertheless, these wet and dry depositions are employed for research purposes and laboratory demonstration, and it's still challenging to scale up. On the other hand, large‐scale fabrication processes with commercialized potential are highly demanded. In contrast, printing technologies with solution‐processable TC materials offer a promising solution for the fabrication of large‐area electronics. In general, printing techniques facilitate a selective additive manufacturing route, with minimal material wastage and process complexity and, thereby, cost‐effectiveness.^[^ ^128^ ^]^ In addition, it is compatible with a wide range of flexible substrates because of its low thermal budget and chemical‐free etching/patterning. Printing of large areas can be efficiently enabled by various conventional printing techniques such as inkjet printing, screen printing, and stamp‐based transfer printing. In addition, other emerging printing technologies such as roll‐to‐roll compatible transfer printing techniques, including direct roll transfer, printing contact printing, and dielectrophoresis, have been explored to meet the demand for large‐scale fabrication. It has shown promising potential for precisely aligning NWs over large areas and enhancing device performance. In general, printing can meet high throughput requirements with low material consumption, which brings cost‐effectiveness and sustainability. Based on these features, research interest in the field of printed electronics has increased from both industry and academia. Several printing technologies have emerged over the last few years; some of them are introduced in this section and summarized in **Table** 3.

**Table 3:** Summary of printing and vapor deposition techniques for the fabrication of large‐area TC devices.

Technology	Contact	Dry/Wet	Viscosity	Area	Patternability	TC Materials	Material wastage	Refs.
Vapour deposition	Non‐contact	Dry	NA	Small	Complex lithography assistance	Oxides, graphene	High	[9, 18]
Inkjet printing	Non‐contact	Wet	2 to 20 mPa.s	Medium	Direct	Low aspect ratio nanoparticles	Very low	[129]
Aerosol‐jet printing	Non‐contact	Wet	1 to 1000 mPa.s	Medium	Direct	Low aspect ratio nanoparticles	Medium	[130]
Electrohydrodynamic printing	Non‐contact	Wet	1 to 10000 mPa. s	Medium	Direct	Low aspect ratio nanoparticles	Very low	[131]
Screen printing	Contact	Wet	10^2^ to 10^5^ mPa.s	Large	Require hard mask	Metallic mesh	High	[132]
Roll to roll	Contact	Dry/wet	NA	Large	Direct	Oxides, NPs, NWs, Carbon‐based, Polymers	Medium	[133]
Transfer printing	Contact	Dry	NA	Large	Using patterned stamp	Graphene, NWs	Medium	[61, 134]
Contact printing of NWs	Contact	Dry	NA	Large	By surface functionalization	Vertically grown NWs	Low	[27, 135]
DEP	Non‐contact	Wet	Low viscosity (depends on the medium used for DEP)	Large (depends on electrode design and setup)	By patterning the electrodes to control the electric field	1D Materials	Low (as it precisely positions materials)	[136]
Langmuir–Blodgett assembly	Non‐contact	Wet	Low viscosity	Large (scalable to large substrates)	Assistance from surface functionalization/lithography	1D Materials	Low (efficient use of materials in the monolayer formation)	[137]
Capillary printing	Contact	Wet	High (required for controlled capillary action)	Limited to moderate (depends on capillary and substrate size)	Direct	1D Materials	Moderate, (some wastage occurs during the process but can be minimized with precise control)	[138]

### 4.1 Conventional Printing Techniques

#### 4.1.1 Inkjet Printing

**Figure 8:**
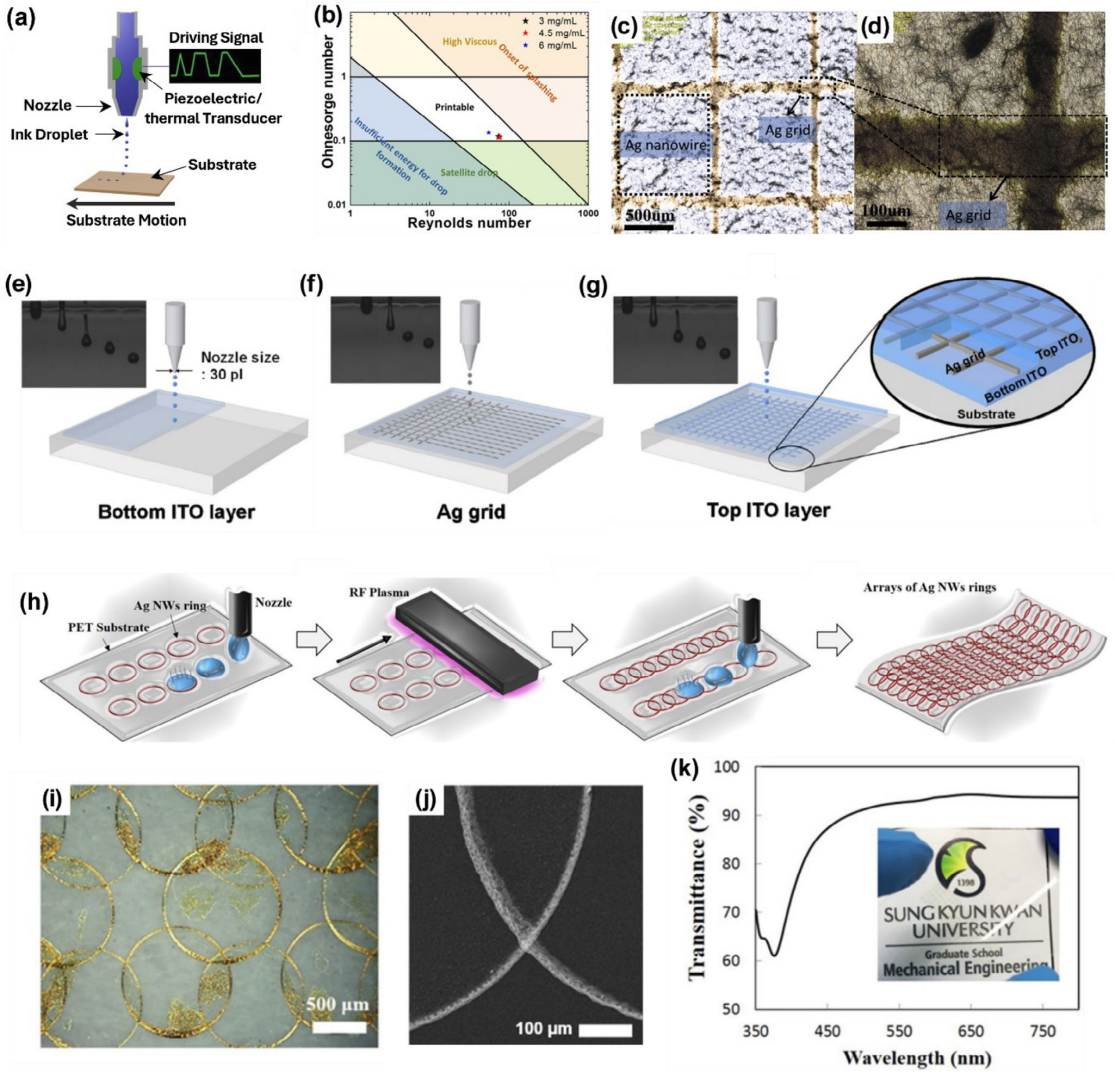
Inkjet Printing. a) Schematic illustration of inkjet printing technique. b) printable rheological region of inks defined by Ohnesorge and Reynolds numbers. Reprint with permission.^[^ ^93^ ^]^ Copyright 2020, American Chemical Society. The SEM micrograph of Ag NW/Ag grid TC electrode fabricated by inkjet printing at c) lower and d) higher magnifications. Reproduced with permission.^[^ ^129c^ ^]^ Copyright 2017, Elsevier. e–g) Schematic diagram of the fabrication of ITO/Ag grid/ITO hybrid TC electrode, with the inset showing the jetting of the ink. Reproduced with permission.^[^ ^129d^ ^]^ Copyright 2011, Elsevier. h) Schematic of fabricating the Ag NW ring array by inkjet printing, utilizing the coffee‐ring effect. i) Optical microscopy, j) SEM, and j) transmittance of the TC electrode of Ag NW ring array. Reproduced with permission.^[^ ^129f^ ^]^ Copyright 2017, IOP publishing.

Inkjet printing is a promising candidate technique for the large‐area fabrication of transparent electronics. It has the capability to print a wide range of TC inks such as polymers, carbon‐based materials, metallic NWs, etc. on various types of substrates, which therefore serves as the most used technique to produce micro‐scale patterns. Inkjet printing enables the direct printing/writing of micropatterns using nanomaterials as inks with no template with high resolution without needing lithographic assistance or hard masks for patterning.^[^ ^139^ ^]^ This method facilitates high‐resolution 2D patterning with minimal material wastage through the use of an inkjet nozzle of micrometer size in a non‐contact manner.^[^ ^140^ ^]^ As illustrated in **Figure** **8**a, Inkjet printing can be categorized into continuous inkjet or drop‐on‐demand (DoD) printing. In continuous inkjet printing, the droplets are generated continuously, whereas, in DoD printing, droplets are selectively deposited by activating the print head using a piezoelectric, thermal, or acoustic impulse as needed. For inkjet printing, the material must be in a liquid phase or uniformly dispersed or dissolved in a liquid solvent. The dispersed particle size, viscosity, surface tension, and density must be optimized to avoid print nozzle clogging of the print nozzle. Typically, the ink used in inkjet printing is typically of low viscosity, and normally the maximum dispersed particle dimensions must not exceed 50 times the print nozzle diameter.^[^ ^129a^ ^]^ Consequently, inkjet printing of high aspect ratio materials, such as NWs and CNTs, is highly challenging and often requires sonication to reduce the length while preparing the inks to avoid nozzle clogging.^[^ ^11^, ^129^, ^141^ ^]^ For example, the Inkjet printing of 2.2 µm long Ag NWs dispersed in a bi‐solvent mixture of IPA, and polyethylene glycol has been reported.^[^ ^129a^ ^]^ Newtonian dispersion with viscosity range of 2—20 mPa s and surface tension between 35 and 70 mg mL^−1^ surface tension are ideal for printing. A printable rheological region is defined (Figure 8b) by two dimensionless quantities; Ohnesorge and Reynolds numbers, which are functions of viscosity, surface tension, density, and nozzle diameter. These parameters ensure proper extrusion of the ink, droplet formation, and pattern formation on the substrate without spreading.^[^ ^11^, ^93^, ^142^ ^]^ Printable inks can be formulated adjusting the dispersant, surfactant, solvents and binding agents.^[^ ^143^ ^]^

A printable TC ink obtained dispersing PEDOT:PSS in glycerol and DI is another example.^[^ ^129b^ ^]^ The high viscosity of glycerol helped tune the dispersion's viscosity and functioned as a dopant to the PEDOT, enhancing conductivity by up to 300 times. Ye et al. demonstrated a hybrid flexible TC electrode by inkjet‐printing of Ag grid on top of spin‐coated Ag NWs (Figure 8c,d), achieving a sheet resistance of 22.5 Ω sq^−1^ and transmittance of 87.5%.^[^ ^129c^ ^]^ It has been shown the printed grids significantly improved the electrical conductivity of the electrode. Similarly, incorporating Ag grid into an ITO matrix reduced the sheet resistance from 6.5 to 0.54 Ω sq^−1^.^[^ ^129d^ ^]^ The ITO ink was prepared by dissolving indium chloride tetrahydrate, and tin chloride dehydrates in ethanol. A 1000 nm thick ITO film was printed on glass (Figure 8e), followed by a 200 nm thick Ag grid, patterned on top (Figure 8f), and finally covered with another inkjet printed ITO layer (Figure 8g). This structure demonstrated the potential of inkjet printing in developing transparent electrodes with enhanced performance. In another study, Inkjet printing of graphene dispersed in terpineol achieved 80% transmittance and 30 kΩ sq^−1^ sheet resistance.^[^ ^129e^ ^]^ Adding a small amount of ethyl cellulose prevented the agglomeration of graphene flakes and mitigated the coffee‐ring effect, which is well known undesired phenomenon where particles accumulate at the edge of a drying droplet, leading to non‐uniform films. The coffee‐ring effect has been utilized to fabricate TC electrodes by printing an array of Ag NW rings on PET substrate, as shown in Figure 8h.^[^ ^129f^ ^]^ The optical and SEM images of the Ag NW ring array are demonstrated in Figure 8i,j. The effectiveness of this technique in producing high‐performance transparent electrodes has been achieved by reporting transmittance of 91% (Figure 8k) and 49.6 Ω sq^−1^ sheet resistance. Another interesting example is the demonstration that, due to the coffee‐ring effect, inkjet printing of electrically continuous micron‐wide lines often results in the formation of twin parallel lines instead of a single uniform one. These twin‐line formations can be organized into rectilinear metallic grids, which are promising candidates for use as transparent conducting grids.^[^ ^144^ ^]^

#### 4.1.2 Aerosol‐Jet Printing

**Figure 9:**
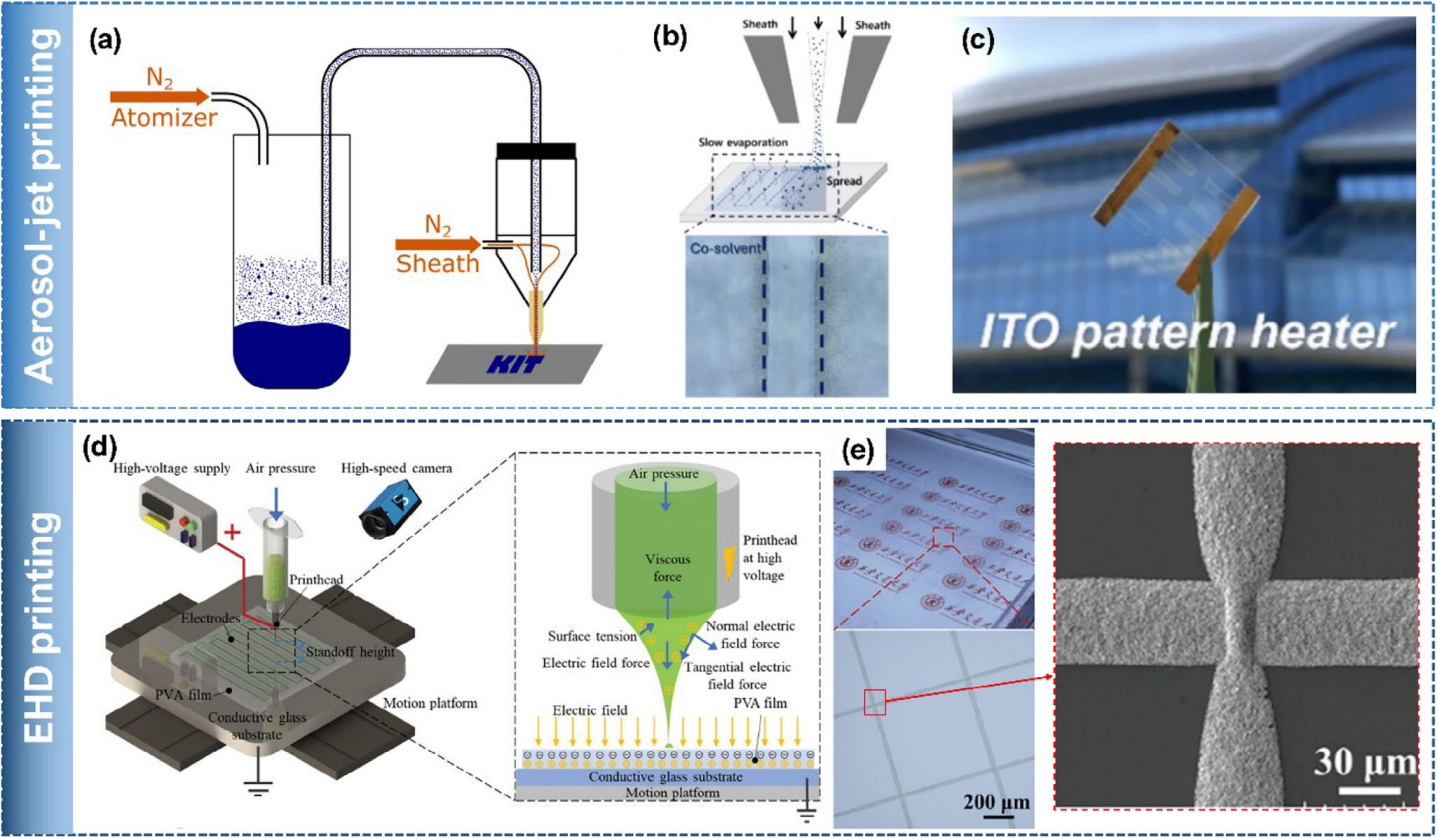
Aerosol and Electrohydrodynamic jet printing. a) The working principle of aerosol‐jet printing. Reproduced under the terms of CC‐BY.^[^ ^145^ ^]^ Copyright 2023, IOP publishing. b) Schematic and optical microscopy images showing the aerosol‐jet ITO electrodes c) Photograph thin‐film heater patterns fabricated by AJP of ITO nanoparticles. Reproduced with permission.^[^ ^130a^ ^]^ Copyright 2024, Elsevier. d) Schematic demonstrating the working mechanism of EHD jet printing. e) EHD printed Ag nanoparticle‐based meshes as TCEs. Reproduced with permission.^[^ ^131b^ ^]^ Copyright 2025, John Wiley and Sons.

Aerosol‐jet printing (AJP) is a non‐contact printing technology capable of achieving resolutions as fine as below 10 µm.^[^ ^145^ ^]^ The ink used must be a dispersion with a viscosity between 1 and 1000 mPa s. During the process, the ink is atomized into tiny aerosol droplets, transported by carrier gases such as N_2_, and directed toward the printing nozzle. At the nozzle, a sheath gas (sheath flow) surrounds the aerosol stream, focusing it and simultaneously protecting the nozzle from droplet accumulation. The print nozzle and substrate bed can be moved to print lines or complex patterns. Mechanical shutters were employed if the aerosol needed to be stopped, which leaves the technique material‐consuming in comparison to inkjet printing. The working mechanism is shown in **Figure** **9**a. Due to the large standoff distance, typically of  >3 mm, above the target substrate and due to the jetting behavior, AJP facilitates conformal printing over non‐linear surfaces of complex or multidirectional curvatures and having high surface roughness or other conditions.^[^ ^146^ ^]^

Direct patterned AJP was demonstrated with ITO nanoparticles, dispersed in a blend of methanol and ethylene glycol to improve the surface uniformity and roughness (Figure 9b).^[^ ^130a^ ^]^ The AJP‐based TCE exhibited 90.6% transmittance and 23.2 Ω sq^−1^ sheet resistance. Finally, the technique was used to realize a transparent heater that was directly patterned by AJP (Figure 9c). Similar to inkjet printing, twin‐line formation has also been observed in AJP, presenting an interesting phenomenon that can be leveraged to fabricate narrow, parallel lines for TC electrode applications.^[^ ^147^ ^]^ AJP of Ag NWs dispersed in water was used to fabricate transparent electrodes with a line width of 52 µm and a sheet resistance of 58 Ω sq^−1[^ ^130b^ ^]^ Similarly, transparent and flexible conductive electrodes were fabricated using Ag NW‐based aerosol‐jet printed conductive grids on PEN substrate. By optimizing parameters such as stand‐off height, stage speed, atomization, and sheath gas flow rates, grid lines of 150 µm width and 75 µm spacing were successfully printed. The final film showed 78% transmittance and 31 Ω sq^−1^ sheet resistance.^[^ ^130c^ ^]^ However, the conformal printing capability of AJP has not yet been explored for out‐of‐plane transparent electronic technologies, presenting a promising direction for future research (Table 3).

#### 4.1.3 Electrohydrodynamic Jet Printing

Electrohydrodynamic jet (EHD) printing is a promising non‐contact technique that has gained attention for its ability to achieve high‐resolution patterning.^[^ ^148^ ^]^ In inkjet printing, it is difficult to eject liquids through very small nozzles using thermal or piezoelectric methods. In contrast, EHD printing applies a strong electric field between the nozzle (as small as ≈100 nm) and the substrate, which pulls the ink out rather than pushing it, enabling much finer control and smaller droplet sizes.^[^ ^149^ ^]^ EHD printing achieves high resolution through a combination of key mechanisms. First, it utilizes nozzles with much smaller inner diameters than those used in conventional inkjet printing. Second, electrohydrodynamic forces enable the formation of droplets that are significantly smaller than the nozzle itself. Third, the applied electric field focuses these droplets, allowing for precise deposition with minimal lateral spreading.^[^ ^149^ ^]^ Figure 9d highlights the working mechanism of EHD printing.

EHD printing has been actively explored for the fabrication of TC electrodes, particularly for printing metallic grid structures. One notable example involved the fabrication of an invisible electrode using EHD‐printed Ag nanoparticle‐based mesh structures.^[^ ^131a^ ^]^ Ag grid lines with widths below 10 µm were printed, and the line spacing (pitch) was optimized to balance optical transparency and electrical conductivity. A pitch of 150 µm resulted in 81.75% transparency and a low sheet resistance of 4.87 Ω sq^−1^ In another study, Ag grid lines with widths of 50 µm were EHD printed on water‐soluble PVA substrates, then transferred onto non‐planar surfaces using a hydroprinting process to realize conformable TCEs (Figure 9e).^[^ ^131b^ ^]^ The resulting electrodes demonstrated excellent performance, with a sheet resistance of 3.8 Ω sq^−1^, 92.8% transparency, and a figure of merit of 1304. These results highlight the potential of EHD printing as a promising approach for developing conformable electronics and sensors.

#### 4.1.4 Screen Printing

**Figure 10:**
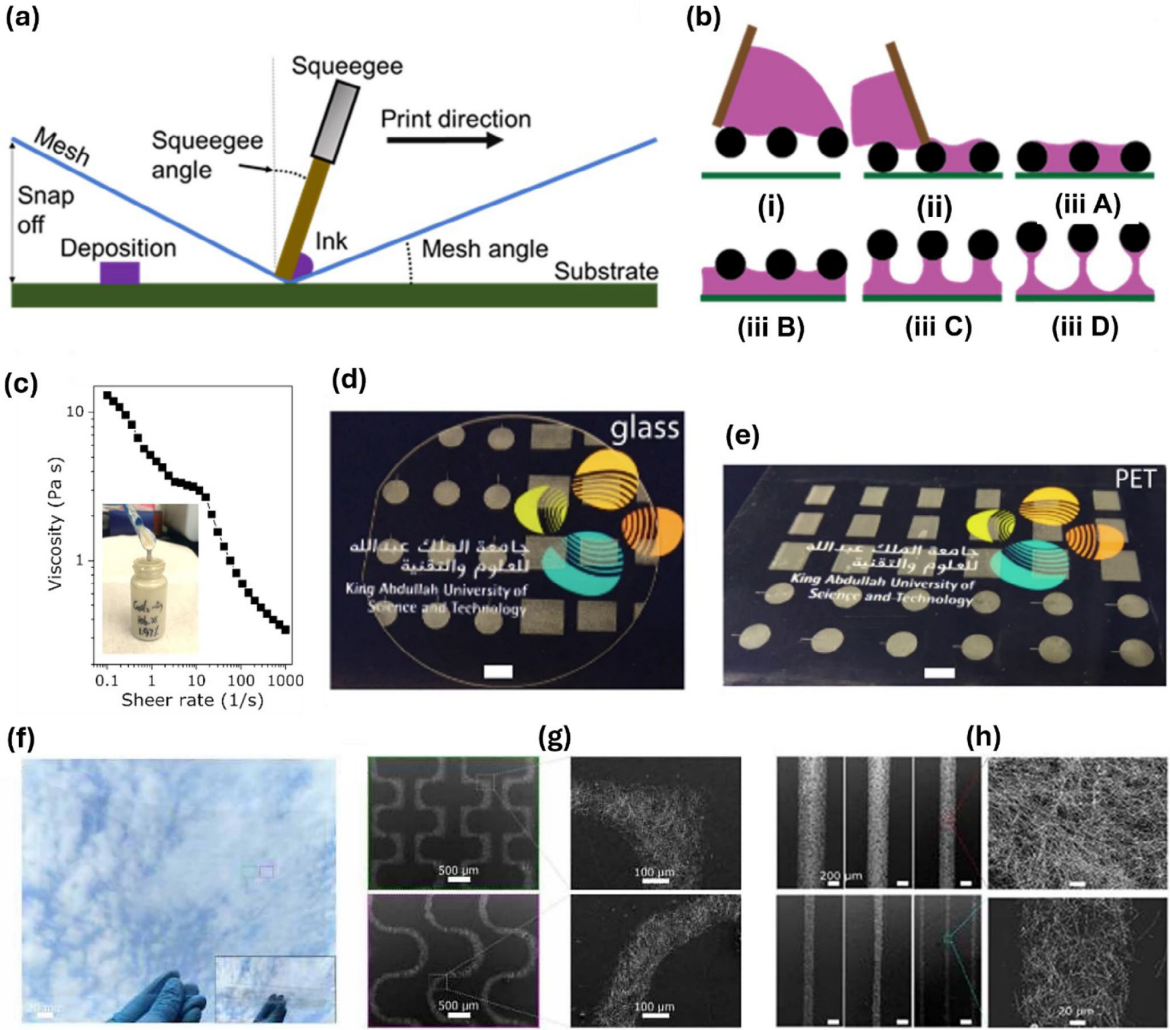
Screen printing. a) Schematic illustration of the screen‐printing process. b) The three‐stage mechanism of transferring the ink from the screen to the substrate. Reproduced with permission.^[^ ^132c^ ^]^ Copyright 2021, American Chemical Society. c) Viscosity of the prepared Ag NW ink (inset) at 0.1 to 1000 s^−1^ shear rate, confirming the shear‐thinning behavior of the ink. Screen printed Ag NW patterns on flexible d) rigid glass substrates and e) PET. Scale bar 10 mm. Reproduced under the terms of CC‐BY.^[^ ^152^ ^]^ Copyright 2019, Springer Nature. Large‐area and high‐resolution printing of Ag NWs: f) photograph of screen‐printed Ag NW patterns on a PET substrate, showing 92% optical transmittance at 550 nm and flexibility (inset). SEM images of screen‐printed Ag NW patterns with detailed edge views (right panel): g) serpentine and spiral patterns. h) lines with widths ranging from 500 µm to 50 µm. Reproduced under the terms of CC‐BY.^[^ ^132d^ ^]^ Copyright 2020, IOP Publishing.

Screen printing is a conventional contact printing process where material ink is transferred through a mesh screen to the substrate. The mesh screen has impermeable regions to block the ink from printing in selected areas, allowing direct patterning. High viscous (10^2^ to 10^5^ mPa. s) inks with shear‐thinning rheology are required.^[^ ^150^ ^]^ When a blade (squeegee) moves across the screen, the ink fills the screen openings and is printed onto the substrate by capillary force (**Figure** **10**a).^[^ ^150^ ^]^ The technique can be elaborated into a three‐stage printing mechanism.^[^ ^132^, ^151^ ^]^ In the initial stage, the ink is flooded into the mesh and filled in the open areas, as shown in Figure 10b(i). Based on the internal free energy of the system, the ink adheres to both the mesh and the substrate, and the mesh due to the downward force when squeezed with the blade (Figure 10b(ii)). The ink adheres to both the mesh and the substrate (Figure 10b(iiiA)), and when the mesh is pulled vertically the ink structure extends (Figure 10b(iiiB)), forming a filament structure (Figure 10b(iiiC)), and finally separate to form a film on the substrate, with a small portion remains in the mesh (Figure 10b(iiiD)). Due to the high ink viscosity, the quality of the film is minimally affected by the substrate surface properties, making this method widely implemented in textile manufacturing.^[^ ^10^ ^]^ The resolution of the printed pattern depends on the mesh size of the screen and the rheology of the ink. The surface tension and wettability of the ink also influence substrate adhesion, which can be controlled by the addition of binders, additives, and suitable solvent selection.^[^ ^132b^ ^]^

A wide variety of functional materials can be deposited using this technique for TC applications, including metal inks such as Ag and Cu, carbon‐based materials (Graphene, CNTs), polymer‐based PEDOT:PSS, and dielectrics.^[^ ^150^ ^]^ Ag NW dispersed in ethanol and with ethylene cellulose (EC) and polyvinyl pyrrolidone (PVP) as binders have been reported (Figure 10c). The shear‐thinning behavior of the dispersion with 3200 mPa s viscosity at 10 s^−1^ shear rate, which is suitable for screen printing.^[^ ^152^ ^]^ The ink can be directly used in screen printing and Figure 10d,e demonstrates the reliable patterns with various shapes and sizes printed on glass and flexible PET substrates. After optimizing the Ag loading (2 wt%), printing speed (>10 mm s^−^), squeeze pressure, number of cycles (1‐3) and followed by laser treatment, the film showed an excellent sheet resistance of 1.9 Ω sq^−1^ and transmittance of 73% at 550 nm (with ϕ_*TC*_ = 22.6 mΩ^−1^).^[^ ^152^ ^]^ Although simple and reliable, achieving high‐resolution and ultrathin films are the major challenges of traditional screen printing technology.

Screen printing can offer significant potential for the development of transparent electronics due to its scalable fabrication process.^[^ ^132a–c^ ^]^ For instance, integrating screen printing with flash‐light sintering (FLS) has been demonstrated for the large‐area, high‐resolution patterning of Ag NWs.^[^ ^132d^ ^]^ Figure 10f presents a photograph of screen‐printed patterns of Ag NWs on a flexible PET substrate with a dimension of 200 × 200 mm^2^, achieving low sheet resistance (1.1–9.2 Ω sq^−1^) and high transparency (75.2–92.6%).^[^ ^132d^ ^]^ The SEM image presented in Figure 10g clearly demonstrates well‐defined printed Ag NW patterns with precise and smooth edges. Additionally, printed Ag NW lines with various line widths were successfully achieved, with the narrowest line measuring approximately 50 µm, as shown in the SEM images in Figure 10h. In recent years, it is worth noting that mass production has been shifting towards adopting fine lines of 50 µm or smaller. As a result, screen printing has the potential to compete with digital printing techniques like inkjet printing, which can achieve even narrower line widths.^[^ ^132e^ ^]^ However, when compared to other printing technology such as inkjet printing, screen printing offers the advantage of working with a broader range of ink with high viscosities, enabling the use of more diverse and potentially more functional materials. While inkjet printing is valued for its higher resolution, it is typically limited by the low viscosity of inks it can handle where the possibility of getting the nozzle clogged still highly exist,^[^ ^132a^ ^]^ which can constrain the range of materials used and may lead to certain limitation with material properties and performance.

#### 4.1.5 Gravure Printing

**Figure 11:**
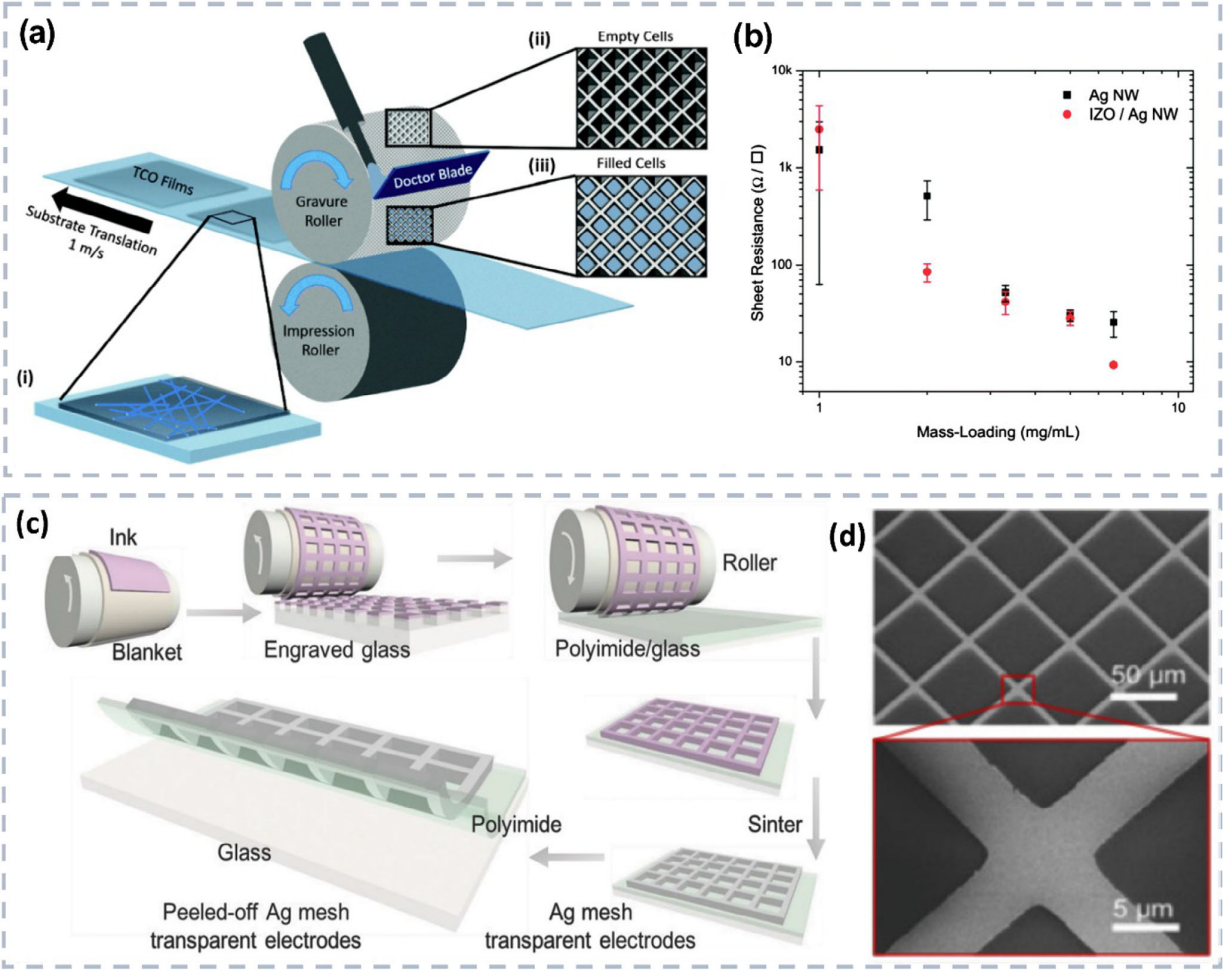
Gravure printing technique. a) Illustration of gravure printing to print conductive hybrid ink. b) The electrical performance of the fabricated transparent conductors with different mass loading of A NWs.^[^ ^133c^ ^]^ c) Schematic of the reverse gravure offset printing of Ag mesh transparent electrodes. d) SEM images of the printed Ag mesh with different magnifications. Reproduced with permission from.^[^ ^133d^ ^]^ Copyright 2018, John Wiley and Sons.

Gravure printing, also known as rotogravure printing, is a high precision, high‐speed contact printing technique that employs the direct transfer of functional inks through physical contact of the engraved structures (on cylinder or plates) and the substrate.^[^ ^153^ ^]^ This method involves the use of recessed cells on cylinder or planar plates to create patterns and can be implemented in both roll‐to‐roll (R2R) and plate‐to‐roll systems. Compared to other printing methods, such as drop‐on‐demand inkjet printing, gravure printing stands out for its ability to produce high‐resolution (sub‐10 µm)^[^ ^154^ ^]^ patterns at very high throughput (>10 ms^−1^) in a cost‐effective manner. This high‐speed process can cover large areas (greater than 1 meter wide and up to 1 kilometer long) at speeds up to 2000 feet per minute),^[^ ^153^, ^155^ ^]^ making it particularly suitable for the high‐volume production of printed electronics, including sensors, solar cells, and transistors.^[^ ^156^ ^]^ The benefits of this method arise from the utilization of microscale cells etched into the smooth surface of a solid metallic cylinder, which allows for the precise printing of controlled ink volumes (**Figure** **11**a).^[^ ^133c^ ^]^ The versatility of gravure printing is enhanced by its ability to handle a wide variety of inks with different functionalities including dielectric, metal oxides, semiconducting, and metallic^[^ ^153^, ^154^ ^]^ with viscosities (1–1500 mPa s).^[^ ^153^, ^157^ ^]^ This capability allows for the printing of inks with varying thickness, enabling the fabrication of devices with diverse functionalities, such as LEDs, RFID tags, and transistors. Gravure printing systems can operate in either forward or reverse mode.^[^ ^153f^ ^]^ In the forward mode, the substrate and roll move in the same direction through the engraved cells filled with ink, while in the reverse mode, the roll and substrate move in opposite directions. An alternative method, known as inverse direct gravure printing, uses a flat plate instead of a roll to transfer patterns to a substrate.^[^ ^158^ ^]^ This reverse and inverse modes of operation are gaining attention because of their improved operational stability.^[^ ^153^, ^159^ ^]^ Recent advancements in gravure printing technology have shown significant potential for applications in flexible and transparent large‐area electronics. For instance, studies have shown that gravure printing can produce highly conductive and transparent electrodes, which are crucial for the advancement of flexible displays and photovoltaic cells.^[^ ^133a,b^ ^]^ High‐throughput patterning and enhanced mechanical stability are essential for large‐area applications of metal nanowire mesh transparent electrodes. In this context, hybrid transparent conductors based on Ag NWs embedded in mechanically robust metal oxide matrix of IZO have been successfully demonstrated using high‐speed gravure printing (1.0 m s^−1^). These conductors exhibit excellent conductivity with 9.3 Ω sq^−1^ sheet resistance (Figure 11b), and high transparency (≈91% at 550 nm), along with robust mechanical properties.^[^ ^133c^ ^]^ An example of reverse‐offset printing technique, capable of realizing a uniform, ultrathin Ag mesh (100 nm) on flexible substrate is shown in Figure 11c.^[^ ^133d^ ^]^ By adjusting the spacing between adjacent lines, the conductivity and transparency of these Ag mesh electrodes can be precisely controlled. SEM images of printed Ag mesh are shown in Figure 11d, confirming well‐structured grids. This method achieved a sheet resistance of 17 Ω sq^−1^ and a transmittance of 93.2%, exceeding the performance of sputtered ITO electrodes. These advancements highlight the potential of gravure and reverse‐offset printing techniques in producing high‐quality, transparent electrodes suitable for next‐generation flexible and transparent electronics.

### 4.2 Emerging Printing Techniques

#### 4.2.1 Transfer Printing

**Figure 12:**
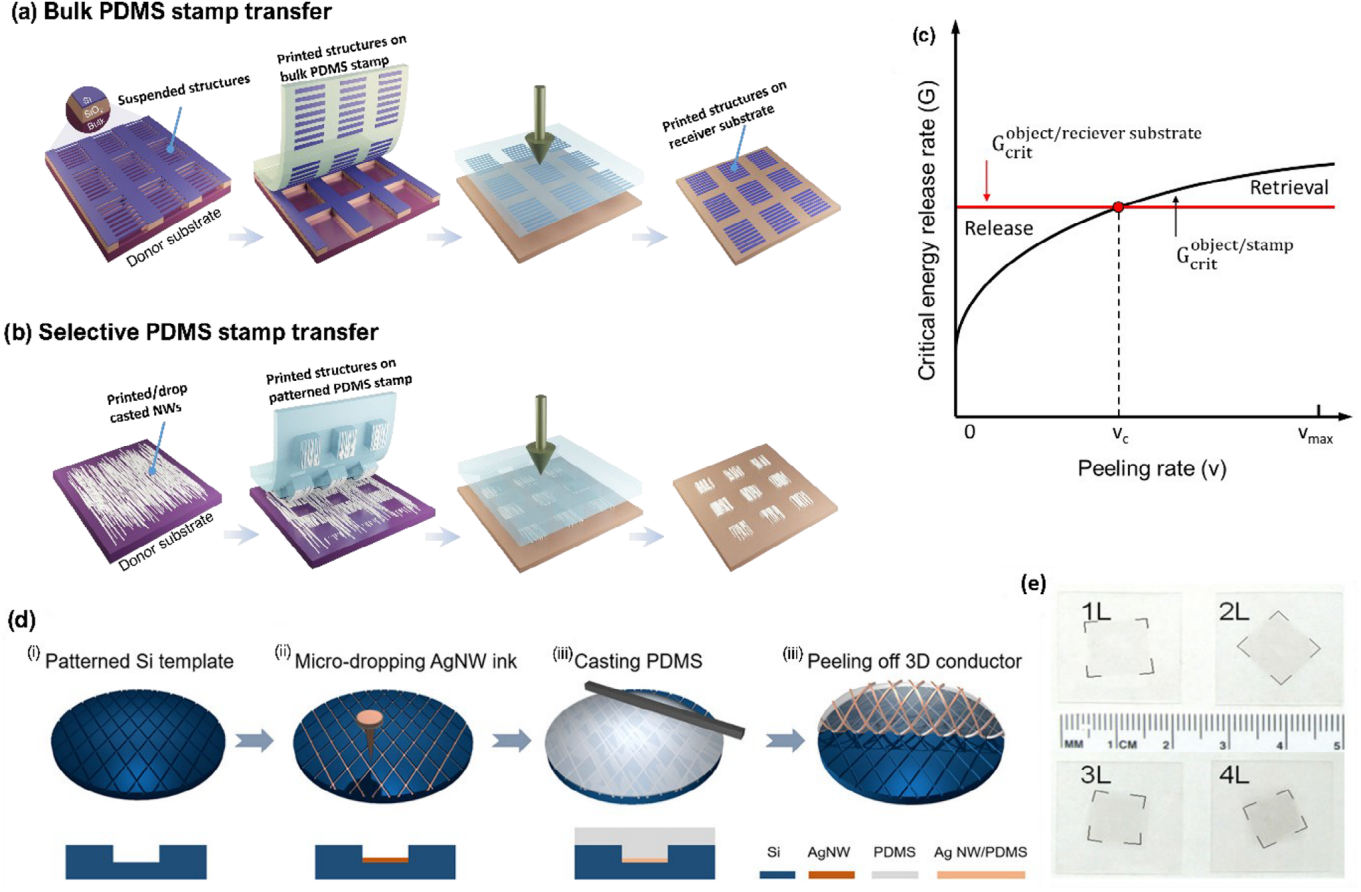
Transfer Printing method. a) Transfer printing of Si nanoribbons using a bulk viscoelastic stamp. b) Selective transfer printing of nanowires using patterned viscoelastic stamps with pillars. Reprinted under the terms of CC‐BY.^[^ ^165^ ^]^ Copyright 2022, John Wiley and Sons. c) Schematic representation of adhesion strength modulation by external stimulus during retrieval and printing. The extended peel velocity range of a PDMS‐based stamp (From 0 to V_max_ on the *x*‐axis) enables compatibility with wide range of substrates and receiver materials, (change in $G_{\mathrm{crit}}^{\mathrm{object}/\mathrm{reciever}\;\mathrm{substrate}}$, *y*‐axis). Reproduced under the terms of CC‐BY.^[^ ^166^ ^]^ Copyright 2007, American Chemical Society. d) Template‐assisted transfer printing process for the fabrication of transparent conductors. Reprinted with permission from.^[^ ^134^ ^]^ Copyright 2019, American Chemical Society. e) Photograph of 1 cm^2^ films of the TC graphene of 1–4 layers sequentially transferred with PMMA assistance to cover the glass receiver. Reprinted with permission from.^[^ ^61^ ^]^ Copyright 2009, American Chemical Society.

Transfer printing addresses several limitations associated with conventional solution‐based printing techniques, such as the non‐uniformity of printed material and the consistency in device‐to‐device performance. Traditional methods for fabricating ultrathin transparent high‐quality semiconducting layer such as Si and graphene or metallic materials on flexible polymeric substrates are hindered by extreme processing requirements, including high‐temperature annealing and chemical etching. Transfer printing offers alternative by allowing independent fabrication of devices structures (referred to as inks) on wafer donor substrates, which are subsequently transferred and assembled onto flexible or stretchable substrates with uniformity. This technique utilizes a soft and elastomeric stamp, typically made of polydimethylsiloxane (PDMS), to mediate the physical transfer of devices. Notably, transfer printing of Si‐based micro/nanostructures, such as nanowires, nanorods, and nanoribbons, from silicon‐on‐insulator (SOI) wafers to highly flexible substrates has been successfully demonstrated (**Figure** **12**a).^[^ ^160^ ^]^ As illustrated in Figure 12a,b, the process involves two key steps: retrieval or pick‐up process of micro/nanostructures from the donor substrate and stamping or printing them on to the receiver substrate. Efficient pick‐up requires that the stamp/ink/NW interface be stronger than the ink/donor interface, which can be achieved through techniques such as chemical etching^[^ ^160a^ ^]^ and lift‐off^[^ ^161^ ^]^ methods to weaken the ink/donor interface. Applying a preload to the stamp ensures sufficient adhesion to the inks. Recently, direct patterning of NWs on the growth substrate has been reported to further patterning after the transfer steps.^[^ ^15^, ^162^ ^]^ This new development helps to reduce the number of fabrication process steps and avoids the use of transfer steps and hence the chances of having leftover residues from the stamps. The residues from transfer stamps could adversely impact the optical and electrical properties of the devices developed using transferred materials.^[^ ^163^ ^]^

For efficient printing, the ink/nanostructure/receiver interface must be stronger than the stamp/nanostructure/ink interface. The success of the transfer printing process critically depends on modulation the stamp/ink interface adhesions (Figure 12c), which can be controlled using external stimuli such as the peel velocity and lateral movements.^[^ ^164^ ^]^ For instance, 3D stretchable TC was demonstrated by transfer printing Ag NWs, resulting a sheet resistance of 1 Ω sq^−1^ and 85% transmittance. This involved etching a netlike groove in Si wafer, microdropping the conducting Ag NWs into the groove, then transferring the pattern to PDMS (Figure 12d).^[^ ^134^ ^]^ Additionally, the transfer printing of multilayers of graphene, with precise layer controllability, has been demonstrated. Graphene layers grown on Cu foil via CVD were sequentially transferred to the receiver substrate assisted by PMMA layer. This technique produced graphene films with up to four layers (Figure 12e),^[^ ^61^ ^]^ achieving a sheet resistance of 350 Ω sq − 1 and nearly 90% of transmittance, which is higher compared to solution‐processed graphene assembly. However, transfer printing is not without its challenges. The stamp‐based process requires careful optimization of the stamp material and the adhesion properties at different interfaces to ensure high yield, high registration accuracy, and reliability. Additionally, while transfer printing is employed in realizing high‐quality, flexible electronic components, it may be less suitable for applications requiring large area and high‐throughput manufacturing compared to roll‐based printing techniques.

#### 4.2.2 Contact Printing of NWs

**Figure 13:**
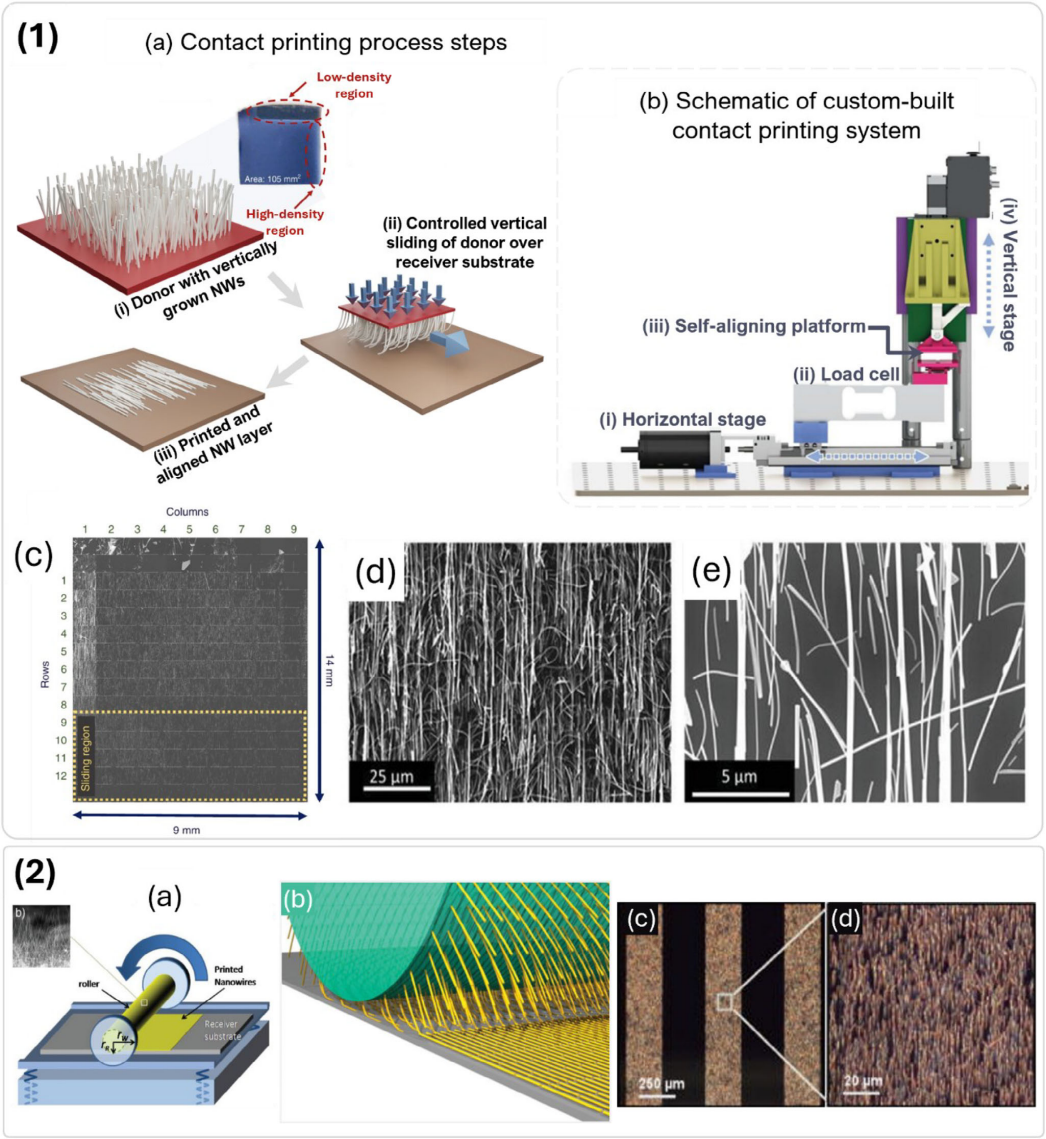
Contact printing of NWs. 1) Contact printing technique: a) Schematic showing contact printing process of vertical grown NW. b) Contact printing system setup. c) SEM of large area printed NWs on receiver substrate confirming high alignment. d, e) SEM image of the printed NWs with different magnification, showing uniform NW orientation and confirming the good alignment achieved. Reproduced under the terms of CC‐BY.^[^ ^27^ ^]^ Copyright 2021, Springer Nature. 2) Differential roll printing: a) 3D schematic representation of the differential roll printing process using cylindrical donor. b) Illustration of the interface between the cylindrical donor and planar receiver substrates during the direct roll process. Reproduced with permission.^[^ ^135e^ ^]^ Copyright 2009, John Wiley and Sons. c, d) Optical images of uniformly printed NWs on Si/SiO_2_ substrates using the differential roll printing technique. Reproduced with permission.^[^ ^135d^ ^]^ Copyright 2007, AIP Publishing.

Contact printing is one of the emerging non‐conventional printing technique, particularly adapted for transferring vertically grown quasi‐one‐dimensional materials, such as NWs, from a donor substrate to a receiver substrate without the need for an elastomeric stamp.^[^ ^167^ ^]^ It is highly challenging to synthesize NWs of similar dimensions and assembles them with uniform orientation over a large area. The high temperature or chemical etching requirements to grow NWs are also incompatible with conventional flexible polymer substrates. This makes contact printing a perfect solution to address these difficulties. **Figure** **13**(1a) illustrates the potential of contact printing technique to realize large area, aligned NW networks, which are critical for scalable, high‐performance transparent electronics. In this technique, NWs are grown with vertical orientation on a rigid donor substrate using traditional synthesis technique. The donor substrate is then brought into contact with the receiver substrate, which can be flexible, and pressure is applied between the two substrates. As the donor substrate slides over the receiver, the vertically grown NWs detach from the donor and are printed onto the receiver horizontally, aligned in the sliding direction.^[^ ^27^ ^]^ Key parameters, such as the applied force and the sliding velocity, must be optimized to control the printing quality. Additionally, treating the receiver surface chemically or with O_2_ plasma can further enhance the efficiency of NW transfer and alignment.^[^ ^168^ ^]^ Figure 13(1c) presents the stitched SEM image of a NW‐printed receiver sample, processed at 33 kN pressure and 1 mm s^−1^ sliding velocity, demonstrates regions of both low and high NW density that mirror the donor substrate (Figure 13(1a(i)) shows the donor substrate), confirming the efficient transfer. Highly aligned NWs are clearly visible in these regions. Furthermore, the primary advantage of the contact is its ability to print high‐density, well‐aligned NWs over a large area in a single step. This method is compatible with a wide variety of materials.^[^ ^135a–c^ ^]^ As shown in Figure 13, The process of contact printing allows for the selective transfer of high‐density, aligned nanowires onto flexible and transparent substrates, achieving device architectures compatible with transparent electronics.

The contact printing of Si NWs with a diameter of 115 nm and ZnO NWs with a diameter of 100 nm, achieving a high NW density of ≈7 NWs µm^−1^ and NW to NW spacing of 165 nm, has been demonstrated for transparent photodetection application.^[^ ^169^ ^]^ The NW length, morphology, and crystalline structure were preserved with high NW density and low NW to NW spacing over both rigid (Si/SiO_2_) and flexible (polyimide) receiver substrates. This capability to produce highly dense and consistently aligned NW films over large areas makes contact printing a promising technique for manufacturing transparent electronics. Contact printing is particularly resource‐efficient due to its lithography‐free process,^[^ ^165^ ^]^ which minimizes material waste and reduces electronic processing costs. Compared to other conventional techniques, e.g. inkjet printing and screen printing, contact printing provides superior alignment and density control over nanostructures, which are critical for the performance of transparent electronic devices. While other methods such as transfer printing and roll‐based processes are also explored for large‐area fabrication, contact printing offers distinct advantages in terms of simplicity and efficiency. The differential roll printing technique further extends contact printing to a roll‐based manufacturing process, adopting a roll‐to‐planar approach.^[^ ^135d^ ^]^ This method utilizes cylindrically shaped donor substrates, such as glass or quartz tubes, as NW growth substrates. The NW‐bearing cylinder is rolled over a planar substrate to transfer and align the NWs.^[^ ^135d,e^ ^]^ The performance of differential roll printing is comparable to conventional contact printing as shown in Figure 13(2a,b). Both methods have demonstrated the ability to fabricate 2D and 3D devices with similar NW densities, wafer‐scale NW transfer, and compatibility with both rigid and flexible substrates (Figure 13(2c,d)).^[^ ^9c^ ^]^ In terms of large‐area NW transfer, differential roll printing naturally has an edge over conventional contact printing.

#### 4.2.3 Roll‐Based Printing Techniques

**Figure 14:**
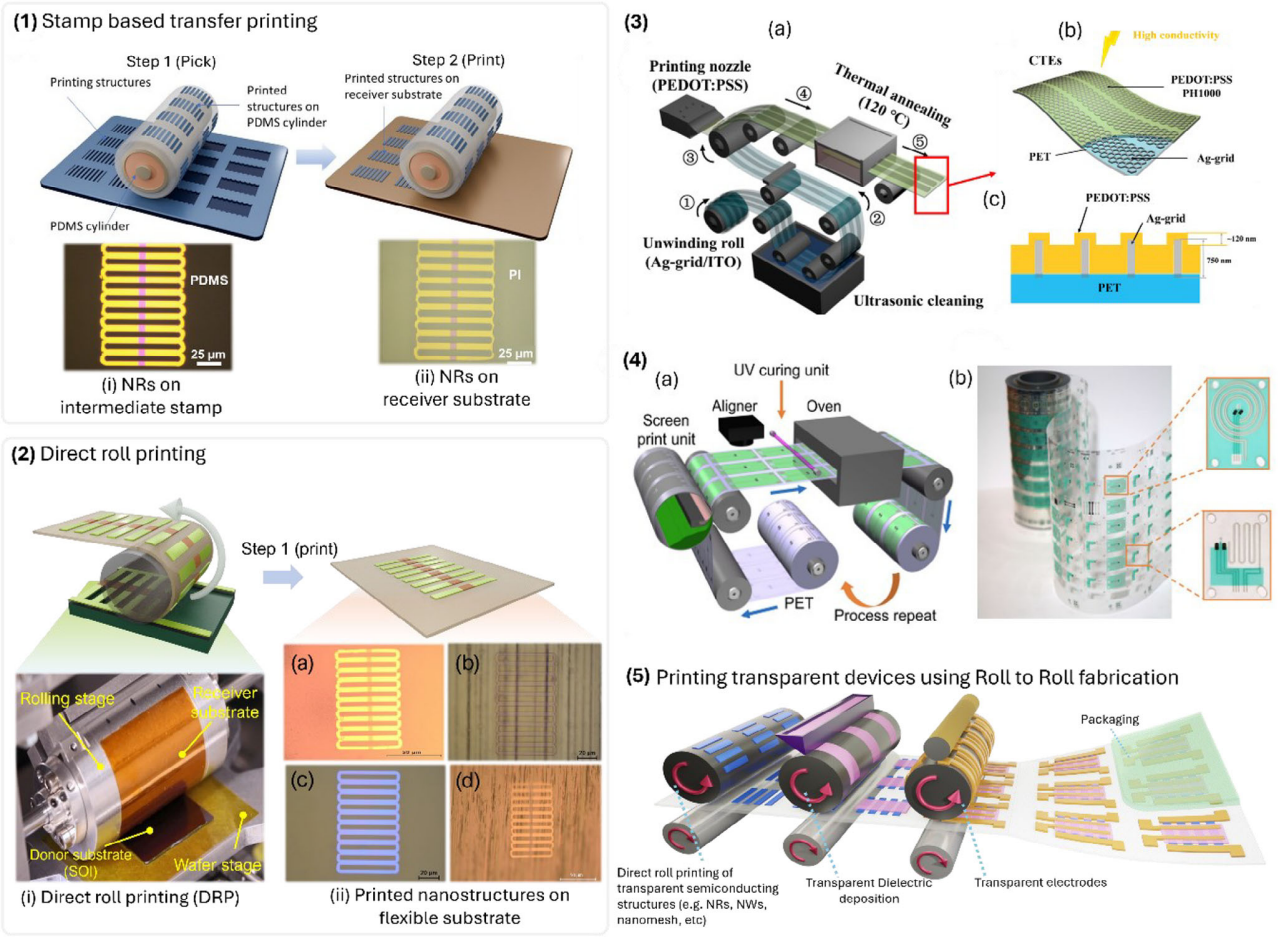
Roll‐based printing techniques. 1) Stamp‐based transfer printing of Si nanostructures using roll‐based PDMS stamp with optical microscopy images of each step. 2) Direct roll printing (DRP) process with optical microscopy images, showing the DRP of transparent Si NR arrays (70 nm thick) on: a) transparent PI, b) aluminium (Al), c) PET, and d) copper (Cu), and d) magnesium (Mg)). Reproduced under the terms of CC‐BY.^[^ ^160d^ ^]^ Copyright 2021, Springer Nature. 3) R2R printing of large area (meter scale) of PEDOT:PSS/Ag‐grid/PET composite TC electrodes. a) Schematic of R2R system. b) Schematic structure of the TC electrode and c) its cross‐sectional view. Reprinted with permission.^[^ ^175^ ^]^ Copyright 2018, American Chemical Society. 4) R2R rotary screen printing. a) schematic of R2R‐fabricated sweat sensing patches and b) optical images of sensing electrode patterns printed via R2R processing. Reproduced with permission.^[^ ^178^ ^]^ Copyright 2019, The American Association for the Advancement of Science. 5) Schematic demonstration of integrating DRP with Roll‐to‐Roll technology for realizing flexible and transparent large‐area electronics.

Expanding on the recently developed printing techniques, direct roll printing (DRP) offers a transformative approach for large‐area flexible and transparent electronics; by utilizing roll‐to‐roll (R2R) processing technology, DRP has shown substantial promise for enabling high‐throughput at relatively low costs, particularly due to its ability to print nanoscale structures over large areas, thereby making it highly attractive for transparent electronics applications. DRP stands out for its ability to transfer micro and nanoscale structures without the need for soft stamps, as seen in traditional transfer printing methods. The absence of these stamps simplifies the process and enhances registration accuracy. Recent studies have demonstrated that DRP can achieve a high transfer yield of 95% for silicon nanoribbons onto various substrates without requiring elastomeric stamps, thus highlighting its efficiency.^[^ ^160^, ^170^ ^]^ This process is further illustrated in (**Figure** **14**(1,2)), which present the key steps involved in both roll stamp‐based transfer printing and DRP. Furthermore, the elimination of elastomeric stamps in DRP reduces material waste, contrasting sharply with traditional transfer printing techniques that often involve significant material loss. Figure 14 (2) schematically illustrates the DRP process, where a flexible substrate coated with a semi‐cured thin polyimide (PI) film is rolled, and nanoribbons are gently released, allowing them to come into conformal direct physical contact with the receiver substrate for efficient transfer. The DRP process is well‐suited for large‐area fabrication, offering uniform and continuous electronic layer manufacturing over various substrates. This characteristic is essential for flexible displays, solar cells, and sensor platforms, where uniformity and scale are critical. For instance, silicon nanoribbons with a thickness of less than 100 nm can achieve optical transmittance of over 85%, making them suitable for transparent electronics applications.^[^ ^171^ ^]^ The fabrication process for achieving engineered transparent structures such as Si meshes, networks, NRs, etc. has been investigated for flexible transparent electronics, highlighting the potential of DRP in this domain. Optimizing the contact force in DRP ensures sufficient adhesion and conformal contact, thereby improving transfer yield. Direct roll printing offers several advantages, including high registration accuracy, reduced printing time, and lower fabrication costs. Direct roll printing of structurally transparent Si NR arrays has been achieved on various substrates without the need for an elastomeric stamp.^[^ ^172^ ^]^ The process involves several critical steps: fabricating suspended micro/nanostructures on the donor substrate, preparing the receiver substrate, optimizing printing parameters, and performing post‐printing processing. The donor substrate, typically a rigid wafer, experiences micro/nanofabrication processing steps to create ultra‐thin structures such NRs, where thin silicon ribbons can exhibit excellent transparency. Surface treatment of the receiver substrate, often using solutions such as semi‐cured PI, ensures better transfer yield. However, the use of PI as an adhesive layer may not always be compatible with transparent electronic layers, necessitating alternatives such as UV‐curable polymers for enhanced transparency and substrate compatibility. Despite this, the DRP process has demonstrated the capability to print Si NRs with 70 nm thickness on different flexible substrates, including transparent PI, polyethylene terephthalate (PET), and metal foils (e.g., aluminum (Al), copper (Cu), and magnesium (Mg)), (Figure 14(2a–d)), expanding its applicability for various flexible substrates. The DRP process has demonstrated the capability to print Si NRs with a thickness of 70 nm on different flexible substrates, including PET, transparent PI, and metal foils such as Al, Cu, and Mg (Figure 14(2a–d)). To enhance transparency further and expand substrate compatibility, UV‐curable polymers can be employed instead of semi‐cured PI. These adhesives not only facilitate accelerated printing, reducing the overall process duration, but also offer improved transfer yields. For example, UV‐curable adhesives enable the transfer process on a wide range of substrates, including those with varying surface roughness and mechanical properties.^[^ ^160^, ^173^ ^]^

Direct roll printing's ability to integrate with R2R processes enhances its potential for large‐area fabrication and micro/nanoscale integration necessary to transform and upscale from lab‐scale to large‐scale commercialization for flexible and transparent electronics. The technology offers high throughput, high‐speed production, and low costs, making it highly beneficial.^[^ ^174^ ^]^ For instance, roll printing's ability to realize cost‐effective electrodes, such as PEDOT and Ag grid on PET substrates, demonstrates its economic and material printing efficiency. Composite electrodes fabricated via slot‐die R2R technology exhibit significant cost saving ($15–20 m^−2^) compared to traditional ITO/PET electrodes ($50 per square meter), demonstrating both economic and material printing efficiency. The process for fabricating these electrodes involves several stages, including cleaning and surface treatment of substrates, optimizing printing parameters, and post‐printing treatments to enhance conductive performance, as shown in Figure 14 (3). Figure 14(3a) shows the schematic of the fabrication of a composite transparent electrode consisting of PEDOT:PSS and Ag grid on PET substrate by slot‐die roll‐to‐roll technique. Figure 14(3b) shows the composite electrode schematic structure and Figure 14(3c) shows the cross‐sectional view of the composite electrode with the polymer covering the Ag‐grid.^[^ ^175^ ^]^ Thermal evaporation techniques can also be incorporated with R2R technology.^[^ ^176^ ^]^ R2R fabrication of 18 nm thick Ca:Ag transparent electrodes by co‐evaporation was recently reported.^[^ ^177^ ^]^ The electrode exhibited 64% transmittance and 21 Ω sq^−1^ sheet resistance and observed suitable as top‐electrode for OLED fabrication. Similarly, recent advancements have demonstrated the feasibility of roll printing using R2R rotary screen printing for achieving high‐throughput fabrication of large‐area flexible electronics. A recent example of this capability is demonstrated by the fabrication of wearable biosensing patches.^[^ ^178^ ^]^ Schematic of R2R‐fabricated sweat sensing patches (Figure 14(4a)) and optical images of sensing electrode patterns printed via R2R processing (Figure 14(4b)) illustrate the potential of this technique for creating large‐area transparent flexible electronics. Similarly, in the domain of large‐area transparent electronics, DRP can be directly applied to transparent platforms using transparent materials, offering an attractive avenue for future transparent electronics. Notably, DRP is highly compatible with roll‐to‐roll manufacturing and holds promise for producing large transparent electronics. For instance, roll‐to‐roll printed devices on flexible substrates, depicted in Figure 14 (5), highlight the versatility and scalability of DRP for large‐area transparent applications.

### 4.3 Alignment/Assembly Techniques

#### 4.3.1 Dielectrophoresis (DEP)

**Figure 15:**
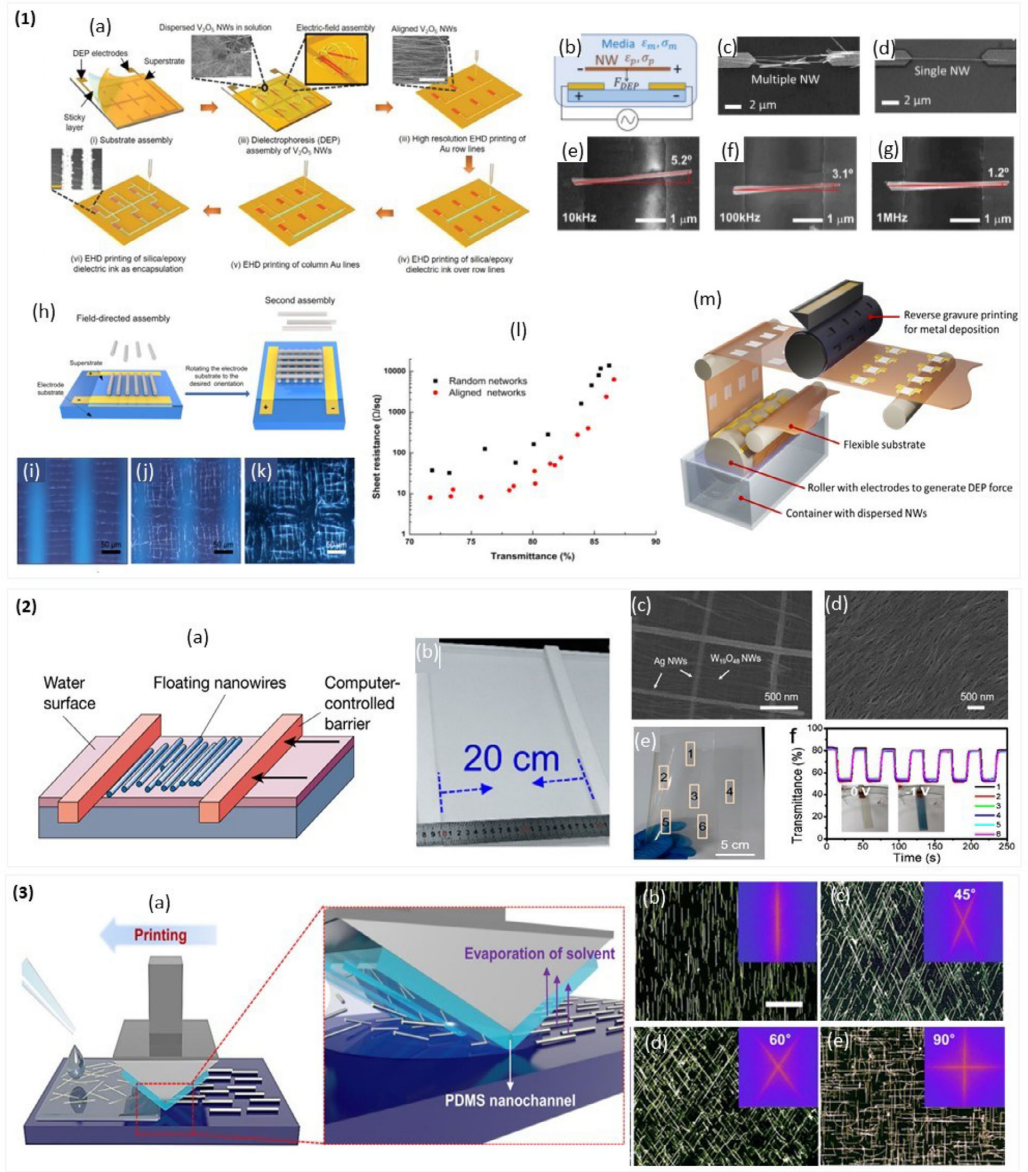
Aligned deposition of 1D materials. Alignment/assembly techniques for transparent electronics. 1) Dielectrophoresis (DEP) process, a) Schematics and SEM images presenting the fabrication process of artificial thermoreceptors utilizing V_2_O_5_ NWs aligned via DEP. Reproduced under the terms of CC‐BY.^[^ ^180^ ^]^ Copyright 2022, John Wiley and Sons. b) Schematic showing the mechanism of DEP; a suspended NW near the DEP electrodes. c) Multiple and d) single CuO NW interconnect formation across the electrodes achieved by controlling the applied DEP AC frequency range. Reproduced with permission.^[^ ^181^ ^]^ Copyright 2021, Elsevier. SEM image of the alignment of individual GaAs NW deposited by DEP of 7 V amplitude and e) 10 kHz, f) 100 kHz, and g) 1 MHz frequencies. Reproduced under the terms of CC‐BY.^[^ ^182^ ^]^ Copyright 2020, IOP Publishing. h) Schematic representation of the multi‐step nanowire assembly method. i–k) Dark‐field optical image of Multi‐directional assembly. (l) Sheet resistance versus optical transmittance (λ = 550 nm). Reproduced with permission.^[^ ^136c^ ^]^ Copyright 2018, IOP Publishing. 2) Langmuir–Blodgett (LB) technique, a) Schematic illustration of NWs assembly using LB technique. Reproduced with permission.^[^ ^137a^ ^]^ Copyright 2003, Springer Nature. b) photograph of large‐scale NWs assembly using LB alignment technique. c,d) SEM images of the co‐assembled hybrid nanowire networks. e) photograph of flexible transparent electrochromic film. f) cyclic switching behaviors of the electrochromic film corresponding to 6 different locations, as shown in (e). Reprinted with permission.^[^ ^137b^ ^]^ Copyright 2017, American Chemical Society. 3) capillary printing. a) schematic illustration of NWs alignments via capillary printing technique. b–e) highly aligned Ag NW network with different orientations. The scale bar is 40 µm. Reprinted with permission.^[^ ^138b^ ^]^ Copyright 2015, American Chemical Society.

Dielectrophoresis (DEP) is a critical technique for the alignment of one‐dimensional (1D) nanomaterials, such as metallic nanowires (NWs), which is an important factor in meeting the conductivity and transparency demand for flexible large‐area transparent electronics.^[^ ^132a^ ^]^ 1D‐nanomaterials dispersed in a solvent can be aligned by applying an external field such as electric and magnetic.^[^ ^179^ ^]^ DEP employs a non‐uniform electric field to induce a dipole moment in dispersed particles, and to align them between conducting electrodes. When subjected to either an alternating current (AC) or an inhomogeneous direct current (DC) field, the induced polarization separates charges on the NW surfaces. This process aligns the NWs orientation along the electric field of lines and attract them toward the electrodes. The alignment process is influenced by several factors, including the density of the NWs in the dispersion, the flow rate near the electrodes, the electrode configuration, and the frequency and amplitude of the AC signal. Precise control of these parameters is essential for optimizing the assembly rate and positioning accuracy of the NWs. Recent research has demonstrated the use of the dielectrophoretic (DEP) process for defined positioning and orienting V_2_O_5_ NWs at specific locations across large area. This method, as shown in the schematic in **Figure** **15**(1a), involves detailed DEP assembly steps for fabricating artificial thermoreceptors utilizing DEP‐aligned V_2_O_5_ NWs.^[^ ^180^ ^]^ Similarly, the alignment of CuO NWs between metallic electrodes has been successfully demonstrated.^[^ ^181^ ^]^ CuO NWs grown via thermal oxidation and dispersed in isopropanol, were aligned between lithographically patterned Au electrodes using an AC signal (Figure 15(1a)). It was demonstrated that the alignment efficiency was frequency‐dependent, with higher frequency (50–500 kHz) leading to a single NW assembly (Figure 15(1c)), and lower frequencies (<10 kHz) resulting in multiple NW assembly (Figure 15(1b)). This behavior is influenced by the dielectric constant of the NWs and the liquid media.^[^ ^181^ ^]^ In another example, semiconducting GaAs NWs achieved over 90% assembly yield and above 95% alignment yield (less than 5^○^ misalignment) by optimizing the AC signal's amplitude (4 to 7 V) and frequency (10 to 1000 kHz) as shown in Figure 15(1e‐g).^[^ ^182^ ^]^ This confirms that the high AC frequency is essential not only to assemble individual NWs but also to align them between the electrodes. DEP has been widely employed with various materials, including CNTs,^[^ ^183^ ^]^ ZnO,^[^ ^184^ ^]^ Ag,^[^ ^136^, ^185^ ^]^ V_2_O_5,_^[^ ^180^ ^]^ and Si^[^ ^186^ ^]^ NWs. This technique offers promising potential in the development of large‐area transparent electronics by ensuring precise alignment, which is crucial for electrical conductivity and reliability.^[^ ^132a^ ^]^ Accurate alignment is an important factor for transparent flexible electronics due to the requirements for scalability, and device uniformity. Techniques such as DEP enable the controlled placement of nanomaterials, which is essential for maintaining high electrical performance and transparency in flexible devices. In this regard, DEP has been employed for transparent applications by aligning conductive NWs, demonstrating its potential in scalable optoelectronic devices, such as transparent touchscreens.^[^ ^136a,b^ ^]^ DEP‐based multi‐step assembly of Ag NWs on a flexible substrate has been reported (Figure 15(1h‐k)), which achieves lower sheet resistance compared to randomly distributed NWs (Figure 15(1l)), highlighting the critical role of precise alignment.^[^ ^136c^ ^]^ Moreover, advanced roll‐based contactless DEP has been demonstrated (Figure 15(1m)).^[^ ^187^ ^]^ In the future, such techniques could enable the fabrication of complex multilayer structures, further expanding the capabilities of DEP in the next generation transparent and flexible electronics.

#### 4.3.2 Langmuir–Blodgett Assembly

The Langmuir‐Blodgett (LB) assembly technique is a well‐established method for aligning various nanomaterials, such as NWs, nanoparticles (NPs), and nanotubes, providing a potential approach for fabricating flexible and transparent large‐area electronics. This technique utilizes the “logs‐on‐a‐river” analogy, where the alignment of 1D nanomaterials occurs similarly to logs aligning along a narrow river due to flow direction constraints. Typically, 1D NWs diffusing on the surface of organic solvents form a randomly distributed, loosely stacked network (Figure 15(2a)).^[^ ^137a^ ^]^ By employing a baffle to control surface pressure and barrier velocity, a high‐density aligned Langmuir nanostructured film can be achieved, which is critical for various electronic applications requiring both transparency and high connectivity. Recent studies have highlighted the potential of Langmuir–Blodgett (LB) assembly in manufacturing transparent electronics on a large scale. For example, a transparent electrochromic film with dimensions of 20 cm × 16 cm has been successfully fabricated, as illustrated in Figure 15(1b). As shown in Figure 15(2c,d), this process involves the manipulation of highly dense and ordered networks of Ag and W_18_O_49_ NWs to create flexible, transparent electrochromic devices. Achieving tunable conductivity (7–40 Ω sq^−1^) and transmittance (58–86% at 550 nm) through the assembly of two layers of aligned NWs at crossing angles (Figure 15(2e,f).^[^ ^137b^ ^]^ This process, conducted under ambient temperature and pressure conditions, preserves the intrinsic properties of the materials. Similarly, another study demonstrated the large‐scale fabrication of flexible and transparent electrodes by co‐assembling Ag nanowires (NWs) with Te NWs using LB technique. After assembly, the Te NWs were etched away, resulting in a network of Ag NWs with a controllable pitch. This network forms a flexible, transparent conducting electrode with an average transmission of up to 97.3% and sheet resistances as low as 2.7 Ω/sq under optimized conditions.^[^ ^137c^ ^]^ Various materials, including Ag NWs,^[^ ^137c^ ^]^ Au NWs,^[^ ^188^ ^]^ VO_2_ NWs,^[^ ^189^ ^]^ and Ge NWs,^[^ ^190^ ^]^ have been successfully assembled using the LB technique, highlighting its versatility for NW assembly. However, the traditional LB technique has limitations, such as the need for surface functionalization of NWs, slow processing times, and stringent condition control to achieve uniform NW arrays over large areas. To address these challenges, modified LB techniques have been explored.^[^ ^132a^ ^]^ For example, a meniscus‐assisted float assembly method has been reported for Au NWs, resulting in a densely arranged, defect‐free NW monolayer with a thickness of 2 nm and a high transmittance of 96.5% at low sheet resistance (400—500 Ω sq^−1^).^[^ ^137d^ ^]^ Similarly, another attempt involved developing a shear‐assisted Langmuir technique for faster alignment of NWs, which achieves rapid and efficient alignment of NWs.^[^ ^191^ ^]^ These advancements in LB assembly techniques underscore the potential of this method in producing high‐quality, transparent electronics suitable for large‐area applications.

#### 4.3.3 Capillary Printing

Capillary printing has emerged as an effective technique for fabricating micropatterned electronic layers, employing solution shearing along a defined direction on the substrate. This method involves drop‐casting of the solution onto the substrate, where it is subsequently anchored and dragged parallel to the surface at specified intervals. This process utilizes nanopatterned polydimethylsiloxane (PDMS) stamps, which, under controlled velocity and pressure, create micropatterned thin layers over a large area. The efficacy of the micropatterns produced via capillary printing is largely dependent on the channel width of the nanopatterned PDMS. These channels confine the solution and induce an ordered assembly of nanostructures. To achieve a high‐quality micropatterned layer, parameters such as solution concentration, contact pressure between the PDMS stamp and the substrate, and printing speed must be carefully optimized.^[^ ^192^ ^]^ Despite the advantages, the PDMS stamp requires frequent replacement due to its incompatibility with various solvents, which increases overall material waste and limits its use for developing transparent electronics over large areas.^[^ ^18b^ ^]^ For metallic nanowire‐based transparent conductive electronic layers, a significant portion of the sheet resistance originates from the contact resistance between NWs. Minimizing this contact resistance is crucial for achieving high conductivity for large‐area transparent electronics.^[^ ^138a^ ^]^ In this regard, capillary printing offers an alternative approach, enabling the formation of junction‐free conductive NW networks. This is achieved by dragging nanopatterned PDMS stamp over metallic NW solutions on target substrates under consistent velocity and pressure (Figure 15(3a), resulting in highly aligned (Figure 15(3b)) or bi‐aligned Ag NW networks (Figure 15(3c–e)).^[^ ^138b^ ^]^ The alignment of Ag NW networks has been demonstrated using this approach with superior transparency (95.0–96.7%) and lower sheet resistance (15.6‐25.2 Ω sq^−1^) compared to random Ag NW networks (92.9%, 20 Ω sq^−1^).^[^ ^138b^ ^]^ The potential of capillary printing for transparent electronics is further highlighted by the enhanced performance of polymer light‐emitting diodes (PLEDs) and polymer solar cells utilizing aligned Ag NW electrodes. Overall, capillary printing is a promising technique for the development of flexible electronics, offering significant advantages in terms of pattern precision, transparency, and conductivity. The method's ability to produce high‐quality, aligned nanowire networks makes it worthy of further development toward the advancement of next‐generation transparent electronic devices.

Various printing technologies have been explored recently for fabricating transparent and conductive materials on flexible substrates, some of these techniques were discussed in this section. Direct patterned printing of transparent and conducting materials over a large area on a flexible substrate can be a challenging task, and clearly, a single fabrication process cannot satisfy all the requirements. Inkjet and screen‐printing technologies can be utilized when lateral resolution is vital over film quality. Transfer printing techniques facilitate the integration of inorganic and semiconducting materials with flexible substrates. Direct roll transfer printing, a hybrid approach combining R2R and transfer printing technologies, is an authentic promise in terms of mass production of uniform electronics over a large area. Emerging printing techniques such as roll‐based transfer printing, contact printing, and DEP technologies are still in their nascent stages, requiring further research and development to extend their applicability beyond NWs. Future advancements will likely focus on developing hybrid technologies compatible with diverse materials, ensuring printed material quality and device uniformity without compromising scalability or resource management. By optimizing alignment techniques such as DEP, the field of transparent flexible electronics can achieve significant advancements in performance and manufacturing efficiency, opening new avenues for innovation and application in various electronic devices.

## 5 Applications

The functional materials for transparent electronics and the advancements in printing technologies discussed earlier have paved the way for the development of new types of flexible, transparent, large‐area electronic devices. This section discusses some of the applications of transparent electronics, including transparent integrated circuits, sensors, interactive displays, actuators, and energy harvesters.

### 5.1 Electronic Circuits

**Figure 16:**
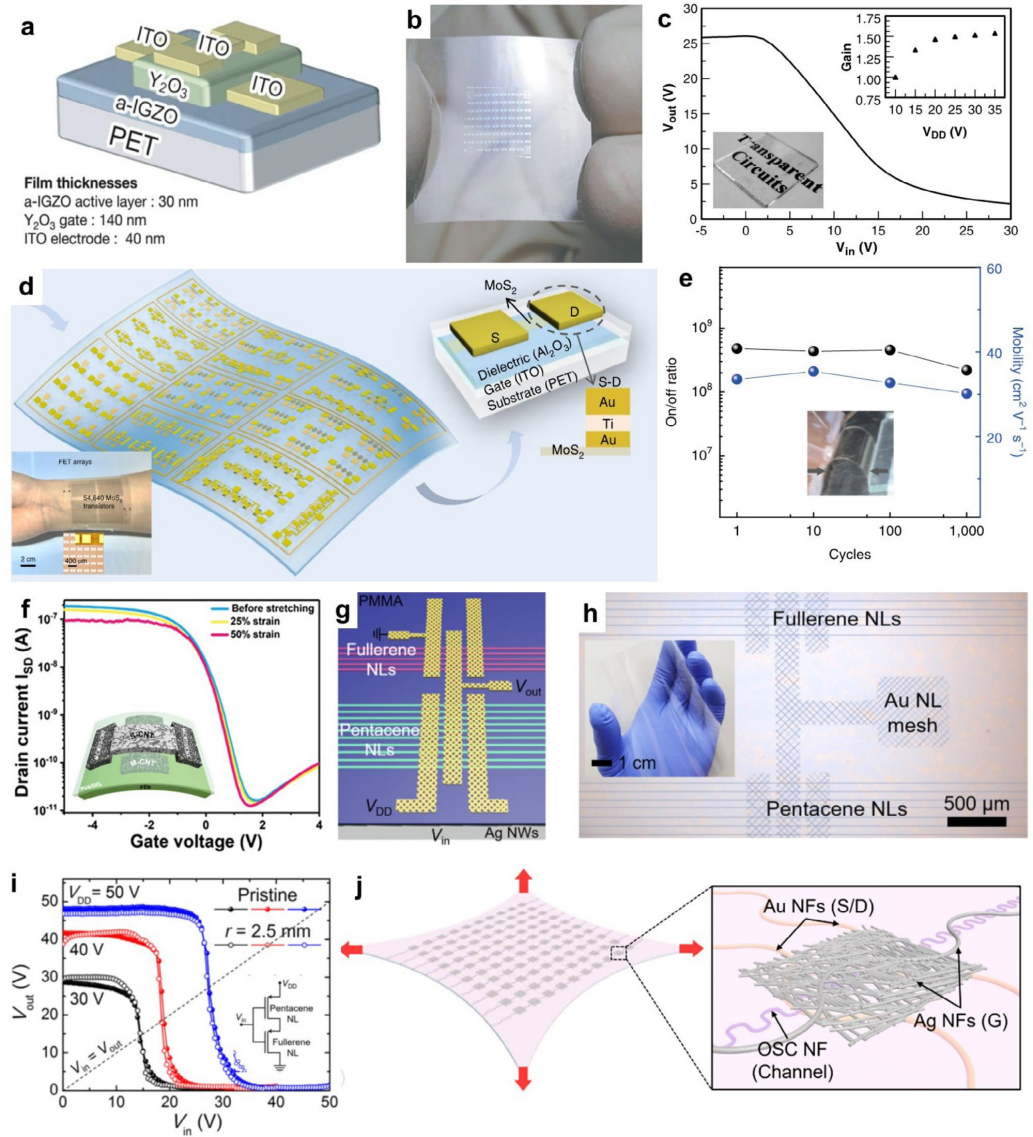
Transparent Electronic Circuits. a) Schematic and b) Photograph of the IGZO TFT on the PET substrate with the inset represents the transistor structure. Reprinted with permission.^[^ ^20^ ^]^ Copyright 2004, Springer Nature. c) Transfer characteristics and the gain for the transparent inverter fabricated using IGO TFTs. Photograph of a glass substrate with three inverters, two ring oscillators, and several transistors, confirming good transparency of the circuits. Adapted with permission.^[^ ^194^ ^]^ Copyright 2006, Elsevier. d) Schematic of flexible transparent MoS_2_‐based transistor arrays with integrated circuits and the specific structure of the MoS_2_ FETs. A photograph of MoS_2_ transistor arrays with 1518 transistors cm^−2^ device density fitted to a hand is shown in the inset. e) On–off ratio and device mobility as a function of bending cycles, confirming good flexibility of the devices. Reproduced with permission.^[^ ^202^ ^]^ Copyright 2020, Springer Nature. f) Transfer characteristics of all CNT‐based transistors under various tensile stress perpendicular to the channel direction. Inset shows the schematic structure of the transistor. Reprinted with permission.^[^ ^203^ ^]^ Copyright 2020, John Wiley and Sons. g) Schematic structure and h) optical microscopy of the NL FET‐based complementary inverter circuit array. i) The voltage transfer characteristics of the NL FET complementary inverter at pristine and bent states with the inset showing the circuit diagram. Reprinted with permission.^[^ ^31^ ^]^ Copyright 2020, American Chemical Society. j) Schematic structure of the highly integrated nanofiber‐based stretchable and transparent FET array. Reprinted with permission.^[^ ^204^ ^]^ Copyright 2021, American Chemical Society.

Transistors are the building blocks of every electronic circuit; they are three‐terminal devices in which the current through a semiconducting layer, positioned between two electrodes, is modulated by a third transverse or all‐around electrode, generally separated from the semiconductor by a dielectric. The first transparent thin‐film transistor (TFT) with 75% transparency, reported in early 2000s,^[^ ^193^ ^]^ using ZnO as *n*‐channel. This enhancement mode TFT exhibited a mobility of up to 2.5 cm^2^ V^−1^ s^−1^. Mobility is a crucial factor for efficient transistors, which measure electron movement efficiency under an electric field.^[^ ^183b^ ^]^ Higher mobility results in higher current, enabling rapid charging and discharging of capacitive loads, which is essential for high‐speed circuits. The source, drain, and gate contacts were made using sputter‐coated ITO, with ALD‐deposited aluminium‐titanium oxide as gate dielectric. In the following year, a flexible and transparent (>80%) *n*‐channel TFT on PET substrate using an amorphous In‐Ga‐Zn‐O (IGZO) system as the channel was reported (**Figure** **16**a,b), with improved mobility of 9 cm^2^ V^−1^ s^−1^. The channel, ITO contacts, and Y_2_O_3_ dielectric were fabricated at room temperature using PLD.^[^ ^20^ ^]^ The example among the first reported circuits includes a 75% transparent inverter and ring oscillator circuit developed using *n*‐channel indium‐gallium oxide (IGO) based TFTs.^[^ ^194^ ^]^ The inverter, consists of two n‐channel TFTs, a control transistor, and a load transistor. The voltage transfer characteristics of the inverter are shown in Figure 16c at 30 V bias, with a peak gain of 1.5. The ring oscillator, with five serially connected inverters, has a maximum oscillation frequency of 9 kHz. Other *n*‐channel oxides, including ZTO,^[^ ^195^ ^]^ IZO,^[^ ^196^ ^]^ IZTO,^[^ ^197^ ^]^ and IHZO,^[^ ^198^ ^]^ in addition to *p*‐channel transparent TFT based on CuO, Cu_2_O,^[^ ^199^ ^]^ and SnO,^[^ ^1a^ ^]^ have been reported. Combining *n* and *p* channel technologies can realize CMOS technology with lower power consumption and higher functional density. In 2011, a transparent CMOS was reported by utilizing the ambipolar property of the SnO,^[^ ^200^ ^]^ achieving significant switching abilities but still below par with the conventional electronics, and a boost is desired in terms of better carrier mobility. Metal oxide films exhibit limited flexibility due to their inherent properties.^[^ ^201^ ^]^

Recent attention has shifted to 2D semiconducting materials such as graphene and MoS_2_ due to low device yield and limited flexibility of semiconducting oxides. For example, epitaxially grown MoS_2_ has been transferred onto PET substrate pre‐deposited with ITO (gate electrode) and Al_2_O_3_ (dielectric).^[^ ^202^ ^]^ This work exhibited excellent device‐to‐device uniformity, a 97% yield, and a high density of 1518 transistors per cm^2^ were reported (Figure 16d)_._ The MoS_2_ transistors demonstrated an on/off ratio of ≈10^10^, a current density of ≈35 µA µm^−1^, and carrier mobility of ≈55 cm^2^ V^−1^ s^−1^, exceeding metal oxide‐based devices. These transistors remained reliable against mechanical deformations, maintaining mobility, and on/off ratio remained unchanged after 1000 bending cycles of 1% strain (Figure 16e). Flexible and transparent integrated circuits, including logic gates (inverter, NAND, NOR, AND), static random‐access memory (SRAM), and a five‐stage ring oscillator, demonstrated the high potential of 2D materials for high‐performance devices. CNTs, known for their mechanical and optoelectronic properties along with high carrier mobilities, have also been explored.^[^ ^205^ ^]^ A fully CNT‐based transparent and stretchable TFT^[^ ^203^ ^]^ has been reported with metallic and semiconducting CNTs as the electrode and channel, PVA hydrogel as the dielectric material, and PDMS as the substrate. Figure 16f shows the transfer characteristics, remaining functional after 1000 stretching cycles at 50% strain perpendicular to the channel, making them suitable for wearable technologies, due to their high stretchability.

Another approach involves constructing transistors using 1D nanostructures, where the dimension of the non‐transparent materials is reduced to make them appear as transparent (structural transparency). A nano‐line (NL) field‐effect transistor (FET) array has been demonstrated with pentacene, IZO, and fullerene deposited as NLs to form the semiconducting channel.^[^ ^31^ ^]^ Au nanomesh was patterned on top of semiconductor NLs for top contacts, while Ag NWs were spin‐coated over PET sheet as the gate electrode with PMMA as the dielectric. The nearly 90% transparent NL FET showed 0.52 cm^2^ V^−1^ s^−1^ mobility and 7 × 10^6^ on–off ratio, stable at a 2.5 mm bend radius. A NL FET‐based complementary inverter (Figure 16g) with a gain of 21 was implemented, demonstrating applicability for circuit implementations.^[^ ^31^ ^]^ The device's optical microscope image is shown in Figure 16h, and the transfer characteristics in Figure 16i indicate that NL devices can achieve comparable performance with that of continuous film‐based devices. An array of stretchable transparent nanofiber‐based FETs was demonstrated by electrohydrodynamic printing of metallic and organic semiconducting nanofibers (Figure 16j).^[^ ^204^ ^]^ Nano‐dimensional material‐based devices offer structural transparency, high flexibility, and short channel lengths, allowing faster switching. Despite predicted improvements, the electrical performance of transparent devices and circuits is still inferior to that of traditional Si technology.

In addition to transparency, the transient behavior of the devices is an important aspect, which is gaining interest, particularly because a significant amount of e‐waste is generated annually. Some of the examples in this direction include a vertical organic–inorganic hybrid transistor developed using biodegradable chitosan biopolymers.^[^ ^206^ ^]^ To further enhance the degree of sustainability of such devices, the glass substrate could be replaced with a flexible, biodegradable substrate, as discussed in Section 3. Another notable advancement in this area is reflected by the devices such as metal‐oxide‐based CMOS on nanopaper (nanocrystalline cellulose), which exhibited a mobility >7 cm^2^ V^−1^ s^−1^, marking a promising step toward fully biodegradable transistors.^[^ ^207^ ^]^ However, full transparency was not achieved due to the use of opaque Al thin films for the contacts. Another example is the flexible transparent and biodegradable electrolyte capacitor, offering 80% transparency and a capacitance on the order of millifarads per gram.^[^ ^208^ ^]^ More efforts are required to build on this momentum and develop fully transient, transparent electronic circuits in the future.

### 5.2 Sensors and Actuators

#### 5.2.1 Sensors

**Figure 17:**
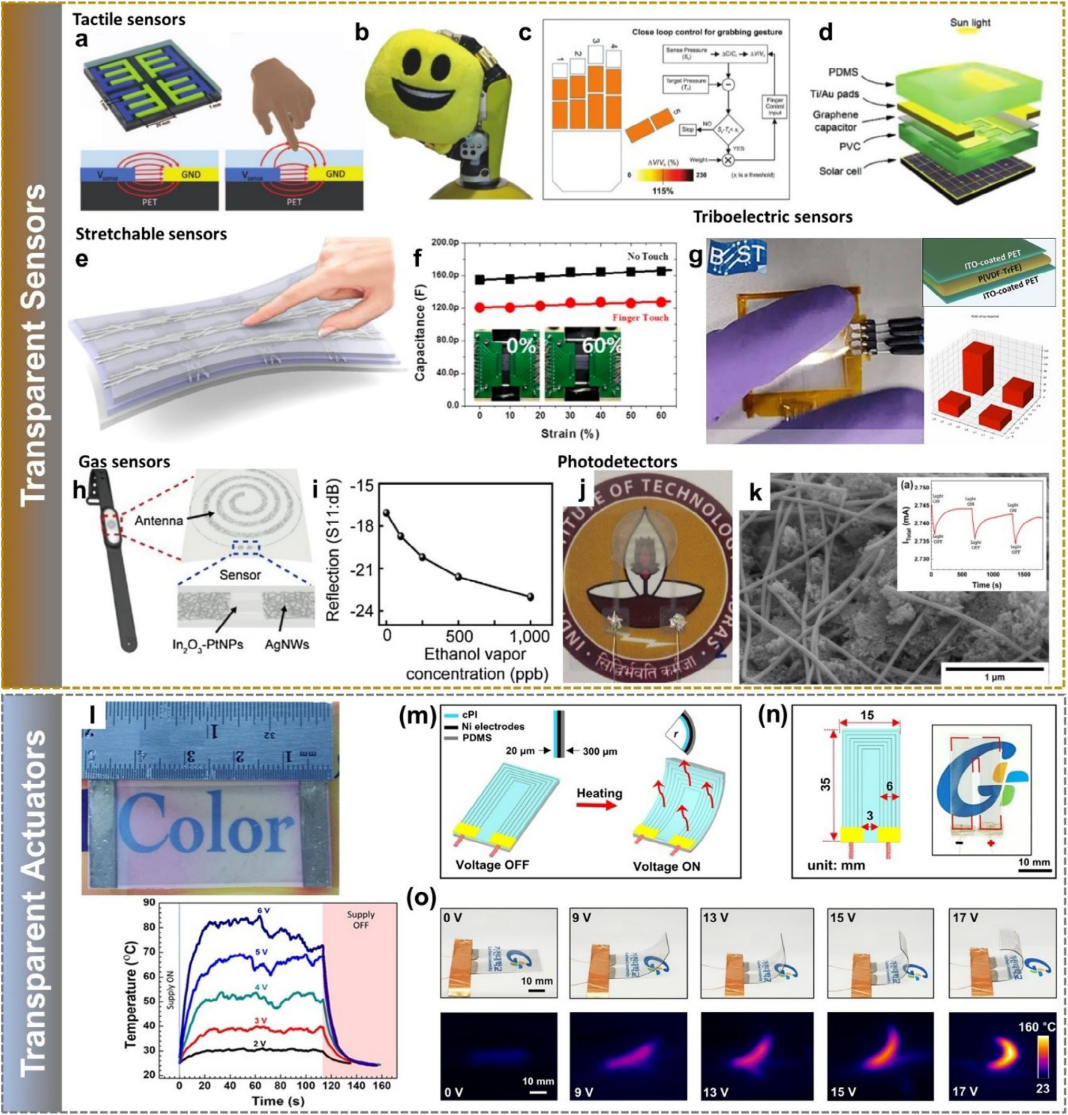
Transparent sensors and actuators. a) The schematic of a 2 × 2 touchpad matrix and the variation in its electric field in the absence and presence of the finger. Reproduce with permission.^[^ ^11b^ ^]^ Copyright 2019, IOP Publishing. b) The graphene‐based touch sensor integrating on the phalanges of a bionic hand. c) Closed loop implementation of the sensor to identify contact with soft objects. d) The concept of energy autonomous e‐skin by integrating transparent touch sensor with solar cell. Reproduced under the terms of CC‐BY.^[^ ^2a^ ^]^ Copyright 2017, John Wiley & Sons. e) Schematic of the stretchable transparent touch sensor. f) variation in the capacitance under different strain conditions without and with finger touch on top. Inset shows a single sensor at 0% and 60% stretching. Reprinted with permission.^[^ ^210^ ^]^ Copyright 2017, American Chemical Society. g) The piezoelectric‐based transparent touch sensor with the inset showing its structure and response to single touch. Reproduced with permission.^[^ ^213^ ^]^ Copyright 2023, IEEE. h) Schematic of a transparent flexible alcohol sensor integrated with an antenna for wireless communication installed on a smartwatch. Reproduce with permission.^[^ ^216^ ^]^ Copyright 2018, Elsevier. j) Photograph of the ZnO‐Ag NW nanocomposite PD placed on top of the IITM logo for the transparency demonstration. k) SEM image of the ZnO‐Ag NW nanocomposite with the inset showing the response to mobile flashlight illumination at 1 V bias. Reproduce with permission.^[^ ^122^ ^]^ Copyright 2021, IOP Publishing. l) Image of the printed transparent heater with the inset showing the heating profile at the center for different voltages. Reprint with permission.^[^ ^93^ ^]^ Copyright 2020, American Chemical Society. m) Schematic showing the working of transparent actuator before and after heating and n) photograph of the actuator. o) Photographs and corresponding IR images demonstrating the bending of the transparent actuator at various supply voltages. Reproduced with permission.^[^ ^217^ ^]^ Copyright 2024, Elsevier.

Sensors convert physical stimuli into electrical signals. A touch sensor is a commonly employed input device in mobile phones and other touch‐display devices to facilitate human–machine interactions. The primary requirement is that the sensor needs to be transparent so that it can be positioned on top of the display to sense the physical touch and, at the same time, allow the light from the display below to pass through. Several sensing mechanisms, including resistive, capacitive, piezoelectric, triboelectric, and infrared‐based, are used, with capacitive sensing being widely utilized due to its ease of fabrication, low power consumption, high sensitivity, and thermal stability features. Capacitive sensors with interdigitated and parallel structures are popular, as they detect touch by sensing variation in the electric field between the electrodes.^[^ ^209^ ^]^ An interdigitated touch sensor printed using Ag NW‐based on PET substrate with a PDMS dielectric was reported to achieve nearly 84% transparency.^[^ ^11b^ ^]^ A 2 × 2 flexible touchpad was also demonstrated to perform well under flat and bend conditions (**Figure** **17**a). Another application is in electronic skins (e‐skins), an artificial smart skin for robotics and prostheses to provide human‐like tactile perceptions. Graphene‐based e‐skin with an interdigitated structure was demonstrated, patterned using a computer‐controlled cutting blade on PVC substrate and PDMS as the dielectric.^[^ ^2a^ ^]^ This e‐skin exhibited high sensitivity over a wide pressure range, detecting a minimum pressure of 0.11 kPa up to 80 kPa with a sensitivity of 4.3 Pa^−1^. It was demonstrated by integrating on the phalanges of a bionic hand (Figure 17b), responding to touch feedback to grab soft objects (Figure 17c). An interesting concept of energy‐autonomous tactile skins was also illustrated with a solar cell on the backplane of the touch sensor, enabling battery‐free operation (Figure 17d). Recently, in the same direction, transparent touch sensors were integrated with flexible organic photovoltaics (OPV), where the OPV not only generates power but also performs shadow sensing for gesture recognition and proximity sensing.^[^ ^2e^ ^]^ It helps safer interactions and manipulations with better resource management, enabling the robots to “feel” and “see” simultaneously. Looking forward, the opaque solar cell can be replaced with a transparent and stretchable solar cell to realize a completely transparent autonomous e‐skin. Additionally, a stretchable 5 × 5 arrays of transparent capacitive touch sensors on PDMS substrate with a parallel structure, using Ag NW/GO hybrid parallel electrodes with a PU dielectric, has been developed for futuristic stretchable electronics (Figure 17e).^[^ ^210^ ^]^ The lower Poisson's ratio of PU dielectric compared to that of the PDMS substrate prevents changes in capacitance due to the sensor stretching (Figure 17f). The transparency of inkjet‐printed 70 nm thick amorphous ITO film on colorless polyimide substrate has been used for the development of invisible touch sensors for a security access system, demonstrating a simple yet promising application.^[^ ^211^ ^]^ The sensors discussed so far are capacitive‐based and hence, require an external power supply for the operation. Self‐powered sensor, such as triboelectric and piezoelectric, sensors are energy‐efficient alternatives.^[^ ^212^ ^]^ A triboelectric tactile sensors was reported with all the layers of the device being transparent, including the fluorinated ethylene propylene substrate used as the contact layer, Ag NW as the electrode material, and encapsulated PDMS, achieving 89% transparency.^[^ ^178^ ^]^ Transparent piezoelectric materials, such as PVDF, are promising alternatives for self‐powered pressure sensors. A transparent piezoelectric‐based touch sensor array was demonstrated using P(VDF‐TrFE) as the active material, stacked between ITO‐coated PET sheets (Figure 17g).^[^ ^213^ ^]^ These piezoelectric sensors could be extended into smart plasters, allowing wound monitoring without removing the plaster. Such self‐powered sensors help reduce power requirements and, therefore, lightweight sensing applications by eliminating bulkier charge storage devices. However, long‐term reliability, stability, and efficient circuit readout design are the main challenges associated.^[^ ^214^ ^]^

A transient transparent strain sensor was recently demonstrated, utilizing screen‐printed Ag NWs on agarose/glycerol gel substrates, designed to be in wearable form factor to monitor various biological activities.^[^ ^215^ ^]^ This device was capable of repeatedly measuring up to 20% strain, with a maximum resistance variation of 55%. Additionally, screen printing of serpentine Ag NWs electrodes was demonstrated for wearable applications, including the measurement of ECG and EMG signals and for haptic control. Upon reaching the end of their lifecycle, the gel‐based substrate biodegrades naturally, and the Ag NW electrodes can be conveniently recycled. These wearable electrodes are promising for sustainability, especially since their applications typically involve single‐use or limited reusability.

Transparent and flexible physical sensors that can sense parameters such as temperature and humidity were also widely employed for wearable and e‐skin applications.^[^ ^218^ ^]^ Recent trends include multi‐functional sensors that measure more than one physical parameter. An example in this direction is a transparent (≈89%) and flexible multifunctional sensor array that combines a capacitive fingerprint sensor array with tactile pressure and finger skin temperature detection capabilities.^[^ ^219^ ^]^ A parallel plate capacitive sensor with Ag nanofibers‐Ag NW hybrid electrodes and SiO_2_ dielectric for fingerprint sensing, an IGZO channel TFT transistor for pressure sensing, and PEDOT:PSS film for temperature sensing were fabricated on transparent polyimide substrate to form the multiplexed sensor. The demonstration is an encouraging example of the recent developments in the field and similar transparent and flexible multi‐functional sensors that have tremendous potential to meet the demand for futuristic e‐skins.^[^ ^220^ ^]^

Optically transparent chemical sensors are not as prevalent as physical sensors, but they offer the advantage of being “invisibly” installed anywhere for efficient detection. An example is the highly transparent (>93%) and flexible MoS_2_/rGO composite NO_2_ sensor.^[^ ^221^ ^]^ This sensor works by exchanging electrons during the interaction of the NO_2_ gas with the composite, resulting in a change in resistance that can be used to detect the gas. The sensitivity can be further improved using measures such as increasing the sensing surface area with free‐standing hollow AZO nanofibers.^[^ ^222^ ^]^ Real‐time wireless sensing of alcohol vapor was demonstrated using In_2_O_3_‐Pt NP hybrid nanostructures and Ag NWs as electrodes on a polyimide substrate (Figure 17h shows the schematic of a transparent flexible alcohol sensor integrated with an antenna for wireless communication, installed on a smartwatch).^[^ ^216^ ^]^ The 91% transparent sensor has an excellent response to ethanol vapor at concentrations less than 95 ppb, maintaining stable sensitivity over 155 days of operation and temperature range from −40 to 125 °C (Figure 17i). Implementing these sensors is extremely challenging, as both the electrical contacts and wiring connections must be transparent in addition to the active substance.

A photodetector (PD) converts the incident photons into an electrical signal, typically consisting of a semiconducting absorber layer to generate carriers (electrons and holes) based on the incident light intensity and highly conducting contacts to collect the carriers. A transparent (>70% transparency) and flexible PD has been reported with Ni/ZnO junction as an absorber and Ag NW electrodes on the colorless PI substrate.^[^ ^223^ ^]^ The transparent PD exhibited ultra‐high responsivity of 1.46 × 10^4^ A W^−1^ at 365 nm and quick response with <1 ms rise time and 2.5 ms fall time at −3 V bias. Another example is 44% transparent NiO/TiO_2_‐based self‐operational photodetector that works based on the photovoltaic effect without needing any external biasing, utilizing the built‐in potential for the charge separation, collected in Ag NW and FTO electrodes.^[^ ^224^ ^]^ Although self‐powered, this device needs a major boost in optical transparency. A printable composite ink of ZnO–Ag NW was also demonstrated to function as a photodetector (Figure 17j shows the transparent PD and Figure 17k shows the morphology of the nanocomposite) with 77% transparency.^[^ ^122^ ^]^ Photogenerated holes in the ZnO move to the Ag NWs by the Schottky barrier field, modulating the current through the NWs. The single‐layer nanocomposite transparent and flexible PD represents a promising approach for large‐area fabrication via wet processing techniques. Another promising example is a highly transparent (96%) PD utilizing single crystalline Si nanomesh structures.^[^ ^11e^ ^]^ Flexible and transparent transient PD has also been demonstrated on biodegradable cellulose substrates, highlighting the potential for low‐cost, ecofriendly disposable sensor systems.^[^ ^225^ ^]^

#### 5.2.2 Heaters and Actuators

Transparent heaters and actuators are in high demand for applications in invisible robots, haptic displays, and biomedical fields.^[^ ^226^ ^]^ In addition to their functionality and aesthetics, these devices offer the advantage of allowing visibility of underlying internal organs, which is particularly beneficial in the biomedical field.^[^ ^217^ ^]^ They can also create a camouflage effect to easily adapt to the surrounding conditions. TC materials are widely used to fabricate transparent and wearable heaters. When a large current flows through a material, heat is generated via the Joule effect,^[^ ^93^, ^227^ ^]^ which can be harnessed for various applications such as defrosters and anti‐foggers on displays, solar panels, window panes, as well as in therapeutic devices.^[^ ^228^ ^]^ Printed Ag NW‐PEDOT:PSS nanocomposite‐based transparent and flexible heater is a typical example, as shown in Figure 17l.^[^ ^93^ ^]^ Additionally, transient transparent heaters were also reported.^[^ ^229^ ^]^ The same heating mechanism can also be employed to create imperceptible actuators for transparent soft robotics^[^ ^230^ ^]^ and displays.^[^ ^231^ ^]^ Ni‐based directional transparent shape morphing actuator has been demonstrated (Figure 17m,n).^[^ ^217^ ^]^ The device consists of a three‐layer stack, where the Ni‐based transparent heater is sandwiched between two polymers having a significant mismatch in linear thermal expansion coefficient, PDMS, and colorless polyimide (PI) which have comparatively high and low thermal expansion coefficients, respectively. When a DC voltage is applied, the Ni film generates heat through resistive heating, causing the surrounding polymers to expand thermally. The soft actuator bends towards the PI (low thermal expansion) side due to the thermal expansion mismatch (Figure 17o). Ag NW‐based actuators also reported for various soft robotic applications, such as a transparent gripper, a venus flytrap, and a transparent walking robot, have been demonstrated.^[^ ^1^, ^226^ ^]^ However, the mechanical force that can be applied with similar actuators might be limited. Thermally actuated liquid crystal elastomer‐based tubular actuators, capable of lifting a weight of up to 3.92 N with a 38% deformation from their starting length, have been demonstrated.^[^ ^232^ ^]^ These actuators offer large actuation force and more extensive deformation with less voltage. Additionally, a dielectric elastomer actuator using an Ag NW‐SWCNT hybrid electrode, capable of achieving maximum areal strain of 146% has been reported.^[^ ^233^ ^]^ CNT improves device performance by effectively distributing thermal and electrical stress and increasing the effective area of the electrodes. Other stimuli, such as light, electric field, humidity, and pH have also been explored for actuation beyond electrical and thermal methods.^[^ ^234^ ^]^ More examples include 3D printed actuators, made from bio‐derived and biodegradable hydrogel materials, to realize sustainable soft robots, particularly for marine applications.^[^ ^235^ ^]^ Calcium‐alginate hydrogels derived from biodegradable brown seaweed, which is both safely edible and digestible by marine organisms, are utilized in their manufacturing. Although the optical transparency of these actuators needs to be studied more, such soft robots hold significant potential for deployment in marine ecosystems, with an additional advantage of not contributing to ocean pollution.

### 5.3 Interactive Displays

**Figure 18:**
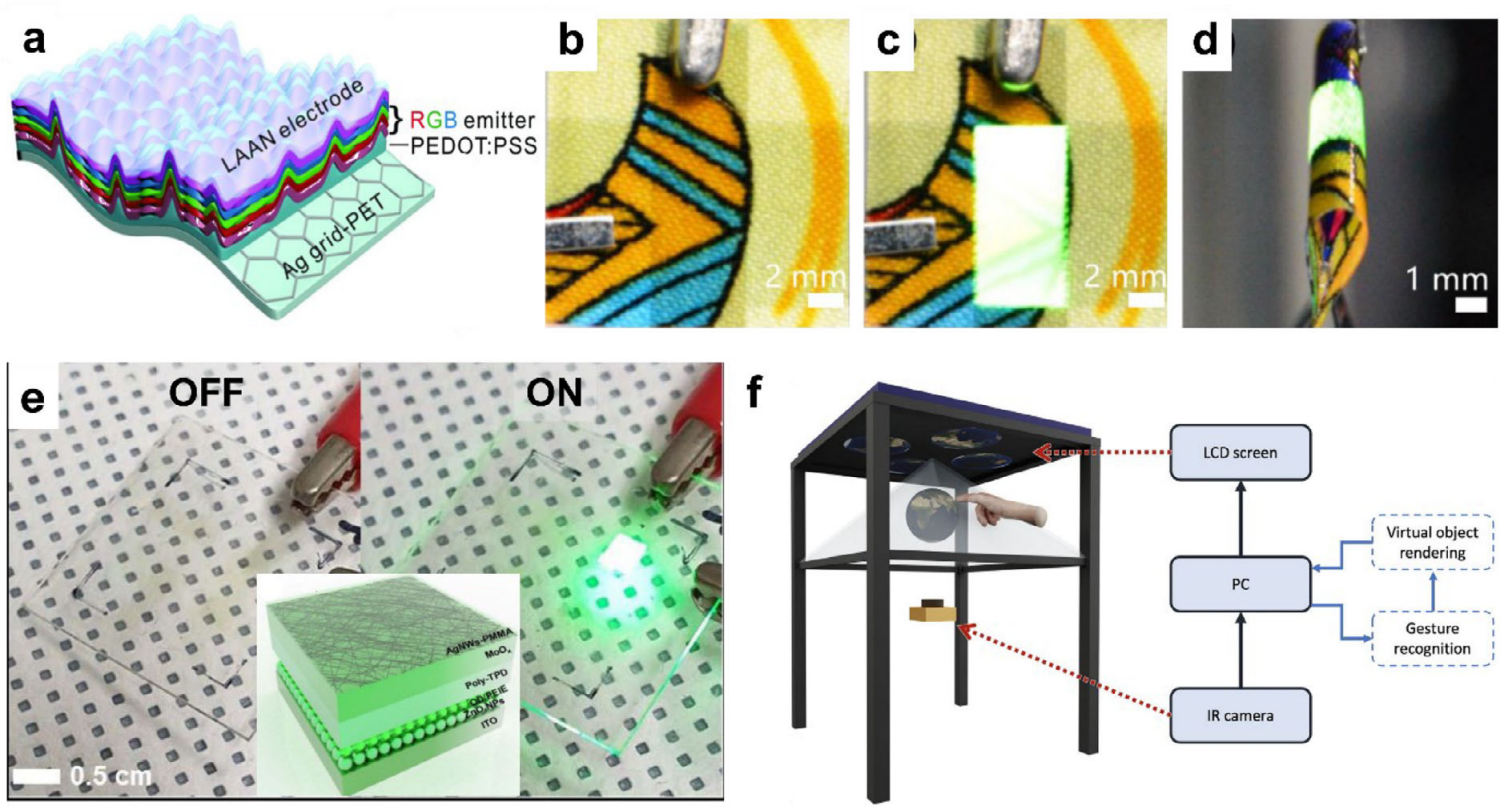
Transparent Interactive displays. a) Schematic of the structure of the transparent OLED with internal moth‐eye‐inspired nanostructure. Reproduced with permission.^[^ ^238^ ^]^ Copyright 2018, John Wiley and Sons. Photographs of the fabric display at a driving voltage of b) 0 V, c) 7 V, and d) under 1 mm radius bend condition. Reproduced with permission.^[^ ^239^ ^]^ Copyright 2020, Elsevier. e) Photograph of the QLED without and with the voltage. Inset shows the structure of the QLED. Reproduced with permission.^[^ ^240^ ^]^ Copyright 2019, American Chemical Society. f) Illustration of the 3D pseudo‐holographic display and its basic operation's block diagram. Reproduced under the terms of CC‐BY.^[^ ^3b^ ^]^ Copyright 2020, John Wiley & Sons.

Displays are an inevitable component in the visual interface of every electronic device and an indispensable part of the modern world. A transparent display projects information while allowing users to see through to the surroundings and interact using integrated transparent touch sensors. Such displays can be installed in the windshields of automobiles and airplanes for safer driving, and as head mount displays on eyewear.^[^ ^236^ ^]^ Displays technology varies from color filters that selectively pass the wavelength from a white backlight to passive matrix electronic inks and emitting technologies such as organic (OLEDs) and quantum‐dot light‐emitting diodes (QLEDs).^[^ ^231a^ ^]^ A notable advancement was made by LG display R&D center with the fabrication of a 77‐inch transparent and flexible OLED display with ultra‐high definition that can roll up to a radius of 80 mm, although it exhibited limited transparency of 40%.^[^ ^237^ ^]^ The major challenges in developing transparent displays include realizing efficient TC electrodes that facilitate a constant current supply across the active area while allowing generated light to pass through. Achieving effective TC electrodes on both the top and bottom is crucial for fully transparent displays. A 70% transparent flexible OLED with efficient warm white light emission from both sides was demonstrated,^[^ ^238^ ^]^ featuring a Ag‐grid/PET composite electrode structured similar to moth‐eye nanostructure for broadband and angle in‐dependent emission, with PEDOT:PSS coated on top to enhance the hole injection into the organic electrophosphorescent triple emitter layers (**Figure** **18**a). A maximum of 72.4% EQE was reported with 168.5 lm W^−1^ power efficiency and a color‐rendering index over 84. A fabric‐based flexible and transparent OLED demonstrated 74.22% transmittance using ultra‐thin metal films as electrodes.^[^ ^239^ ^]^ The display was constructed on a smooth spin‐coated polymer film transferred from glass to nylon fabric for surface smoothening and planarizing. Figure 18b,c shows the fabric display turned OFF and ON, respectively, confirming good optical transparency by the clear visibility of the fabric's color and pattern. The display functioned at a 1 mm radius bending (Figure 18d). Additionally, a transparent, highly efficient QLED was developed without any vacuum deposition technique, achieving a maximum total luminance of 27 310 cd m^−2^ and current efficiency of 45.99 cd A^−1^.^[^ ^240^ ^]^ Figure 18e shows the photograph of the QLED without and with the voltage, and the inset shows QLED structure. A biodegradable electrochromic display has also been demonstrated using PEDOT:PSS‐based electrochromic layer, gelatin‐based electrolytes, and Au electrodes—exhibiting transparent and deep blue states.^[^ ^241^ ^]^ These displays exemplify sustainability by combining a design based on biodegradable materials, which allows for eco‐friendly degradation at the end of life, with printed electronic fabrication that minimizes material waste through direct patterning.

Modern displays are expected not to be limited to visual information but should have surface texture rendering for an enhanced interactive experience. Embedding haptic feedback with visual displays has significant applications in automobiles, as displays for the visually disabled, and in interactive virtual reality applications.^[^ ^242^ ^]^ Soft elastomer actuators with Ag NW electrodes created a transparent haptic interface with customizable surface textures.^[^ ^243^ ^]^ Periodic rectangular void patterns were created on PDMS‐Ecoflex mixed elastomer, and these void regions could be vertically displaced by a controlled electrostatic force on Ag NW electrodes, successfully demonstrating various surface profiles from smooth to rough. Piezoelectric‐based transparent haptic devices have also been reported recently.^[^ ^244^ ^]^

Commonly used touch‐haptic‐visual‐display technologies are typically planar, such as 2D, due to the limitations in electronics manufacturing and packaging technologies. A simple and cost‐effective 3D interactive display was developed by combining pseudo‐holographic display technology with frustrated total internal reflection (FTIR) based touch sensing on a four‐sided transparent pyramidal surface (Figure 18f).^[^ ^3b^ ^]^ LCD screens positioned above the pyramid produced midair floating 3D illustrations using Pepper's ghost projection scheme. These 3D illustrations could change images, rotate by swiping, and zoom by pinch operations. Additionally, the group demonstrated interfacing an air‐based haptic feedback system with pseudo‐holographic display to deliver the sensation of touch, making the technology more promising.^[^ ^3c^ ^]^ This 3D display allows users to view and feel the virtual objects in mid‐air without the need for any headgear assistance, providing a futuristic virtual reality system capable of creating a feedback‐capable virtual environment.

### 5.4 Energy Devices

Solar energy is one of the most abundant energy sources, a solar cell facilitates the conversion of sunlight into electrical energy. A solar cell typically consists of a semiconducting material that generates electron‐hole pairs by absorbing photons through the photovoltaic effect.^[^ ^46^ ^]^ The amount of photocurrent generated is proportional to the number of photons absorbed, and hence, increasing the surface area is the solution to harness more energy. Transparent solar cells allow installation on any surface without visual hindrance, facilitating large‐area applications and space utilization. They can be installed on curved surfaces, such as vehicle windowpanes and wearables, by making them flexible.

**Figure 19:**
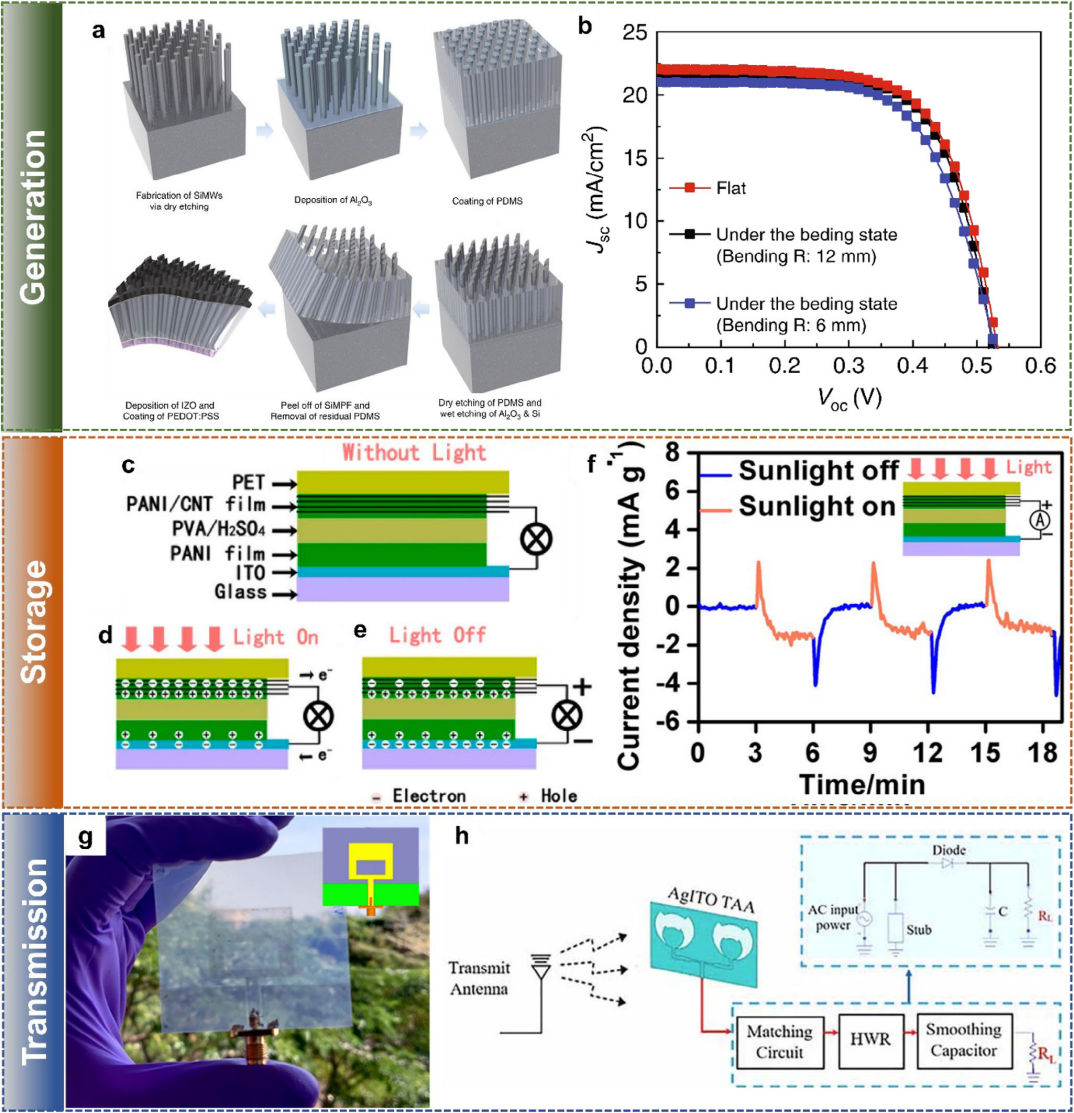
Energy Applications. a) Schematic showing the fabrication process of the Si MW‐based transparent and flexible solar cell. b) The *J*–*V* characteristics of the Si MW‐based transparent solar cell under various bending states, confirm the good flexibility of the device. Reproduced with permission.^[^ ^4c^ ^]^ Copyright 2019, Springer Nature. Schematic showing the c) structure and the working mechanism of the photo‐supercapacitor d) under illumination and e) when the light turned‐OFF. f) The short circuit current of the photo‐supercapacitor without external bias voltage under cyclic AM1.5 solar illumination. Reprinted with permission.^[^ ^29^ ^]^ Copyright 2015, American Chemical Society. g) Ag NW‐PEDOT:PSS‐based printed transparent and flexible Wi‐Fi antenna. Reprint with permission.^[^ ^93^ ^]^ Copyright 2020, American Chemical Society. h) RF energy harvesting system implementation diagram. Reproduced under the terms of CC‐BY.^[^ ^247^ ^]^ Copyright 2021, IEEE.

The concept of a transparent solar cell presents a challenge; as transparency requires materials to allow photons to pass through without absorption, conflicting with the photovoltaic process that relies on photon absorption for energy generation. Various strategies have been employed to make a solar cell transparent; i) controlling the shape of the nanostructures, ii) by optimizing the thickness of the films to trade‐off between transparency and energy conversion efficiency, and iii) allowing the visible light to pass through while absorbing the UV and near IR spectrum for energy conversion. A flexible and color‐neutral transparent solar cell using free‐standing n‐type Si microwires (MWs) embedded in PDMS has been reported.^[^ ^4c^ ^]^ Flat tip Si MWs were fabricated by deep reactive ion etching, then embedded and transferred into PDMS. IZO was deposited as the top transparent electrode, and PEDOT:PSS at the exposed MW end to form a hetero p‐n junction solar cell. **Figure** **19**a shows the fabrication procedure. The solar cell exhibited a power conversion efficiency (PCE) of 8.07% with 10% optical transparency. The transparency can be varied between 10% and 55% by increasing the spacing between the MWs (reducing active device density and thereby the absorption area), but by sacrificing the PCE. The device performance remained stable under bending conditions (Figure 19b), confirming its flexibility. Another example is a digitally printed flexible transparent organic solar cell with a PCE of and transmittance of 45%.^[^ ^245^ ^]^ This solar cell has an inverted structure with Ag NWs as the TC electrode, PEDOT:PSS as the hole transport layer, ZnO as the electron transport layer, and P3HT:PCBM active layer on PET substrate. An ultra‐flexible and light‐permeable organic solar cell with 12.04% PCE and 20% average visible transmittance using a near IR alloy acceptor (PM6:Y6:C6) demonstrated low incident light angle dependence, beneficial for complex 3D curved surface applications.^[^ ^246^ ^]^ It is evident that PCE and transparency are often at odds, but researchers are working to create solar cells that are both decently transparent and efficient. The expected transparent solar cell integrates two distinct structures: one with a continuous design that absorbs non‐visible wavelengths to generate energy while remaining transparent to visible light, and another with a low‐density, structurally transparent design that converts visible light into energy. This combination maximizes energy production across the entire light spectrum while maintaining transparency.

In addition to efficient energy generation, effective energy storage is crucial for the practical application of transparent electronics. Lightweight, optically transparent, and flexible power storage devices are essential for realizing fully transparent electronics. Because of their high storage density, longer life span, quick charge/discharge time, and maintenance‐free operation, supercapacitors are considered as the next‐generation energy storage source with a wide range of applications in portables, wearables, e‐skins, and health equipment. The structure of the supercapacitor typically consists of a solid‐state electrolyte sandwiched between two electrodes, which is a combination of a current collector and an active electrode material. All layers need to be flexible and transparent. A hierarchically nano‐branch structured Ni@MnO_2_ freestanding electrode‐based supercapacitor with PVA/LiCl gel electrolyte has also been reported.^[^ ^248^ ^]^ This supercapacitor exhibited an areal capacitance of 19.65 mF cm^−2^ at a scan rate of 0.01 V s^−1^ and 77% transparency. It also showed a long cycling life with 98.6% retention after 10 000 cycles, and good mechanical flexibility, preserving the capacitance after repeated folding and at various bending angles. An asymmetric supercapacitor has been reported with Ag NW@NiCo/NiCo(OH)_2_‐based cathode, Ag NW/graphene hybrid anode, and LiClO_4_/KOH/PVA gel electrolyte.^[^ ^94^ ^]^ The asymmetric electrode structure extended the voltage window to 1.2 V, boosting energy density and specific capacitance, achieving an areal capacitance of 9.6 mF cm^−2^, an energy density of 3.0 W h kg^−1^ and a power density of 2.6 W kg^−1^. Recently, inkjet‐printed MXene‐based 73% transparent supercapacitors demonstrated an areal capacity of 192 µF cm^−2^ at a scan rate of 2 mV s^−1^, retaining 100.8% of the initial capacitance after 180^○^ bending, which is promising.^[^ ^249^ ^]^ Additionally, an 85% transparent micro‐supercapacitor was demonstrated by direct ink writing.^[^ ^250^ ^]^ Such supercapacitors are ideal for localized power storage for sensors with high density, especially in wearable and e‐skin applications.

Multi‐functional supercapacitors have recently gained significant interest. One notable example is a demonstrated battery‐supercapacitor hybrid device.^[^ ^251^ ^]^ This PET/PEDOT:PSS/o‐MoO_3_/sulfuric acid gel device has >70% transparency, an areal capacitance of up to 2.99 mF cm^−2^, columbic efficiency of 99.7% over 2500 cycles, and high energy and power densities. The hybrid battery‐supercapacitor behavior is attributed to the direct integration of instantaneous power enabled by high surface area PEDOT:PSS doped with sulfuric acid facilitating proton intercalation with o‐MoO_3_. The ionic and electronic conducting PEDOT:PSS is an active layer for storing the ionic species and as a current‐collecting electrode. H^+^ protons intercalate into o‐MoO_3_ with voltage application, resulting in a color change for the negative electrode, providing additional electrochromic functionality. Another example includes the integration of flexible organic photovoltaics of 13.6% PCE with an MXene supercapacitor with a volumetric capacitance of 502 F cm^−3^ to realize a flexible semi‐transparent (33%) photovoltaic supercapacitor, fabricated entirely through solution processing.^[^ ^252^ ^]^ Both photo‐active layer and capacitive structure were combined in a single device for self‐charging under illumination and self‐storage of the generated photocarriers. PANI thin films were used as the photoactive and pseudocapacitive layers, CNT and ITO as electrodes, and PVA/H_2_SO_4_ gel as the solid electrolyte, as shown in Figure 19c. When illuminated, photocarriers were generated in the absorber PANI (Figure 19d). Due to the resistance variation in CNT and ITO, a net current will flow through the device, and charge the supercapacitor. The photovoltaic effect stops when the light is OFF, but the generated charge remains in the capacitor (Figure 19e). Figure 19f shows the short circuit current density of the device.^[^ ^29^ ^]^ More hybrid devices are anticipated to meet diverse energy‐generation and storage requirements in the future.

Antenna permits wireless energy transmission as electromagnetic radiation and is an indispensable part of every communication system. Making the antenna transparent allows integration on any surface, including windows, eyeglasses, automobile window shields, displays, and photovoltaics, without any visual blockage or affecting the performance of underlying devices.^[^ ^253^ ^]^ A CPW‐fed dual‐band Wi‐Fi antenna printed with Ag NW‐PEDOT:PSS nanocomposite ink on PET substrate is a promising example (Figure 19g).^[^ ^93^ ^]^ With over 77% transparency, it exhibits comparable radiation performance to commercial antenna and functions under flexible conditions. Reversibly stretchable Ag NW/PDMS^[^ ^254^ ^]^ and screen‐printed Ag NW/PET^[^ ^255^ ^]^ antennas have also been reported. Long, rigid antennas can be replaced with flexible, invisible antennas that perform better.^[^ ^93^ ^]^ Due to the shadowing and scattering effects from metallic areas, antennas cannot be efficiently placed inside automobiles. However, since a significant portion of the automobile is covered with glass, a transparent antenna can be effectively positioned on the windshield, rooftop, or mirrors. Another example includes a transparent ultra‐wideband multiple‐input multiple‐output antenna fabricated using FTO and ITO for automotive communication.^[^ ^256^ ^]^ Radio‐frequency (RF) energy harvesting is a prominent active research area for wirelessly powering devices. A transparent antenna array for energy harvesting in 5G mid‐range of frequencies was designed, capable of harvesting 0.308 V DC for received power of −5 dBm at 5.8 GHz, with a maximum power conversion efficiency of 53.5% (Figure 18h), promising for “invisible” RF energy harvesting.^[^ ^247^ ^]^

Next‐generation transient energy devices could offer a more environmentally friendly alternative for energy production, storage, and transmission. However, managing the end‐of‐life disposal of energy devices such as solar cells and batteries remains a significant challenge. Implementing new properties into a multifunctional device requires all the components to be flexible, transparent, and degradable while maintaining the intended functionalities. This is highly challenging from a materials standpoint.^[^ ^257^ ^]^ For instance, in energy storage, all the components, including the active materials, current collectors, electrolyte/separator, and battery packaging, need to be flexible, transparent, and degradable, while simultaneously preserving electrochemical performance, operational safety, and longevity. Although biodegradable photovoltaics^[^ ^258^ ^]^ and batteries^[^ ^259^ ^]^ have been widely reported, they typically lack optical transparency. More interdisciplinary research efforts are needed in the coming years to achieve transient and transparent devices.

## 6 Challenges and Opportunities

Flexible and transparent electronics have received enormous attention because of their invisible nature, lightweight, low‐cost, and conformability advantages compared to conventional rigid electronics. Numerous materials have emerged, and novel fabrication technologies have been developed, significantly shaping the flexible transparent technology in its current form. Still, many challenges remain to be addressed. This section discusses some of the challenges that will need greater attention in the near future and how the field could evolve in the long term.

### 6.1 Sustainability and End‐of‐Life Challenges

Technological advancements are essential for the benefit of society and this should happen without jeopardizing the ability of future generations to perform well. This calls for sustainable use of resources, their short‐term recovery, and a lower ecological footprint. While there awareness related to sustainability and recyclability of electronics is growing; there is still a considerable amount of toxic and hazardous byproducts produced. The commonly used transparent and flexible substrates, such as PET, polycarbonate, and polyimide, are transparent plastics and are widely used due to their low cost, lightweight, easy handling, and favorable mechanical properties. Many of these materials are either non‐biodegradable or non‐renewable.^[^ ^70^, ^260^ ^]^ Moreover, discarded plastics may degrade into micro and nano‐plastics that cannot be degraded by micro‐organisms, creating hazardous situations for humans, marine life, animals, and the environment by entering the food chain.^[^ ^261^ ^]^ As the lifespan of flexible and transparent devices becomes shorter and cheaper, partly because of human habits of replacing electronics products too frequently, there is a high probability that these materials could contribute to severe ecological issues, raising series sustainability concerns.^[^ ^67^ ^]^

Another challenge is the availability of materials, such as the indium of ITO, a rare earth element with an average abundance of only 0.02 ppm.^[^ ^262^ ^]^ The majority of indium consumed worldwide, >65%, is dedicated to ITO production.^[^ ^14a^ ^]^ However, the target utilization of ITO in sputter coating for fabricating TC electrodes in displays and photovoltaics is only 15%, indicating that a significant portion of indium is wasted without any use.^[^ ^14a^ ^]^ While the recovery of indium has been extensively studied, it often requires non‐ecofriendly chemical reactions for separation, recovery, and refining.^[^ ^14a^ ^]^ In addition, many nanomaterials need to be handled in a controlled manner; otherwise, they will be toxic and cause health concerns to the users. For example, graphene, a popular TC because of its good carrier mobility, can induce acute and chronic injuries to the tissues by intake and is potentially toxic if not adequately handled.^[^ ^263^ ^]^ The chemical precursors, organic solvents, and stabilizers used for ink formulations in printable electronics can cause environmental pollution, and some of them are even carcinogens.^[^ ^140^ ^]^ To address these environmental concerns, new alternative materials need to be explored.

The biodegradable materials discussed in Section 3 present promising alternatives, offering a viable solution for long‐term sustainability. Additionally, the use of rare earth elements should be minimized and replaced with other earth‐abundant materials. For instance, Fe or Si‐based NWs could serve as substitutes for indium‐based metal oxides in transparent conductors and semiconductors, respectively. In addition, the materials should be properly separated and recovered at the end‐of‐life of the devices whenever possible. End‐of‐life management must be considered from the initial device design steps, and the ecological impact should be analyzed in advance for better management. Fabrication routes should be more eco‐friendly, for example, maximizing water‐based printable ink formulations whenever possible^[^ ^264^ ^]^; otherwise, the solvents and stabilizers should be adequately processed to make them eco‐friendly before disposing to nature. More resource‐efficient manufacturing routes must be encouraged for better utilization of resources and minimizing waste. In this regard, the printing techniques discussed in section 4 are promising. Some of these printing technologies also allow dry and solvent‐free direct transfer of materials. Another solution for sustainable electronics is to extend the lifetime of the devices by designing them for easy repairability, i.e., eco‐design and reusing as much as possible.^[^ ^140^ ^]^

### 6.2 Performance Challenges

The main challenge associated with commercializing transparent electronics is its performance. The strength of conventional Si‐based electronics is high device performance, which has not yet been achieved with state‐of‐the‐art transparent flexible electronics. High‐performance devices need excellent carrier mobility together with suitable quality interfaces. The carrier mobility of the polymers and the metal oxides (suitable p‐type oxides are also lacking) has limitations. Generally, crystalline materials have better mobility due to less boundary scattering, and at the same time, they are more brittle and hence have low flexibility. The film thickness must also be restricted to minimize light absorption for transparent electronics since optical clarity is the primary desire, but it will increase the resistance. While figure of merit for optimizing thickness for TC applications have been defined; no such standards are available for transparent semiconducting and insulating materials, which demands more standardized definitions in terms of optoelectronic performance. Further, achieving good quality films with smooth surface profiles is impractical with solution‐based printing techniques, and the minimum feature size achievable has limitations. The low thermal budget requirements of the flexible plastic substrate further limit the annealing‐based methods for improved quality of the films. Structurally transparent materials will be a better option to cope with these challenges.

Emerging manufacturing techniques such as direct‐roll printing of nanostructures, and DEP‐based mesh of aligned metal NWs etc., discussed in Section 4, could be promising approaches for high‐performance flexible transparent devices.^[^ ^160^, ^265^ ^]^ These methods use nanostructures produced from conventional wafers or grown using bottom‐up approaches, which ensures the electronic grade quality of materials and, hence the development of high‐performance devices with flexible form factors. Being nano‐sized, the nanostructure devices can exhibit structural transparency combined with suitable ohmic TC materials. While such transparent devices have not been explored as much as others, the approach holds significant potential. Long metallic NWs with diagonal alignment, assisted by emerging techniques like DEP, could be used for electrical contacts.^[^ ^136^, ^138^ ^]^ By enabling the seamless fabrication of diverse functional components on a single substrate, advanced multi‐material and multi‐layer printing technologies hold great promise for the development of fully integrated transparent electronic devices with high throughput.^[^ ^266^ ^]^ The next generation of low‐power wearables and Internet of Things applications will benefit greatly from the high‐performance transparent CMOS chips and circuits once they are accomplished. Additionally, more advancements are anticipated in the design, modeling, fabrication, and testing of transparent, flexible analog, digital, and power electronic technologies.

### 6.3 New Opportunities in Transparent Flexible Electronics

The opportunities that can be provided by transparent flexible technologies are enormous, and far beyond making devices invisible. With invisibility and flexibility, users have additional options for device placement on a broad range of surfaces, including non‐planar surfaces without shape and location constraints. For example, a wide variety of communication technologies, such as AM/FM radio, Bluetooth, GPS, and RFID, present in automobiles require an antenna for the signal radiation. Due to scattering and screening effects exhibited by the metallic body of the automobile, the space available for antenna installation is limited. A transparent antenna can be installed on windshields, mirrors, and rooftop glasses without any visual hindrance, as these positions are away from the metallic body of automobiles for better efficiency. Research on high‐performance flexible and transparent antennas is limited. Similarly, transparent displays can be installed on the windshields for better user‐friendliness to the driver. They can make it interactive by adding transparent touch‐sensing technologies but need to ensure that the overall device stack has >80% transparency. A large surface of buildings and automobiles covered by glass can be made as a source of energy using transparent solar cells. Further, these could be combined with transparent antennas for RF‐solar harvesting and wireless power transmission. The PCE of the transparent solar cell is reported to be poor and truly an efficient transparent solar cell is lacking, which is a new area that needs to be explored. Transparent supercapacitors and batteries can also be incorporated for localized and distributed energy storage.^[^ ^267^ ^]^ In this regard, the self‐chargeable supercapacitor (section 5) is a promising device.^[^ ^29^ ^]^ Another potential area will be wearable electronics, the flexibility and transparency of these devices will aid the comfort of wearing without sacrificing performance, which is a primary concern in the field. Transparency enables sensors to be placed in the contact lenses and spectacles that can be self‐powered with piezoelectric,^[^ ^268^ ^]^ thermoelectric,^[^ ^269^ ^]^ and triboelectric generators^[^ ^270^ ^]^ and can be processed locally using transparent circuitry^[^ ^271^ ^]^ for better capability and intelligence. In the medical industry, smart, transparent bandages with multipurpose sensors will allow visual observance of wound healing progress and real‐time monitoring of cell regeneration. In effect, the opportunities are wide open, and the challenges lie at the cross‐section of attributes such as mechanical (flexible form factor), electrical (high mobility of charge carriers), and optical (high transparency) factors.

## 7 Conclusion

In conclusion, transparent flexible electronics have seen great advancements recently, and demand is anticipated to rise in the future. ITO, the commercially popular material famous for its good electrical conductivity and optical transparency, is gradually losing its significance, because emerging applications require electronics in flexible factors and its limited supply. These bottlenecks have paved the way for a wide variety of substitutes such as polymers, metallic nanowires, and carbon‐based materials with good mechanical flexibility. These materials have been reviewed in this article based on their suitability for flexible transparent applications. Large‐area deposition of quality films of these materials is always challenging. It remains to be seen how we may use some of the newly developed resource‐efficient manufacturing processes to produce transparent flexible electronics cost‐effectively and with minimal environmental impact. The applications of the transparent technology are enormous; the emerging flexibility and invisible features offer the freedom of device installation over a large non‐planar area, without being noticed, and hence utilized in developing transparent devices, circuits, sensors, actuators, and energy generation and storage. However, several challenges still remain to be addressed, especially on how to utilize collectively the innovative features (e.g., transparency with flexibility and conductivity) effectively to address the needs of various applications, how to improve the device performance and bring it on par with the conventional devices, how to minimize the impact on the environment during the manufacturing, and finally, how to dispose or recycle at the end of operational life. Opportunities are wide open, and we anticipate more innovations in new materials, and implementation of resource‐efficient fabrication strategies, leading to unexplored applications.

## Conflict of Interest

The authors declare no conflict of interest.
